# Fast and highly sensitive full-length single-cell RNA sequencing using FLASH-seq

**DOI:** 10.1038/s41587-022-01312-3

**Published:** 2022-05-30

**Authors:** Vincent Hahaut, Dinko Pavlinic, Walter Carbone, Sven Schuierer, Pierre Balmer, Mathieu Quinodoz, Magdalena Renner, Guglielmo Roma, Cameron S. Cowan, Simone Picelli

**Affiliations:** 1 Institute of Molecular and Clinical Ophthalmology Basel, Basel, Switzerland; 2 Department of Ophthalmology, University of Basel, Basel, Switzerland; 3 Chemical Biology and Therapeutics, Novartis Institutes for Biomedical Research, Basel, Switzerland

**Keywords:** Transcriptomics, RNA sequencing, Next-generation sequencing

## Abstract

We present FLASH-seq (FS), a full-length single-cell RNA sequencing (scRNA-seq) method with increased sensitivity and reduced hands-on time compared to Smart-seq3. The entire FS protocol can be performed in ~4.5 hours, is simple to automate and can be easily miniaturized to decrease resource consumption. The FS protocol can also use unique molecular identifiers (UMIs) for molecule counting while displaying reduced strand-invasion artifacts. FS will be especially useful for characterizing gene expression at high resolution across multiple samples.

## Abstract (web summary)

FLASH-seq speeds up high-sensitivity scRNA-seq while sparing resources.

## Main

Switching mechanism at the 5′ end of the RNA template (SMART) protocols, such as Smart-seq2 (ref. ^1^) (SS2), have shaped the single-cell transcriptomic field. Although more labor intensive and expensive than emulsion droplet methods, they allow a much deeper analysis of the transcriptome, enabling the characterization at single-cell resolution of splice isoforms, allelic variants and single-nucleotide polymorphisms (SNPs)^2,3^. SS2 remained the gold standard for many years thanks to its sensitivity, robustness and simplicity^4^. Recently, Hagemann-Jensen and colleagues developed Smart-seq3 (SS3), a new SMART sequencing (SMART-seq) protocol that includes UMIs to increase transcript quantification accuracy^5^. However, SS3 remains time-consuming, requiring careful fine-tuning to reach the appropriate balance between internal and UMI-containing reads.

In this study we introduce FS, a full-length scRNA-seq protocol capable of generating sequencing-ready libraries in less than 4.5 hours while offering unmatched sensitivity.

We established FS (Supplementary Fig. 1) by introducing several key modifications to SS2: we combined reverse transcription (RT) and cDNA preamplification (polymerase chain reaction) (RT-PCR), replaced the Superscript II reverse transcriptase with the more processive Superscript IV (SSRTIV) and shortened the RT reaction time, increased the amount of dCTP to favor the C-tailing activity of SSRTIV and boost template-switching reaction^6^ and replaced the 3′-terminal locked nucleic acid guanidine in the template-switching oligonucleotide (TSO) (prone to cause strand invasion ^7,8^) with riboguanosine.

**Fig. 1:**
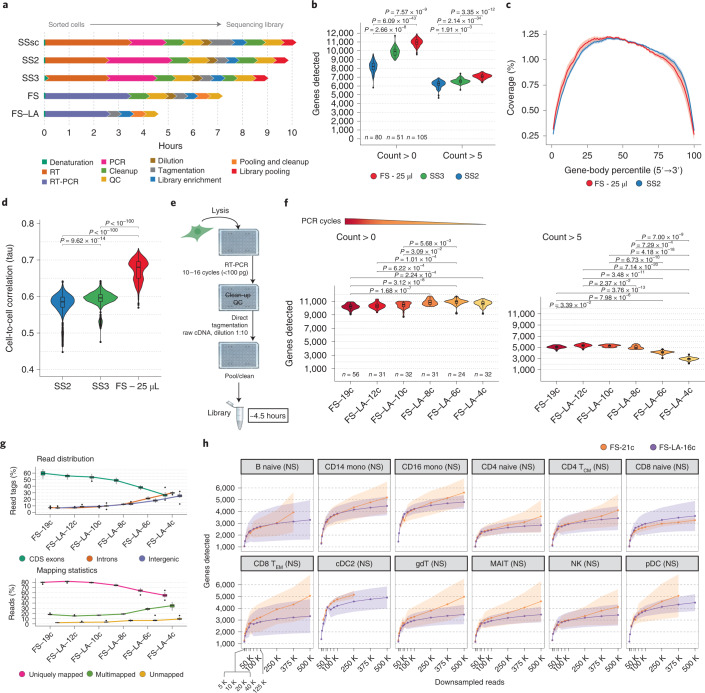
Overview of the FS and FS low-amplification (FS-LA) protocols. **a**, Estimated protocol duration to process a 96-well plate of HEK293T cells for the full-length scRNA-seq protocols used in this study. Steps are color-coded. QCs include concentration and size distribution measurements. SSsc, SMART-Seq Single Cell Kit (Takara). **b**, Number of genes detected in HEK293T cells processed with SS2 (*n* = 80), SS3 (*n* = 51) and FS (*n* = 105) at two read thresholds, with reads downsampled to 500,000 (= 500K) raw reads. **c**, Mean ± standard deviation (s.d.) gene-body coverage in HEK293T cells. **d**, Cell-to-cell Kendall’s tau correlations among gene expression in SS2 (*n* = 80), SS3 (*n* = 51) and FS (*n* = 105) using only genes expressed in all three methods (*n*_genes_ = 20,042). **e**, FS-LA workflow. The number of required PCR cycles is a function of the cell RNA content. **f**, Number of genes detected in HEK293T cells processed with FS (*n*_FS-19c_ = 56) or FS-LA (*n*_FS-LA-12c_ = 31, *n*_FS-LA-10c_ = 32, *n*_FS-LA-8c_ = 31, *n*_FS-LA-6c_ = 24, *n*_FS-LA-4c_ = 32) using 250 K downsampled raw reads. Gene detection threshold was set to >0 or >5 reads. **g**, Top panel shows the percentage of read tags mapped to exonic (= CDS exons), intronic or intergenic features in HEK293T cells processed with FS or FS-LA, measured using ReSQC. Bottom panel shows mapping statistics with the percentage of uniquely mapped, multimapped or unmapped reads for FS and FS-LA. **h**, Mean ± s.d. number of detected genes (>0 reads) per cell type in hPBMC samples. Only points supported by two or more cells are displayed. Some cells had insufficient coverage to be represented at each point. The difference between the number of genes in FS and FS-LA was evaluated at 125 K reads for each cell type using a Wilcoxon rank-sum test (two-sided, Bonferroni correction, adjusted *P* value). No statistically significant differences (NS) were observed (*P* > 0.05). MAIT, mucosal-associated invariant T cell; mono, monocytes; NK, natural killer; T_CM_, central memory T cell; T_EM_, effector memory T cell; gdT, gamma-delta T-cell; pDC, plasmacytoid dendritic cell; cDC2, conventional dendritic cell 2. A two-sided Dunn’s test was used, and Bonferroni corrected, adjusted *P* values are shown for **b**, **d** and **f**. Box plots in **f** and **g** show the median (center), 25th/75th percentile (lower/upper hinges), 1.5× interquartile range (whiskers) and outliers (points).

FS is 2–3.5 hours shorter than other methods (Fig. 1a and Supplementary Table 1) and detects more genes in HEK293T cells (Fig. 1b), regardless of the sequencing depth (Supplementary Fig. 2a,b). This higher sensitivity favors the capture of a more diverse set of isoforms and genes, especially protein-coding and longer genes (Supplementary Fig. 2c–g). The method also shows a typical full-length gene-body coverage and an improved cell-to-cell correlation (Fig. 1c,d). FS yields eight times more cDNA than SS2 and SS3 for the same number of PCR cycles (Supplementary Fig. 2h). The initial version of FS (full-volume reaction, 25 μl) also gave excellent results in cells with a low RNA content, such as human peripheral blood mononuclear cells (hPBMCs) (Supplementary Fig. 3a–c). Of note, we observed a higher proportion of intronic reads in SS3 than with the other protocols tested here (Supplementary Fig. 3d,e).

We then miniaturized FS to reduce costs and increase reaction efficiency^9–12^. The addition of a single RT-PCR mix minimizes evaporation thanks to the higher starting volume, making our protocol particularly suitable for automation and miniaturization.

Miniaturization did not increase the number of genes detected in HEK293T cells (Supplementary Fig. 2a,i) but boosted gene diversity and the number of those with a higher GC content (Supplementary Fig. 2d,g). In hPBMCs, the volume reduction led to a notable increase in the number of genes detected in CD14, CD16 monocytes and naive CD8^+^ T cells (Supplementary Fig. 3a–c). For practical reasons, we eventually settled on a 5-μl reaction volume.

Hundreds of reaction conditions/additives were tested to improve the FS protocol (Supplementary Discussion). As very few of them turned out to be beneficial, we concentrated our efforts on reducing the protocol duration instead. FS yields nanograms of cDNA after preamplification, but library preparation requires just a few picograms^13^. Leveraging this higher efficiency, we tested the limits of our system by varying the number of preamplification cycles and titrating the amount of Tn5 (Supplementary Fig. 4). Due to the minute amounts of cDNA generated after preamplification, we did not perform any purification or intermediate quality control (QC) but proceeded directly to tagmentation, saving an additional ~2.5 hours. The resulting FS-LA protocol is cheaper than FS and requires <1 hour hands-on time (Fig. 1a,e and Supplementary Table 1).

The quality of FS-LA libraries was correlated to the level of amplification but independent of the Tn5 amount (Supplementary Figs. 5 and 6). Performing too few PCR cycles resulted in a high proportion of genes supported by less than one read (Fig. 1f and Supplementary Fig. 5) and, despite an excellent gene-body coverage, a generalized lower data quality (Fig. 1f,g and Supplementary Figs. 6 and 7). A closer investigation revealed a random distribution of intergenic reads across the genome, including in centromeres, suggesting potential genomic DNA tagmentation (Supplementary Fig. 8).

The sufficient level of cDNA amplification depends on the cell RNA content. High-quality libraries from large cells (HEK293T) were obtained with 10–12 PCR cycles. In cells with lower RNA content (hPBMCs), 14–16 PCR cycles were required to generate data of comparable quality with the standard FS (21 cycles), including T cell antigen receptor (TCR) reconstruction (Fig. 1g,h and Supplementary Figs. 7 and 9).

In conclusion, FS can be performed using few cycles, thereby reducing the processing time to ~4.5 hours while still guaranteeing data quality comparable to longer experiments. A pilot test is recommended to determine the optimal number of cycles (see Supplementary Note 1).

FS and SS2 display similar robustness and flexibility^14,15^. To illustrate this, we modified FS by including UMIs into the TSO sequence (hereafter FS-UMI), as previously described^5^, thus generating both 5′ UMI-containing reads and internal reads without UMIs.

**Fig. 2:**
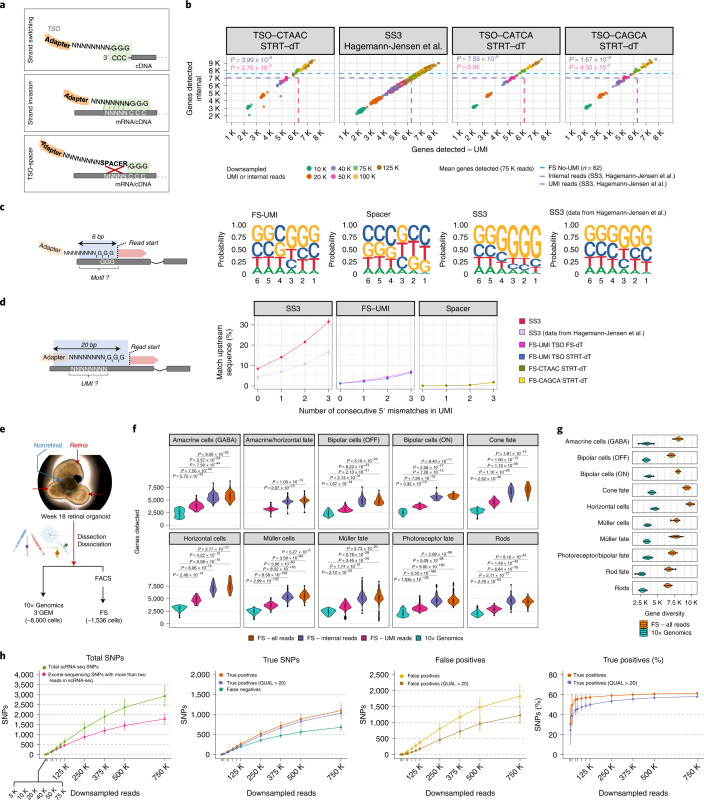
Strand-invasion events are mitigated in FS-UMI. **a**, Strand-switching reaction occurs at the end of cDNA (top); strand-invasion events are increased when the UMI and riboguanosine sequences are adjacent (middle) but are mitigated by a spacer sequence (bottom). **b**, Relationship between the number of genes detected in HEK293T cells using UMI and internal reads at several downsampled sequencing depths (*n*_SS3_ = 85, *n*_SS3_Hagemann-J._ = 101, *n*_FS-CTAAC_STRT-dT_ = 76, *n*_FS-CAGCA_STRT-dT_ = 16). Blue line denotes the mean number of genes detected in FS (*n*_FS-5μl_ = 85, 75 K reads). Purple/pink lines denote the mean number of genes detected in SS3 (Hagemann-Jensen et al.^5^, UMI = pink, internal = purple, 75K reads). Numbers of genes detected at 75 K reads were compared between cells from^5^ and the other conditions for both read types (two-sided Wilcoxon rank-sum test, Bonferroni adjusted *P* value, colored by read type). **c**, Nucleotide distribution in the 6-bp adjacent to the read start using 500 K randomly selected UMIreads (HEK293T). **d**, Mean ± s.d. deduplicated UMI reads (%) harboring a match between the UMI and the upstream sequence within 20 bp, with 0 to 3consecutive 5′ mismatches (*n*_SS3_ = 2,089,581, *n*_SS3_Hagemann-J._ = 13,511,157, *n*_FS-UMI-TSO_STRT-dT_ = 1,404,599, *n*_FS-CTAAC_STRT-dT_ = 9,964,516, *n*_FS-CAGCA_STRT-dT_ = 1,189,676,UMI reads). Colored by oligo-dT/TSO combination. **e**, Retinal organoid and experimental design. Retinal and nonretinal parts are highlighted. **f**, Genes detected (>0 reads) in representative cell types using 10x Genomics and FS-UMI (UMI, internal or both reads). Dunn’s test (two-sided, Bonferroni adjusted *P* value). Bipolar ON, ON-center bipolar cells; bipolar OFF, OFF-center bipolar cells. **g,** Gene diversity. Defined by resampling ten cells per cell type 100 times and calculating the number of genes expressed with >0 reads (both UMI/internal reads) in more than two cells. **h**, Mean ± s.d. detected SNPs at various downsampled read depths (*n*_FS_retinal_organoids _= 1,281). For each cell, exome-sequencing SNPs in transcribed regions (sequencing depth (= DP) > 2) as are used as reference. Totals SNPs, detected in FS-UMI (green, DP > 2) or in the reference (pink). True positive SNPs, detected in FS-UMI and the reference (orange, DP > 2; purple, DP > 2 & variant quality (= QUAL) > 20). False negative SNPs, reference SNPs not detected in FS-UMI (dark green). False positive SNPs, detected in FS-UMI but not present in the reference (yellow, DP > 2; brown, DP > 2 & QUAL > 20). True positive SNPs (%), percentage of true-positive SNPs detected among the reference SNPs. Box plots in **f** and **g** show the median (center), 25th/75th percentile (lower/upper hinges), 1.5× interquartile range (whiskers) and outliers (points).

When designing our UMI-containing TSO, we considered the possibility that the addition of eight random nucleotides upstream of the three riboguanosines (that is SS3-TSO) could favor strand invasion events (Fig. 2a)^7^. These artifacts could affect isoform detection and bias gene counts. We compared the SS3-TSO and FS-UMI-TSO, both carrying an -NNNNNNNN-rGrGrG sequence at their 3′ ends, to additional TSOs containing a 5-nt spacer sequence (-NNNNNNN-SPACER-rGrGrG), similarly to nanoCAGE (ref. ^7^) and the 5′ Next GEM kit from 10x Genomics. These new TSOs were tested in combination with STRT-seq2i-oligo-dT_31_ (ref. ^16^), SS3-oligo-dT_30_VN (ref. ^5^) or FS-oligo-dT_30_VN on HEK293T cells (Supplementary Figs. 10 and 11). The cDNA yield after preamplification was substantially affected by the choice of TSO (Supplementary Fig. 11a,b). The proportion of 5′ UMI reads varied from 10.3% to 43.7% (Supplementary Fig. 11c), highlighting the difficulty of striking the right balance between the two read types ahead of sequencing. Overall, all conditions displayed excellent mapping statistics and gene-body coverage (Supplementary Figs. 12,13 and 14a). FS-UMI detected more genes using internal reads (Fig. 2b and Supplementary Figs. 14b and 15a) and more unique molecules (Supplementary Fig. 15b) than SS3 when using UMI reads. Similar numbers of genes between the best FS-UMI conditions and SS3 were observed when taking UMI reads only (Fig. 2b and Supplementary Fig. 14b). In both protocols, the edit distance of UMI sequences assigned to the same gene did not differ from a random sampling of UMI reads (Supplementary Fig. 15c), suggesting a limited occurrence of UMI inflation events. At 250 K raw reads (=UMI + internal reads), FS-UMI detected on average ~8 ± 4.3% and ~18 ± 6.1% (mean ± s.d.) more genes and isoforms, respectively (pseudo-alignment, Supplementary Fig. 15d). Generally, UMI reads identified a lower number of unique genes than internal reads (−18 ± 9.6% (mean ± s.d.), 75 K reads, Fig. 2b and Supplementary Fig. 15e). Compared to internal-specific genes, UMI-specific genes are on average shorter, display a higher GC content and are enriched in protein-coding genes (Supplementary Fig. 15f–h).

We observed several elements suggestive of strand invasion when using a TSO without a spacer sequence (Supplementary Fig. 16). In fact, a ‘GGG’ motif was more often observed adjacent to the first base of deduplicated 5′ UMI reads (Fig. 2c). We also noted a perfect match between UMI and sequence upstream of the read in >4.25% of SS3 deduplicated 5′ UMI reads (Fig. 2d). When accounting for the partial 5′ match of the UMI and the upstream sequence, this value rose to >10.9% with two mismatches (Fig. 2d). The percentage of UMI matching the upstream sequence remained higher in SS3 compared to FS-UMI, when considering the presence of the GGG motif and spacer sequence in FS-UMI (Supplementary Fig. 16d). Moreover, SS3 showed a higher percentage of UMI reads mapping to intronic features and a decrease in 5′ untranslated region reads (Supplementary Fig. 13). Finally, many SS3 deduplicated 5′ UMI reads mapping to intronic regions were mapped in an antisense orientation to the gene reference (Supplementary Fig. 16e). The addition of a spacer sequence between riboguanosines and UMI appeared to prevent most of the strand-invasion events.

Finally, we compared FS-UMI with 10x Genomics (3′ Next GEM kit) to highlight the added value of using full-length RNA sequencing and further explore FS-UMI properties (Fig. 2e). We processed single cells derived from week 18 human retinal organoids either with FS-UMI (*n* = 1,536, mean ± s.d._raw_reads_ = 558 K ± 260 K) or 10x (*n* ≃ 8,000, μ_raw_reads_cellranger_ = 33.8 K reads).

For FS-UMI, we selected the TSO carrying the ‘CTAAC’ spacer, as it previously gave excellent results and the sequence ‘CTAACGGG’ was the least likely to match any transcriptomic or genomic sequences among all tested TSOs. Paired-end sequencing revealed the unexpected presence of UMI sequences in 5.26 ± 1.08% (mean ± s.d.) read 2, adjacent to a truncated 5′ adapter sequence (Supplementary Fig. 17a–c). Both FS-UMI read 2 UMI (R2-UMI) and regular read 1 UMI (R1-UMI) displayed similar properties (Supplementary Fig. 17c–i). We hypothesize that a tagmentation event occurs within the TSO sequence upstream of the UMI, randomly leaving a P5 (R1-UMI) or a P7 (R2-UMI) sequence (Supplementary Fig. 17a,d). This hypothesis is supported by the regularity of the cuts mirrored in R1/R2-UMI (Supplementary Fig. 17d) favoring a guanosine^17^ and by the fact that the minimal fragment length for a Tn5 cut has been reported to be ~40 bp^18^ (Supplementary Fig. 17d).

These 5′-tagmentation events in a UMI-containing TSO have two implications. First, UMI sequences could be lost during library preparation. Second, as the length of the 5′ adapter sequence gets shortened, the sequencing of uninformative bases decreases and the 5′ sequence diversity increases, which is beneficial for sequencing clustering. Based on these observations, we decided to include R2-UMI reads in the analysis.

Examination of both FS-UMI and 10x data revealed the expected cell types (Supplementary Fig. 18a–d)^19^. In general, the ability of 10x to reconstruct cell fate progression was facilitated by the larger number of cells analyzed, whereas FS-UMI benefited from a higher information content per cell, simplifying the annotation of amacrine/horizontal progenitors (*PTF1A*^+^) or OFF-center-bipolar cells (*GRIK1*^+^) (Supplementary Fig. 18a-b). The choice of FS-UMI has three advantages over 10x. First, the number of genes detected and the gene diversity per cell type is higher (Fig. 2f,g), even after downsampling FS-UMI data to the average 10x raw reads (Supplementary Fig. 18e). This translates into a detection of 4.1 ± 2.9 times more cell-type marker genes (UMI + internal reads; Supplementary Fig. 19a).

We found a 79.7 ± 19.6% (mean ± s.d.) overlap between cell-type markers identified with FS UMI reads (mean ± s.d._DE_markers_ = 539 ± 329) and FS internal reads (mean ± s.d._DE_markers_ = 742 ± 509). The log_2_-fold changes in these shared markers were highly correlated, with an almost systematically higher value for internal-read markers (Supplementary Fig. 19b). We observed a similar relationship between UMI reads and internal-reads gene counts (Supplementary Fig. 19c).

Second, FS full gene-body coverage can better inform about isoforms. The percentage of FS reads confidently assigned to the transcriptome was 72.5 ± 11.1% (mean ± s.d., UMI + internal reads) compared to 48.5% with 10x Genomics. Moreover, 44 ± 14.3% of the cell-type markers harbored two or more differentially expressed isoforms, some with a known impact on protein function. For instance, we observed two isoforms of pyruvate kinase (*PKM*), a key enzyme in photoreceptor glucose metabolism, characterized by the mutually exclusive presence of exon 9 (PKM1) or 10 (PKM2) (Supplementary Fig. 20a,b)^20^. PKM1 favors oxidative phosphorylation, whereas PKM2 is associated with anabolic processes and anaerobic glycolysis^20,21^. In line with previous in situ hybridization experiments in mice, amacrine/horizontal/bipolar cells from retinal organoids mainly expressed exon 9 (PKM1), whereas rods also expressed exon 10 (PKM2) (Supplementary Fig. 20a,b)^21^. PKM isoforms could not be told apart from 10x Genomics data (Supplementary Fig. 20c).

Third, the full-length information enabled the analysis of genomic SNPs at the single-cell level. We called SNPs on individual cells processed with FS-UMI (*n*_SNPs_DP>2_ = 4,210,503) and compared the results to exome sequencing (*n*_SNPs_passing_filters_ = 34,078) of the same retinal organoids (Fig. 2h). For each cell, the comparison was restricted to exome-sequencing SNPs supported by more than 2 reads in FS-UMI. At 750 K raw reads, 1,104 ± 182 (mean ± s.d.) true SNPs were detected per cell, which represents 61.57 ± 1.7% (mean ± s.d.) of the identified exome-sequencing SNPs in transcribed regions . We also observed, on average, 1,831 ± 369 false-positive calls (mean ± s.d.). Both true- and false-positive SNPs scaled with depth and were highly correlated (Supplementary Fig. 20d,e). In comparison, aggregated reads from 10 or 100 rods processed with 10x Genomics led to the detection of 85.8 ± 20.7 (mean ± s.d._mapped_reads_ = 269 K ± 67 K) and 640 ± 24 (mean ± s.d._mapped_reads_ = 2,536 K ± 111 K) true-positive SNPs, respectively, whereas the levels of false-positive calls in each case were similar (Supplementary Fig. 20f).

These results show that a substantial number of SNPs can be captured with FS and highlight the need to use a reference or/and validate the discovered variants whenever possible.

Here, we have presented FS, a full-length scRNA-seq method that, thanks to a unique combination of additives and enzymes, is faster and more sensitive than any other protocol published so far. It perfectly complements droplet methods while providing higher sensitivity and information about isoform usage and SNPs. The cost per cell is lower than other commercial and noncommercial methods, and comparable to SS3 (<$1; Supplementary Tables 2–4).

While this article was in preparation, a new method called Smart-seq3xpress was uploaded on *bioRxiv* (ref. ^22^). Although FS-LA and Smart-seq3xpress share several similarities, we are of the opinion that the use of oil to prevent reagent evaporation, the minute reaction volume, the longer RT reaction and the separation of RT and PCR make Smart-seq3xpress more complex and less user-friendly than FS.

We are confident that FS will be adopted by researchers looking for an efficient, robust, affordable and automation-friendly protocol.

## Methods

### Preparation and FACS of hPBMCs

Leukocytes were isolated from whole blood by density gradient centrifugation using Ficoll-Paque (Sigma-Aldrich). If not used immediately, cells were cryopreserved in fetal bovine serum (FBS) + 10% DMSO (Sigma-Aldrich). Prior to fluorescence-activated cell sorting (FACS), cells were thawed, resuspended in 1x PBS + 2% FBS and stained with an fluorescein isothiocyanate-conjugated mouse anti-human CD45 antibody at a final dilution of 1:50 (clone HI30, BD Biosciences, 560976) for 25 min on ice. Unbounded antibodies were washed away by adding 3 ml of 1× RPMI 1640 medium (ThermoFisher Scientific) + 2% FBS, centrifuged for 5 min at 300×*g*, resuspended in 1× PBS + 0.04% BSA, strained through a 40-µm filter (pluriSelect) and stained with propidium iodide (PI) (1 mg ml^−1^, ThermoFisher Scientific) at room temperature to label dying cells.

Fluorescein isothiocyanate^+^ PI^−^ single cells were sorted on a FACSAria Fusion (100-µm nozzle, 20 psi) instrument equipped with FACSDiva software (v8.0.2, BD Biosciences) (Supplementary Fig. 21 shows the gating strategy). Cells were sorted in different volumes of lysis buffer according to plate type (5 µl when using 96-well plates, 1 or 0.5 µl when using 384-well plates). LoBind twin.tec plates (Eppendorf) were used in all experiments.

### Preparation and FACS of HEK293T cells

HEK293T cells (CRL-3216, ATCC) were cultured in DMEM (ThermoFisher Scientific) supplemented with 10% FBS, 2 mM l-glutamine (Ambion) and 1% penicillin/streptavidin (Gibco). Cells were cryopreserved in FBS + 10% dimethylsulfoxide. Upon thawing, 1 ml prewarmed DMEM was added to the vial, the contents were transferred to a 15-ml Falcon tube containing 10 ml prewarmed DMEM and the cells were centrifuged for 5 min at 300×*g*. The supernatant was removed, and the cells were resuspended in 1× PBS + 0.04% BSA, strained through a 40-µm filter and stained with PI at room temperature to label dying cells. PI^−^ single cells were sorted on a FACSAria Fusion instrument (100-µm nozzle, 20 psi).

### Retinal organoids

Retinal organoids were generated from the induced pluripotent stem cell line 01F49i-N-B7 at passage 39, as described in Cowan et al.^19^. At week 18, retinal organoids were selected based on the presence of outer segments and characteristic retinal layers. Retinal and nonretinal parts of organoids were dissected and maintained in 3:1 medium, supplemented with N_2_ at 37 °C until dissociation. Dissected organoids were pooled together and washed once with 1 ml Ringer solution without calcium. The Neural Tissue Dissociation Kit P (Miltenyi Biotec) was used to dissociate organoids into single cells, following the manufacturer’s instructions. The resulting cell suspension was spun for 5 min at 300×*g* in a prerefrigerated centrifuge (Eppendorf). The supernatant was carefully removed without disturbing the pellet, and samples were processed as described above for HEK293T cells. Only debris, doublets and PI^+^ cells were excluded from sorting.

### scRNA-seq of retinal organoids on the 10x Genomics platform

The cellular suspension was used to load 10 K cells on each of two lanes of a Chromium instrument (10x Genomics). scRNA-seq libraries were prepared using the Next GEM Single Cell 3′ Gel Bead and Library v3.1 kit (CG000204 Rev C) and one of the two libraries was sequenced on an Illumina NovaSeq6000 (28-8-0-91).

### Lysis buffer preparation for FS and FS-LA

Lysis buffer was dispensed in single wells of a 384-well plate with the I.DOT (Dispendix), and the plate was sealed and stored at −20°C until needed. Lysis buffer was composed of 0.02 µl Triton-X100 (10% v/v, Sigma-Aldrich), 0.24 µl dNTP mix (25 mM each, Roth), 0.018 µl FS-dT_30_VN (5′Bio-AAGCAGTGGTATCAACGCAGAGTACT_30_VN-3′ (Bio, biotin); 100 µM, IDT), 0.03 µl RNAse inhibitor (40 U/µl, Takara), 0.012 µl dithiothreitol (100 mM, ThermoFisher Scientific), 0.2 µl betaine (5 M, Sigma-Aldrich), 0.09 µl dCTP (100 mM, ThermoFisher Scientific), 0.092 µl FS-TSO (5′Bio-AAGCAGTGGTATCAACGCAGAGTACrGrGrG-3′ (Bio, biotin); 100 µM, IDT), and water to 1 µl final volume. After sorting, plates were sealed with aluminum foil seals (VWR) and immediately placed in a −80 °C freezer until ready. Of note, FS dT_30_VN and SMART dT_30_VN have the same sequence and are used interchangeably in the figures and tables. Moreover, dCTP and FS-TSO can be removed from the lysis buffer mix and dispensed with the RT-PCR mix instead, without negatively affecting the overall results.

### Lysis buffer preparation for FS-UMI

Lysis buffer composition: 0.02 µl Triton-X100 (10% v/v), 0.24 µl dNTP mix (25 mM each), 0.018 µl STRT-dT_31_ oligonucleotide (5′Bio-AATGATACGGCGACCACCGATCGT_31_-3′ (Bio, biotin); 100 µM, IDT), 0.03 µl RNAse inhibitor (40 U/µl), 0.012 µl dithiothreitol (100 mM), 0.2 µl betaine (5 M), 0.09 µl dCTP (100 mM), and water to 1 µl final volume.

### RT-PCR reaction for FS and FS-LA

Plates were removed from the −80 °C storage, transferred to a preheated Mastercycler thermocycler (Eppendorf), incubated for 3 min at 72 °C and then placed on a metal block kept in an ice bucket. Four microliters of RT-PCR mix were added using the I.DOT. RT-PCR mix composition: 0.238 µl dithiothreitol (100 mM), 0.8 µl betaine (5 M), 0.046 µl magnesium chloride (1 M, Ambion), 0.096 µl RNAse inhibitor (40 U µl^−1^), 0.05 µl Superscript IV (200 U µl^−1^, ThermoFisher Scientific), 2.5 µl KAPA HiFi Hot-Start ReadyMix (2×, Roche) and nuclease-free water to 4 µl final volume.

The following RT-PCR program was used: 60 min at 50 °C, 98 °C for 3 min, then *N* cycles of (98 °C for 20 s, 67 °C for 20 s, 72 °C for 6 min). The number of cycles depended on the cell type and protocol (guidelines in Supplementary Note 1). For FS, we chose *N* = 19 for HEK293T cells and *N* = 21 for hPBMCs. For FS-LA, we used *N* = 4, *N* = 6, *N* = 8, *N* = 10, *N* = 12 and *N* = 16.

### RT-PCR reaction for FS-UMI

The protocol is the same as that described above for FS/FS-LA, with the following minor differences in the RT-PCR mix composition: 0.238 µl dithiothreitol (100 mM), 0.8 µl betaine (5 M), 0.046 µl magnesium chloride (1 M), 0.096 µl RNAse inhibitor (40 U µl^−1^), 0.05 µl Superscript IV (200 U µl^−1^), 2.5 µl KAPA HiFi Hot-Start ReadyMix (2×), 0.092 µl TSO carrying UMIs and a 5-bp spacer (TSO-UMI, 5′Bio-AAGCAGTGGTATCAACGCAGAGTNNNNNNNNXXXXXrGrGrG-3′ (NNNNNNNN = UMI; XXXXX = 5-bp spacer); 100 µM, IDT), 0.05 µl Tn5-ISPCR forward primer (5′-TCGTCGGCAGCGTCAGATGTGTATAAGAGACAGAAGCAGTGGTATCAACGCAGAGT-3′, 100 µM, IDT), 0.01 µl DI-PCR-P1A reverse primer (5′-AATGATACGGCGACCACCGA-3′, 100 µM, IDT) and nuclease-free water to 4 µl final volume. The following RT-PCR program was used: 60 min at 50 °C, 98 °C for 3 min, then *N* cycles of (98 °C for 20 s, 65 °C for 20 s, 72 °C for 6 min). We used *N* = 21 for HEK293T cells, *N* = 23 for hPBMCs and *N* = 24 for week 18 organoids. A complete list of the TSOs as well as all oligonucleotides used in this paper is given in Supplementary Note 2.

### Sample cleanup (all protocols except FS-LA)

Samples were cleaned up using SeraMag SpeadBeads (GE Healthcare) containing 18% w/v polyethylene glycol (molecular weight = 8,000) (Sigma-Aldrich) on a Fluent 780 workstation (Tecan). A detailed protocol is described elsewhere^23^. A 0.8:1 ratio of beads to cDNA was used for most applications. In SS3 and FS-UMI, we used a 0.6:1 ratio. In FS-LA, no cleanup was performed, and 1 µl cDNA was used directly for tagmentation after RT-PCR.

### Sample QC (all protocols except FS-LA)

Sample cDNA concentration was measured using the Quant-iT PicoGreen dsDNA assay kit and black Nunc F96 MicroWell polystyrene plates (ThermoFisher Scientific) according to the manufacturer’s instructions, except that we used half of the recommended reaction volumes to reduce costs. Fluorescence measurements were recorded on a Hidex Sense instrument (Hidex). To assess cDNA size distribution, eleven samples were randomly selected from each plate, loaded on a HS DNA chip and run on a 2100 Bioanalyzer System (software vB.02.10.51764, Agilent).

### Sample normalization (all protocols except FS-LA)

Samples were diluted to a final cDNA concentration of 100–200 pg µl^−1^ based on the PicoGreen measurements. This plate represented the working dilution used later for library preparation.

### Standard tagmentation and enrichment PCR

Tagmentation was carried out using different kits.

- Library preparation with the Nextera XT kit (Illumina) was done in different ways according to the following protocol.
  For standard and miniaturized FS and FS method development, 1 μl cDNA working dilution (100-200 pg µl^−1^) was transferred to a new plate before adding 0.5 µl ATM (Amplicon Tagment Mix), 2 µl TD (Tagment DNA buffer), and nuclease-free water to 4 µl final volume. The plate content was incubated for 8 min at 55 °C, and the Tn5 was inactivated with 1 µl NT (Neutralize Tagment) buffer, followed by a 5-min incubation at room temperature, before adding 3 µl NPM (Nextera PCR Master mix) and 2 µl premixed custom N7xx and S5xx index adapters (5 µM each). The enrichment PCR reaction was done at 72 °C for 3 min, 95 °C for 30 s and then 14 cycles of (95 °C for 10 s, 55 °C for 30 s, 72 °C for 30 s), 72 °C for 5 min, 4 °C hold. The sequences of all index adapters used in this study are available on protocols.io (https://www.protocols.io/view/flash-seq-protocol-b6myrc7w/materials).
  For FS-UMI libraries (HEK293T, hPBMC), we used 1 µl normalized cDNA (100-200 pg µl^−1^), 1 µl TD buffer, and 0.08–0.2 µl ATM. For retinal organoids, 1 µl cDNA working dilution (100 pg µl^−1^) was transferred to a new plate before adding 0.2 µl ATM and 1 µl TD. Samples were incubated for 8 min at 55 °C. After inactivation with 0.5 µl 0.2% SDS for 5 min at room temperature, 1.5 µl NPM and 1 µl pre-mixed custom N7xx and S5xx index adapters (5 µM each) were added to the reaction. The enrichment PCR reaction was carried out as described above. Input cDNA and amount of ATM were adjusted to obtain sequencing-ready libraries 700–1,000 bp long.
- Library preparation using homemade Tn5 transposase (FS method development and FS-LA). Tn5 transposase was produced by the EPFL Protein Facility (Lausanne, Switzerland). A modified version of the original protocol was used for protein preparation^13,24^. The tagmentation reaction mix contained 1 µl cDNA (150 pg µl^−1^), 0.8 µl 100% dimethylformamide (Sigma-Aldrich), 0.8 µl 5× TAPS buffer (50 mM TAPS-NaOH pH 7.3 at 25 °C, 25 mM MgCl_2_), 0.0125 µl Tn5 transposase (~2 µM) and nuclease-free water to 4 µl final volume. The plate was incubated for 8 min at 55 °C, and the Tn5 was inactivated with 1 µl 0.2% SDS for 5 min at room temperature. Enrichment PCR mix contained 0.2 µl KAPA HiFi DNA polymerase (1 U µl^−1^), 0.3 µl dNTP mix (10 mM), 2 µl KAPA HiFi Buffer (5×) (all part of the KAPA HiFi PCR kit, Roche), 2 µl premixed custom N7xx and S5xx index adapters (5 µM each) and water to 5 µl final volume. The enrichment PCR reaction was carried out as described above.
- plexWell Rapid single-cell kit (seqWell) (FS method development). Purified cDNA was diluted to a final concentration of 250 pg µl^−1^; 4 µl cDNA was used to generate sequencing libraries, following the manufacturer’s instructions.

### Tagmentation and enrichment PCR of FS-LA samples

In FS-LA, several aspects of the workflow had to be modified according to the number of preamplification PCR cycles used. Here, we provide some general guidelines but appropriate reaction conditions will require further adjustments, depending on the cell type used (Supplementary Note 1).

RT-PCR was performed in 384-well plates in a final volume of 5 µl. We used 1 µl unpurified amplified cDNA directly for tagmentation. In pilot experiments, we observed that leftover primers, dNTPs and salt could completely inhibit the activity of the Tn5 transposase. An acceptable compromise was to dilute the cDNA ten times (i.e., adding 1 µl cDNA and 9 µl tagmentation mix with homemade Tn5 or using 1 µl 1:10 cDNA diluted in nuclease-free water).

### Sample pooling and sequencing (all protocols)

After enrichment PCR cells were pooled together and cleaned up with a 0.8:1 SeraMag SpeadBeads containing 18% w/v polyethylene glycol (molecular weight = 8,000). Libraries were sequenced using a NextSeq 550 (control software v4.0.1) high-output kit v2.5 (75 cycles) with read mode 75-8-8-0. FS-UMI libraries were sequenced using a NextSeq 550 high (75 or 150 cycles) or mid (150 cycles) output kit v2.5 with read mode 75-8-8-0 or 100-8-8-50, respectively. One of the FS-UMI retinal organoid libraries (plate 316) was resequenced using a NextSeq550 high-output kit v2.5 (75 cycles) to increase the number of reads per cell.

### Other single-cell protocols

Smart-seq2 libraries were generated according to Picelli et al.^25^. Smart-seq3 libraries were prepared according to the protocols.io guidelines (10.17504/protocols.io.bcq4ivyw). SMART-seq single-cell kit (Takara Bio) libraries were prepared following the manufacturer’s instructions, with the exception that all reaction volumes were decreased by half.

### Statistical analysis

Cell-to-cell correlations were calculated using Kendall’s tau rank correlation to handle ties (pcaPP, v1.9-74). Only genes expressed in all the compared groups were used to calculate correlations. Multiple comparisons were performed using Dunn’s test or Wilcoxon rank-sum test (rstatix, v0.7.0).

Box plots in this paper are defined as follows: central part, median; lower/upper hinges, 25th/75th percentile; whiskers, 1.5× interquartile ranges; and points, outliers.

### Full-length scRNA-seq data preprocessing

Sequencing adapter leftovers were trimmed using BBDUK (BBMAP, v38.86) in all reads. When required, Umi-tools (v1.1.1) was used to extract UMIs from 5′ UMI reads and trim spacer sequences, FS/SS3 adapters and GGG motifs originating from the TSO. Non-UMI reads >60 bp or UMI reads >30 bp were aligned against hg38 (Gencode v34) using STAR (–limitSjdbInsertNsj 2000000 –outFilterIntronMotifs RemoveNoncanonicalUnannotated, v2.7.3) in single-pass mode. Primary alignments of mapped reads were retained with samtools (v1.10). Non-UMI reads were assigned to a feature from Gencode v34 annotation (primary_assembly) with featureCounts (-t exon -g gene_name –fracOverlap 0.25, v2.0.0). UMI reads were treated as stranded, assigned to a feature using featureCounts (-s 1 -t exon -g gene_name –fracOverlap 0.25, v2.0.0), and deduplicated/counted using umi_tools counts (–per-gene, v1.1.1). Gene-body coverages, genome-wide read distributions and read GC contents were estimated using ReSQC (v4.0.0). Isoforms were counted using Salmon (v1.5.2) on a prebuild hg38 (salmon_sa_index) index with decoy genome using the following parameters: –minAssignedFrags 1–dumpEqWeights –validateMappings. Library-specific parameters were adapted to the read type (internal: U, UMI: SF).

When working on paired-end data, the following modifications were made to the pipeline. In FS-UMI, UMI sequences were extracted from read 1 or read 2 (Methods, 'UMI in read 2 reads'). UMI reads were mapped with STAR (–seedSearchStartLmax 30 –limitSjdbInsertNsj 2000000 –outFilterIntronMotifs RemoveNoncanonicalUnannotated). UMI reads invading the adjacent sequence were discarded using a custom R script (filterInvasionEventsfromBAM.R, max.mismatch = 1). UMI reads were deduplicated/counted using umi_tools counts (–per-gene –chimeric-pairs = discard –unpaired-reads = discard, v1.1.1).

All down-samplings were performed on the FASTQ files using seqkt (-s42, v1.3-r106).

### Full-length scRNA-seq postprocessing

The resulting data were parsed using R (v4.1.0). Downstream data exploration of the gene counts was performed using Seurat^26^ (v4.0.3). Generally, only cells with >50 K or >100 K raw reads, >60% uniquely mapped reads and <25% or <20% unmapped reads were selected. Mitochondrial, ribosomal and MALAT1 genes/isoforms were excluded from the analysis. SCTransform (v0.3.2) was used to normalize the count matrix. The sample’s read counts and, in hPBMCs, the percentage of mitochondria reads, were regressed out. The most variable genes were selected using variance stabilizing transformation. Nonlinear dimension reduction was performed using UMAP (uwot, v0.1.10) on the principal-component analysis values. Distinct clusters and individual cell communities were identified using Seurat FindNeighbors and FindClusters functions. Differentially expressed cell-type markers were identified using FindAllMarkers (test.use = ”MAST”, logfc.threshold = 0.5, pos.only = T). hPBMCs cell types were automatically assigned with Azimuth^26^. In retinal organoids, cell types were assigned based on the top cell markers and markers obtained from Cowan et al.^19^. TCR rearrangements were extracted from hPBMC data using TRUST4 (ref. ^27^) (v1.0.6). TCR sequences with CDR3aa sequence less than eight amino acids, out-of-frame CDR3aa or missing V-J-C chain were excluded.

Isoform pseudocounts generated by Salmon were imported into R using tximport (v1.20.0). For retinal organoids, length scaled transcripts per million were processed as described above with Seurat v4.0.3, except for SCTransform, which was replaced with NormalizeData. Differentially expressed cell-type markers were identified using FindAllMarkers (test.use = “t-test”, logfc.threshold = 0.5 pos.only = T, min.pct = 0.25).

### Strand invasion

The 20-bp sequence adjacent to the read start of unambiguously assigned UMI reads was extracted using *getSeq* (BSgenome v1.60.0, BSgenome.Hsapiens.UCSC.hg38, v1.4.3) taking into account the read orientation. To avoid overcounting PCR duplicates, UMI reads harboring the same UMI sequence, mapping position and adjacent read-start sequence were collapsed (= deduplicated 5′ UMI reads). The adjacent sequence was compared to the UMI looking for a perfect or partial match (= consecutive 5′ mismatches) in the presence or absence of an additional ‘GGG’ or ‘SPACER-GGG’ motif.

To differentiate the reads mapping to exons or introns, a GTF file regrouping the collapsed exonic and intronic genomic sequences of all genes was created using custom R scripts. This GTF file was used to assign mapped 5′ UMI reads to exonic or intronic features in an unstranded manner with featurecounts (-s 0 –largestOverlap –fracOverlap 0.25 -R BAM). The annotated BAM files were parsed using custom R scripts to retrieve the read position, mapping strand, UMI sequence and gene annotation.

Seqlogo graphics were produced using ggseqlogo (v0.1).

### UMIs in read 2 reads

UMI reads were identified using umi_tools extract (v1.1.1). The motif searched was adapted to the method and read type. UMI reads in FS-UMI read 1 (R1-UMI) were defined as starting with any full or partial 5′ adapter sequence (= full-length sequence − *N* 5′ nucleotides, where *N* = 1, …, [full-length-2]), followed by eight random nucleotides (= UMI), spacer sequence, and ‘GG’. UMIs in read 2 (R2-UMI) from FS-UMI were identified using a motif made of two to four bases of the 5′ adapter (‘^GAGT | ^AGT | ^GT’) followed by eight random nucleotides, spacer sequence and GG.

SS3 data were obtained from Hagemann-Jensen et al. (E-MTAB-8735, ‘Mouse fibroblast - Gel cut’). A total of 150 cells were randomly selected. In SS3, R1-UMI were defined as starting with any full or partial 5′ adapter sequence (= full-length sequence − *N* 5′ nucleotides, where *N* = 1, …, [full-length-4]), followed by eight random nucleotides (= UMI) and GG. SS3 R2-UMIs were defined as starting with a partial 5′ adapter, similarly to SS3 R1-UMIs.

To account for the difference in R1-UMI/R2-UMI orientations, read 2 of R2-UMIs were used as read 1. Conversely, read 1 of R2-UMIs was used as read 2. Reads were cropped to a maximum of 75 bp using trimmomatic (v0.39). Only paired reads of >25 bp were retained. The remaining reads were mapped and assigned to a feature and the gene counts were quantified as described above, except for *–*seedSearchStartLmax, which was set to 25 for the mapping. Gene-body coverages were assessed using ReSQC on reads trimmed to a common 30 × 30-bp length to account for the differences in read 1 versus read 2 lengths.

### 10x Genomics data

Data were processed with CellRanger (v6.1.1). Ambient RNA was removed using SoupX (v1.5.2). Single cells were filtered based on the following criteria: nCount_RNA [1500–25000], nFeature_RNA [< 250], percent.mito [0.25–12.5]. Doublets were removed using DoubletFinder (v2.0.3). The filtered count matrix was processed with Seurat SCT transform, regressing out the percentage of mitochondrial reads (variable.features.n = 3,000, v4.0.3). UMAP was built on the top 20 first PCA values. Cell markers were identified using FindAllMarkers (test.use = “MAST”, logfc.threshold = 0.5, only.pos = T). Cell types were assigned based on markers from Cowan et al.^19^. A subgroup of cells of unknown origin associated with few detected molecules (*n*Count_RNA < 2,000) and mainly expressing lncRNA (NEAT1, MALAT1, …) and apoptotic markers (e.g., BBC3) were defined as apoptotic cells.

### Variant calling

DNA for the exome sequencing was extracted from the central nonretinal part of retinal organoids using the Qiagen AllPrep Dna/Rna Mini kit (Qiagen). The sequencing library was generated with Twist Bioscience Human Core Exome Plus capture kit by CeGaT and sequenced on an Illumina NovaSeq6000 instrument. Raw sequencing files were aligned to the GRCh38 reference using bwa (v0.7.17-r1188). Duplicates were marked using Picard (v2.23.8-2-ga004f14-SNAPSHOT). Variants were called by HaplotypeCaller including base quality score recalibration (GATK v4.1.4.1) according to GATK best practices and were selected to be present in the target regions of the capture kit (that is exons). Variants passing filters were defined as QD > 2, FS < 60, SOR < 3, MQ > 40, MQRankSum > −12.5 and ReadPosRankSum > −8. QD, variant confidence/quality by depth; FS, phred-scaled *P*-value using Fisher's exact test to detect strand bias; SOR, symmetric odds ratio of 2x2 contingency table to detect strand bias; MQ, root mean square mapping quality.

Variants from the scRNA data were called separately in each cell. Reads were aligned using STAR (v2.7.3) in a two-pass mapping mode. New splice junctions discovered during the first mapping pass of all analyzed cells were filtered to retain canonical junctions supported by more than two non multimapped reads in at least one cell. Mitochondrial splice junctions were excluded. Unmapped and secondary alignments were filtered out using samtools (v1.10). The resulting BAM files were processed following GATK RNA-sequencing best practices with GATK (v4.2.2.0) and PicardTools (v2.26.5). Variants were called using bcftools mpileup (v1.10.2, –skip-indels -q 15) followed by bcftools call (-c -v). FASTQ files were downsampled using seqtk (-s42, v1.3-r106). VCF files were parsed using R (v4.1), bamSignals (v1.24.0) and vcfR (v1.12.0).

For each cell, the list of putative true SNPs was defined as the exome-sequencing SNPs expressed with more than 2 reads (=reference list). FS-UMI SNPs outside of the exome-sequencing coverage were filtered out. True-positive SNPs were defined as FS SNPs with DP > 2 and detected in the reference list. The number of false-negative variants was defined as the difference between the number of SNPs in the reference list and the number of true-positive SNPs. False-positive SNPs were defined as detected in FS, but not in the reference list.

### Reporting summary

Further information on research design is available in the Nature Research Reporting Summary linked to this article.

## Online content

Any methods, additional references, Nature Research reporting summaries, source data, extended data, supplementary information, acknowledgements, peer review information; details of author contributions and competing interests; and statements of data and code availability are available at 10.1038/s41587-022-01312-3.

## Supplementary information

Supplementary Information Supplementary Figures 1–21, Discussion and Notes 1 and 2.

Reporting Summary

Supplementary Table 1 Protocol timing and sample cost calculation.

## Data Availability

Sequencing data related to HEK293T cells and hPBMCs have been deposited to Sequence Read Archive (PRJNA816486). Count tables, other processed data and cDNA yields associated with this study are available on Mendeley data (10.17632/bh47n6fnpd.1). FS protocols have been uploaded on protocols.io (https://www.protocols.io/view/flash-seq-protocol-kxygxzkrwv8j/v3, https://www.protocols.io/view/flash-seq-low-amplification-protocol-yxmvmnod5g3p/v3 and https://www.protocols.io/view/flash-seq-umi-protocol-bp2l619rdvqe/v3)

## Code availability

Scripts used to process the data are available at: https://github.com/vincenthahaut/FLASH-seq.
