# Supplementary material for: Fast and highly sensitive full-length single-cell RNA sequencing using FLASH-seq
Source: Nat Biotechnol. 2022 May 30;40(10):1447–51. doi: 10.1038/s41587-022-01312-3 (PMC9546769; doi:10.1038/s41587-022-01312-3)
Supplement: Supplementary file 1 — Supplementary Figures 1–21, Discussion and Notes 1 and 2. [file 41587_2022_1312_MOESM1_ESM.pdf]

nature portfolio

https://doi.org/10.1038/s41587-022-01312-3

Supplementary information

Fast and highly sensitive full-length single-cell RNA sequencing using FLASH-seq

In the format provided by the authors and unedited

Supplementary Figures

Supplementary Fig. 1

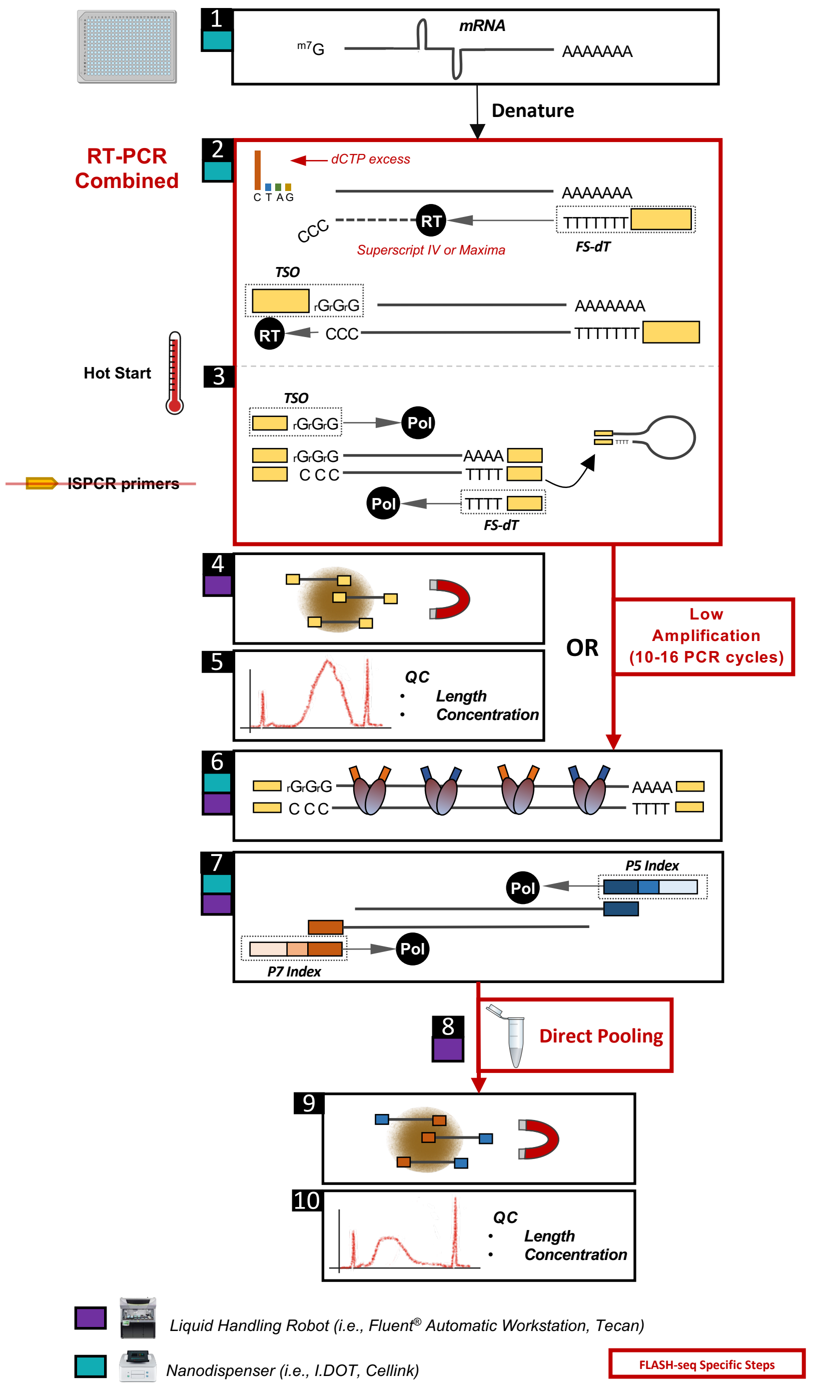

Supplementary Fig. 1

FLASH-seq workflow.

Steps automated with a liquid handling robot or nanodispenser are marked by a purple and teal square, respectively. Steps/reagents differing from Smart-seq2 are highlighted in red. **Step 1**, cells are individually sorted in plates containing a lysis buffer. **Step 2**, after denaturation, mRNAs are reverse transcribed using a template-switching reverse transcriptase (RT) in the presence of an excess of dCTP. **Step 3**, cDNA is amplified by semi-suppressive PCR. Only a single master mix is required to perform Step 2 and 3. **Step 4,** cDNA is purified using magnetic beads. **Step 5**, cDNA concentrations and fragment sizes are measured and the samples are diluted to a final concentration of ~100-200 pg/µl. **Step 6**, cDNA is tagmented using a Tn5 transposase which introduces known adaptor sequences at both ends of each fragment. **Step 7**, tagmented cDNA is amplified by PCR and barcoded sequencing adaptors are added. **Step 8**, samples are pooled. **Step 9,** the library is purified using magnetic beads. **Step 10**, concentrations and average fragment sizes are measured in preparation for the sequencing. Steps 4 and 5 are not performed in FS low amplification.

Supplementary Fig. 2

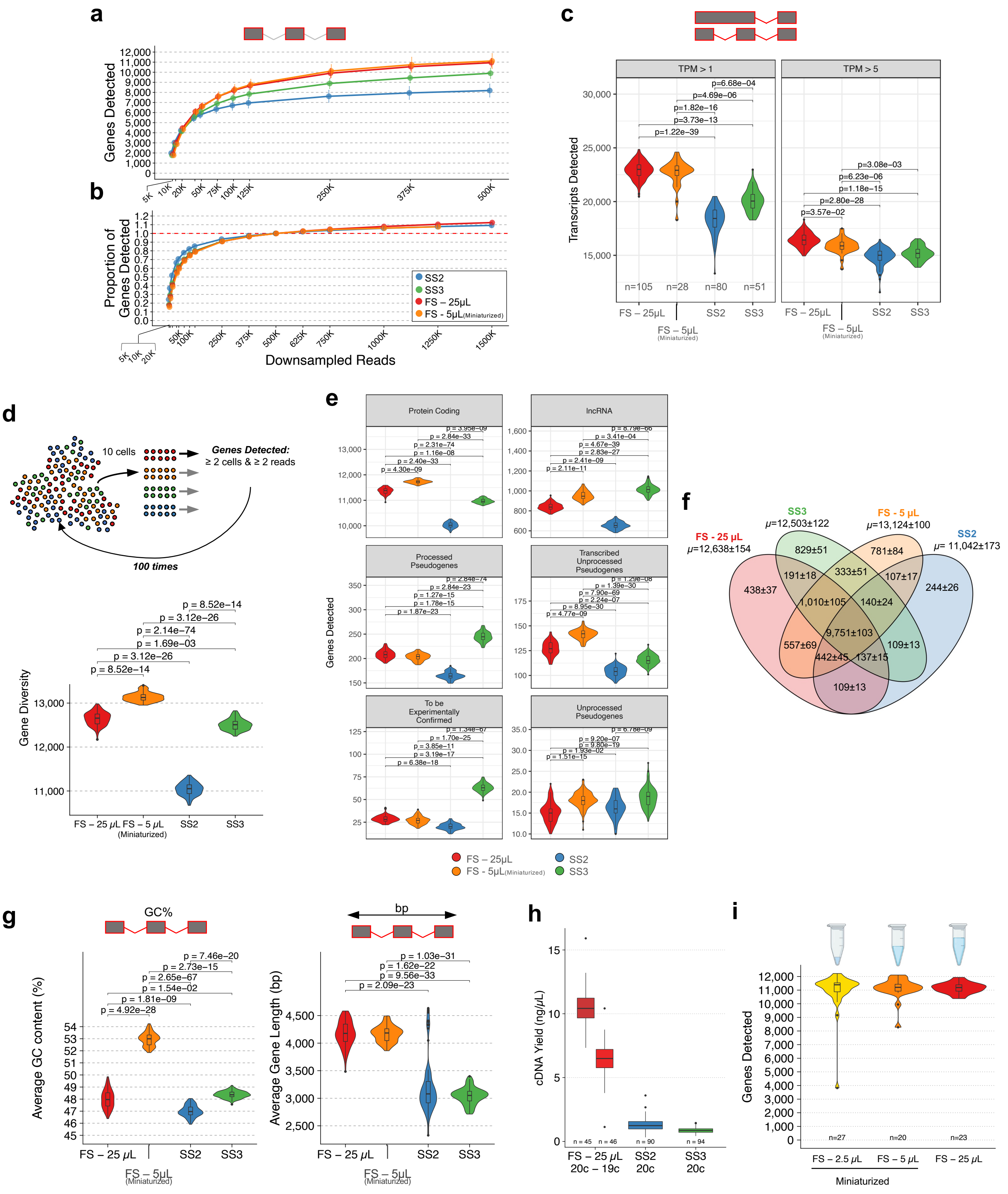

Supplementary Fig. 2

Analysis of FLASH-seq gene detection in HEK293T cells.

a. Mean (± s.d.) genes detected (>0 reads) in HEK293T cells processed with SS2 (n = 80), SS3 (n = 51), FS - 25 µl (n = 105) or FS-5µl$_{miniaturized}$ (n = 28) using 5K to 500K downsampled raw reads.

b. Mean (± s.d.) proportion of genes detected at various sequencing depths compared to the cell reference number of genes detected at 500K raw downsampled reads (red line). In both (a) and (b), some conditions did not have sufficient coverage to be represented at each point. At >1M reads: SS3 (n = 0), FS - 5 µl (n = 2), SS2 (n = 73), FS - 25 µl (n = 77).

c. Number of isoforms detected in the same cells as (a), downsampled to 500K raw reads, with a library scaled transcript-per-million count (TPM) >1 or >5.

d. Resampling strategy used to evaluate the gene diversity and account for the differences in cell numbers. Ten cells with 500K downsampled reads were randomly picked in each group and the number of genes expressed in ≥2 cells with ≥2 reads was obtained (= gene diversity). The resampling was performed 100 times.

e. Gene diversity subdivided by gene type.

f. Mean (± s.d.) number of genes overlapping between methods when performing the cell resampling.

g. Average GC content (%, left) and gene length (bp, right) of the unique genes obtained in (d).

h. cDNA yield, in ng/µl for SS2 (20 PCR cycles), SS3 (20) or FS - 25 µl (20 or 19).

i. Impact of the reaction volume on the number of detected genes in HEK293T cells (500K downsampled reads). Cells were simultaneously processed in a 384-well plate in a final reaction volume of 25 µl (n = 23), 5 µl (n = 20) or 2.5 µl (n = 27). No statistically significant differences were found (P > 0.05).

Multiple comparisons in (d, e, g, h) were performed using a two-sided Dunn's test, Bonferroni correction, adj. P-value < 0.05 displayed.

Supplementary Fig. 3

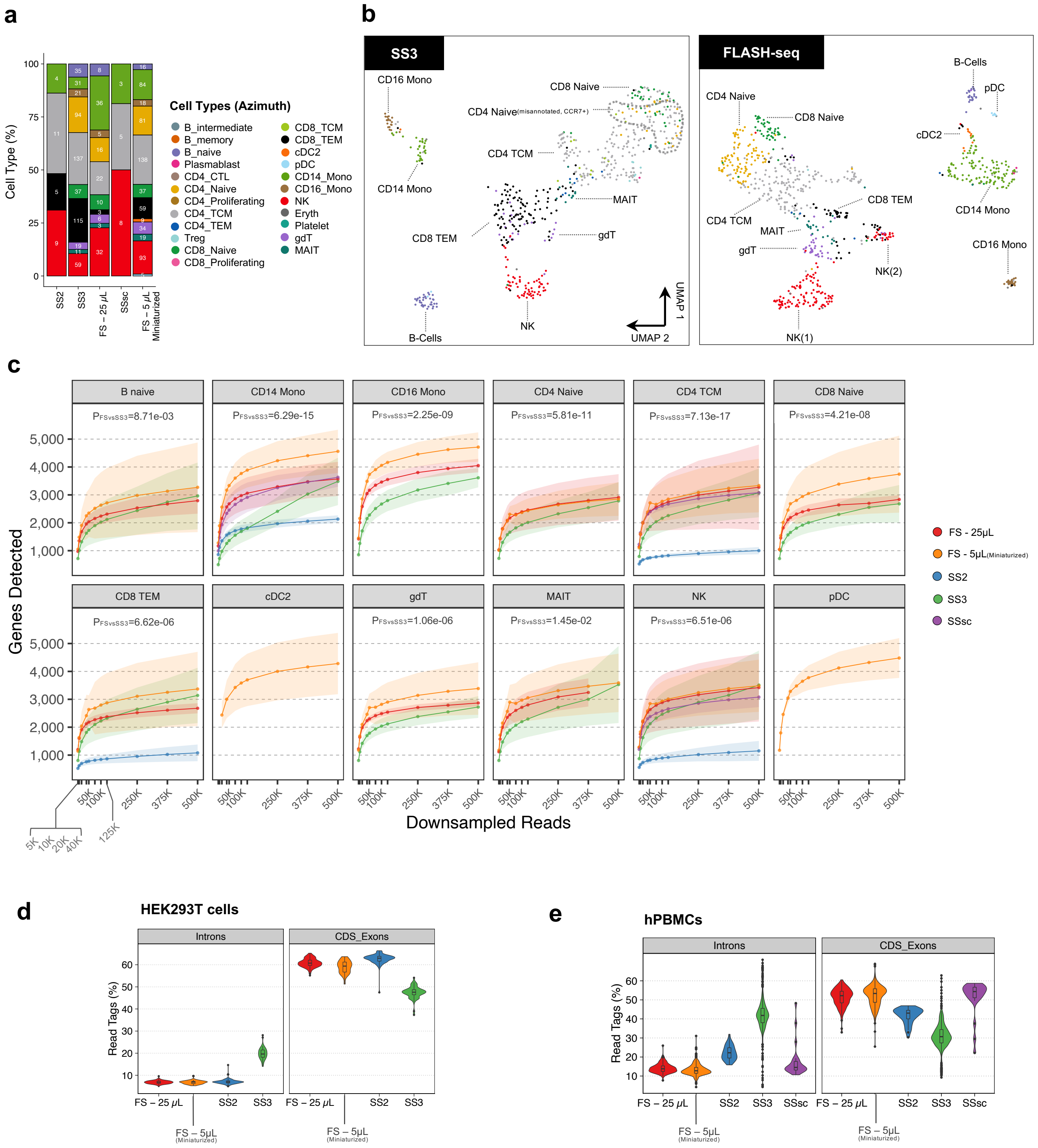

Supplementary Fig. 3

Analysis of FLASH-seq gene detection in hPBMCs.

a. Cell type distribution per protocol, in percentage of cells. The number of cells per cell type is depicted in white.

b. UMAP of hPBMCs at 125K raw reads processed with SS3 (left panel, *n* = 593) and FLASH-seq (right panel, $n_{FS\text{-}25\,\mu l}$ = 147, $n_{FS\text{-}5\mu l}$ = 608), colored by cell-types (Azimuth, predicted.celltype.l2). In SS3, CD4 naïve (CCR7^{+}) were misannotated by the automatic pipeline.

c. Mean (± s.d.) number of detected genes (> 0 reads) per hPBMCs cell type processed with either SS2, SS3, SMART-Seq Single Cell kit (Takara, SSsc) or FS (in 25 µl or 5 µl) using 5K to 500K downsampled raw reads. Some cells did not have sufficient coverage to be represented at each point (125K raw reads, SS2 [*n* = 34], SSsc [*n* = 22], SS3 [*n* = 593], FS-25µl [*n* = 147], and FS-5µl [*n* = 608]). hPBMCs are divided by cell type (Azimuth, predicted.celltype.l2). Only conditions supported by >2 cells are displayed. For each cell type, the number of detected genes was compared between FS-5µL and SS3 at 125K downsampled raw reads using a Wilcoxon rank sum test (two-sided, Bonferroni correction, adj. P-value). The result of this comparison is displayed on top of each panel.

d. Estimated proportion of reads mapped to intronic or CDS-exonic features in HEK293T cells, in read tag percentages.

e. Same as (d) but for hPBMCs. No downsampling was performed for both (d) and (e).

Supplementary Fig. 4

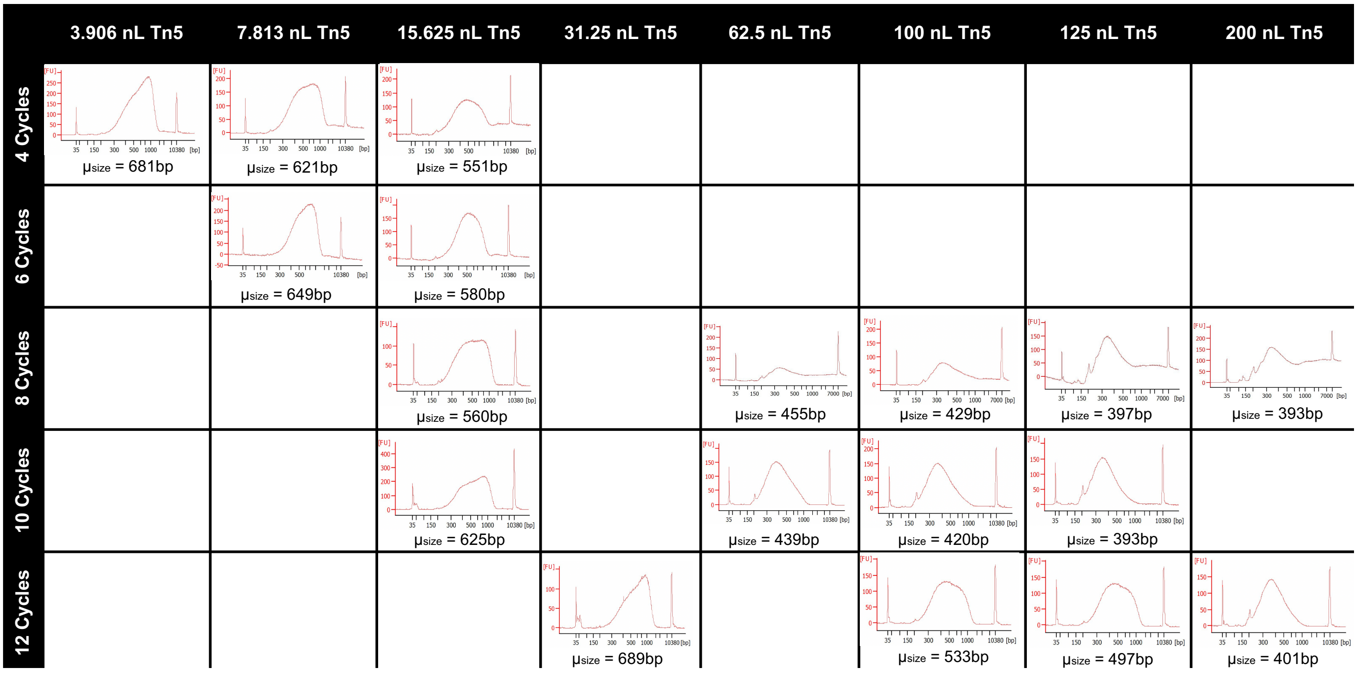

Supplementary Fig. 4

Impact of the number of PCR cycles and home-made Tn5 amount on FS-LA cDNA size distribution (HEK293T cells).

Bioanalyzer traces of sequencing-ready FS-LA libraries. The number of PCR cycles used for cDNA preamplification is reported on the rows (4-12 cycles). The amount of Tn5 added to each cell for tagmentation is reported on top of the chart. The mean cDNA size distribution between 200 bp and 9000 bp is displayed below each trace.

Supplementary Fig. 5

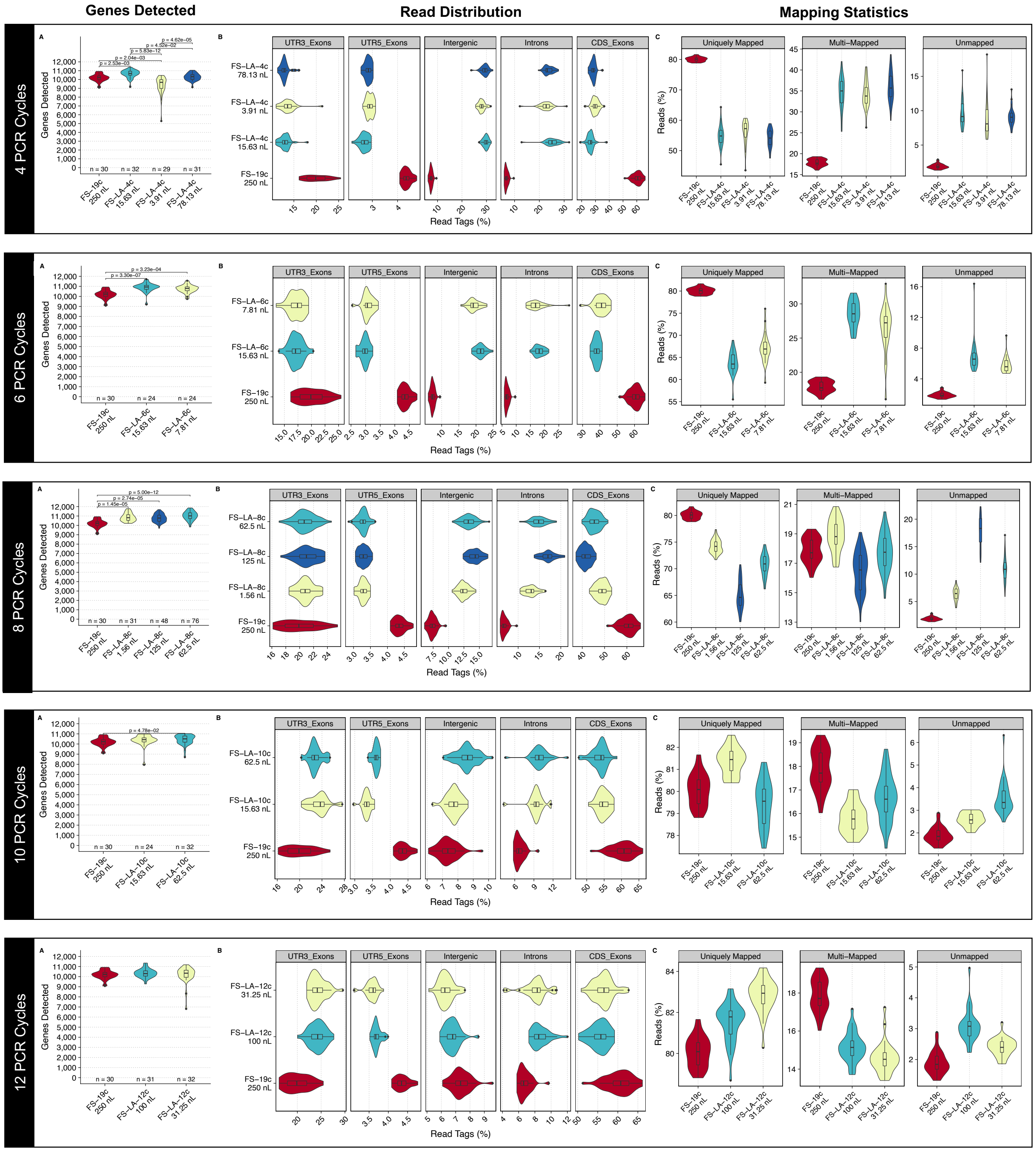

Supplementary Fig. 5

Overview of FS-LA gene detection and mapping statistics (HEK293T cells).

Each line compares standard FS (19 PCR cycles, FS-19c, in dark red) with FS-LA done with **1.** 4 PCR cycles (4c) **2.** 6 PCR cycles (6c) **3.** 8 PCR cycles (8c) **4.** 10 PCR cycles (10c) **5.** 12 PCR cycles (12c). The amount of homemade Tn5 in nanoliters is shown under each condition. Each line is composed of three panels: number of genes detected (> 0 reads), read distribution (ReSQC, reag tag percentage) and mapping statistics (STAR). 250K downsampled reads were used. Multiple comparisons of the number of detected genes were evaluated using Dunn's test (two-sided, Bonferroni adj. *P*-value).

Supplementary Fig. 6

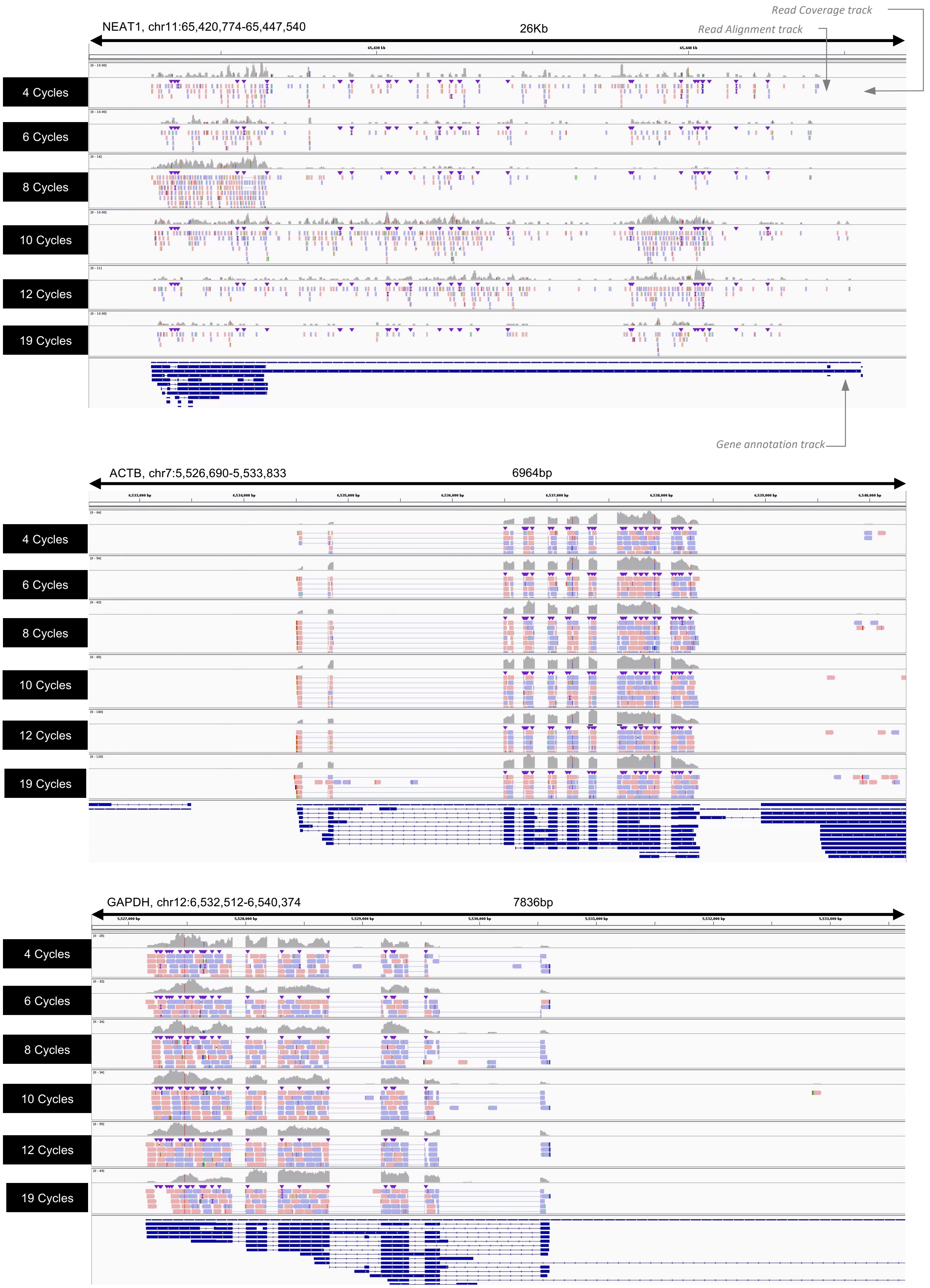

Supplementary Fig. 6

Integrated Genome Viewer (IGV) visualization of selected genes from HEK293T cells processed with FS-LA.

Each panel contains 6 representative cells processed with 4, 6, 8, 10 or 12 PCR cycles. Raw reads were downsampled to 500K and mapped onto hg38. As depicted in the first panel, each sample track consists of a gene coverage and a read mapping track. In the read mapping track, each blue or red bar corresponds to a single mapped read. The color of the bar indicates the read orientation compared to the reference. Fine lines highlight split reads. The gene annotation is displayed below each panel. Fine blue lines correspond to introns and bold blue lines to exons. Each panel shows a different gene (from top to bottom: lncRNA-NEAT1, ACTB, GAPDH).

Supplementary Fig. 7

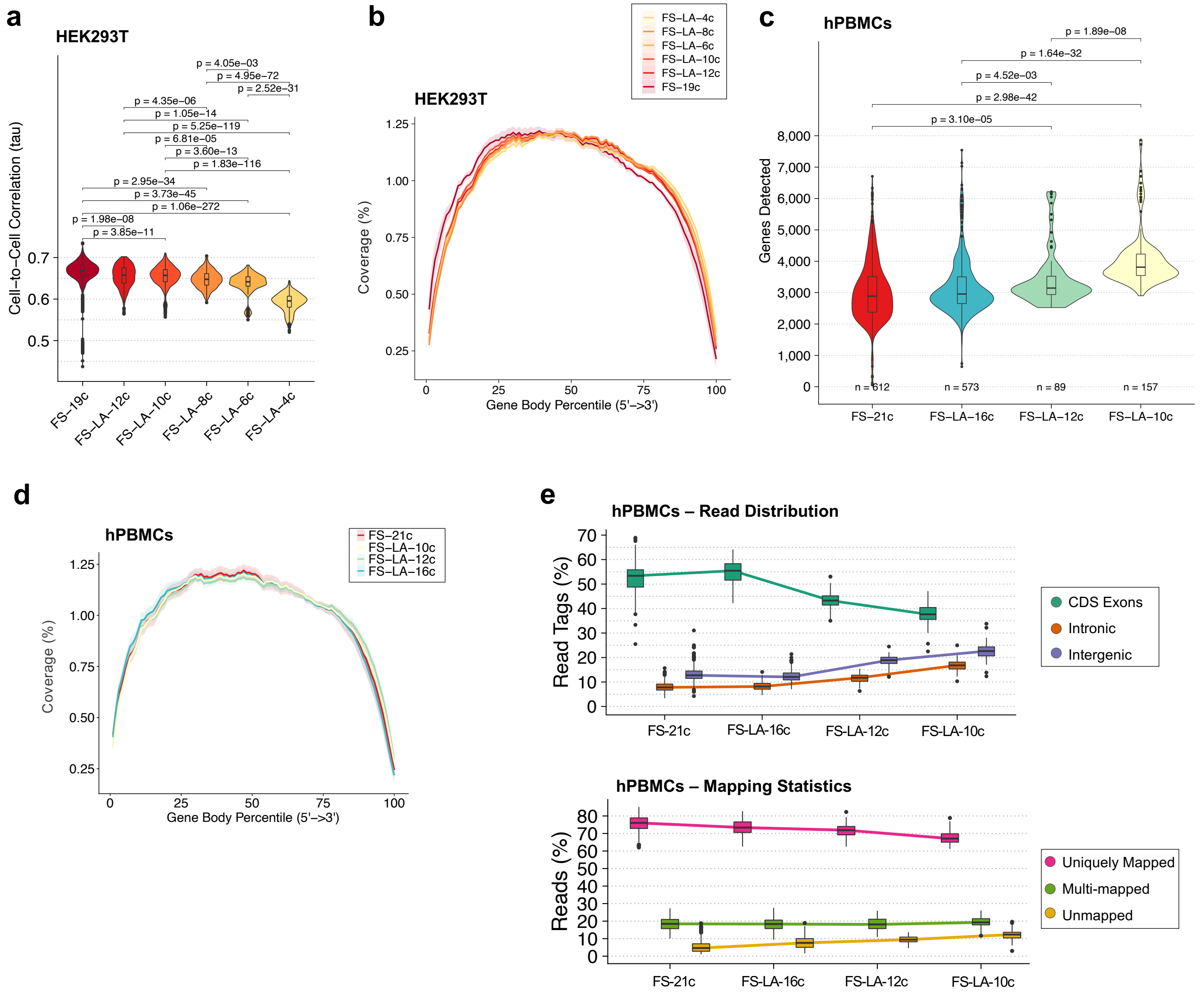

Supplementary Fig. 7

FS-LA Mapping statistics - complementary information to Figure 1.

a. Cell-to-cell Kendall's tau correlations between genes detected in FS and FS-LA on HEK293T cells, downsampled to 250K raw reads (Dunn's test, two-sided, Bonferroni correction, adj. *P*-value). The comparison was restricted to genes expressed with >0 reads in >2 cells in every condition (N_{genes} = 22,611).

b. Mean (± s.d.) gene-body coverages of standard FS (19 PCR cycles) and FS-LA protocols (4-12 PCR cycles) in HEK293T cells.

c. Number of genes detected in hPBMCs for standard FS ($n_{FS\text{-}21c}$=612) and FS-LA ($n_{FS\text{-}LA\text{-}16c}$=573, $n_{FS\text{-}LA\text{-}12c}$=89, $n_{FS\text{-}LA\text{-}10c}$=157) (Dunn's test, two-sided, Bonferroni correction, adj. *P*-value). Cells were downsampled to 250K raw reads.

d. Mean (±s.d.) gene-body coverages of hPBMCs processed with standard FS (21 PCR cycles) and FS-LA protocols (10-16 PCR cycles).

e. Read distribution and mapping statistics of hPBMCs processed with standard FS (21 PCR cycles) or FS-LA (10-16 PCR cycles) - Upper panel: proportion of reads mapped to exonic, intronic or intergenic features, measured using ReSQC, in read tag percentages. Lower panel: percentage of uniquely mapped, multi-mapped or unmapped reads for FS and FS-LA.

Supplementary Fig. 8

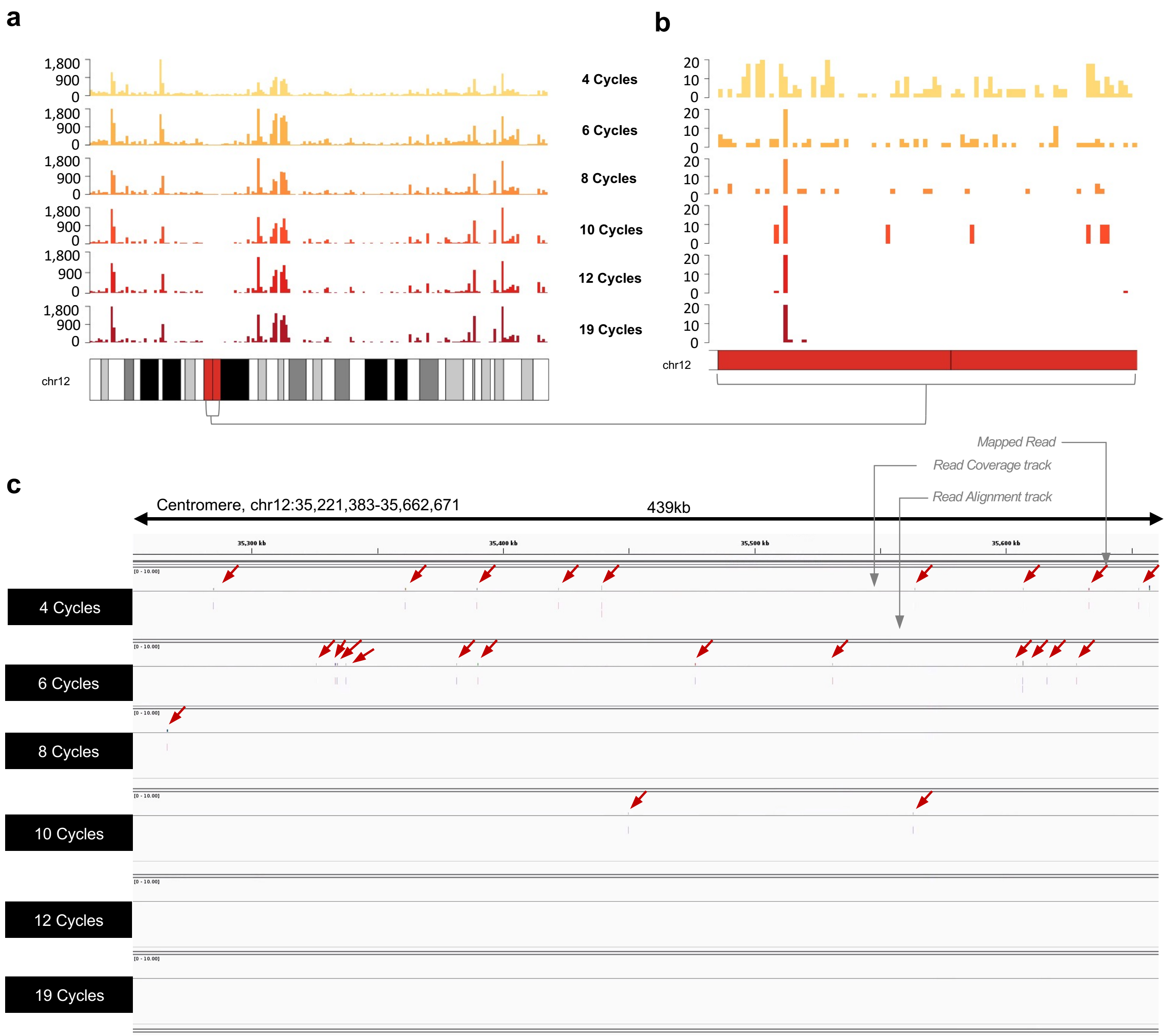

Supplementary Fig. 8

Chromosome and centromere-wide distribution of reads generated with FS-LA (HEK293T cells).

a. Distribution of mapped reads on chromosome 12, averaged to 750,000 bp windows for 6 representative samples, amplified with 4, 6, 8, 10 or 12 PCR cycles (FS-LA) or 19 PCR cycles (FS), respectively, all downsampled to 500K raw reads.

b. Zoomed view on the chromosome 12 centromeric region.

c. Integrated genome viewer screenshot showing an example of unexpected reads mapping on a 439 Kb region of the chromosome 12 centromere. Mapped reads are marked by a red arrow. Each line represents a different sample processed with 4, 6, 8, 10, 12 or 19 PCR cycles, respectively.

Supplementary Fig. 9

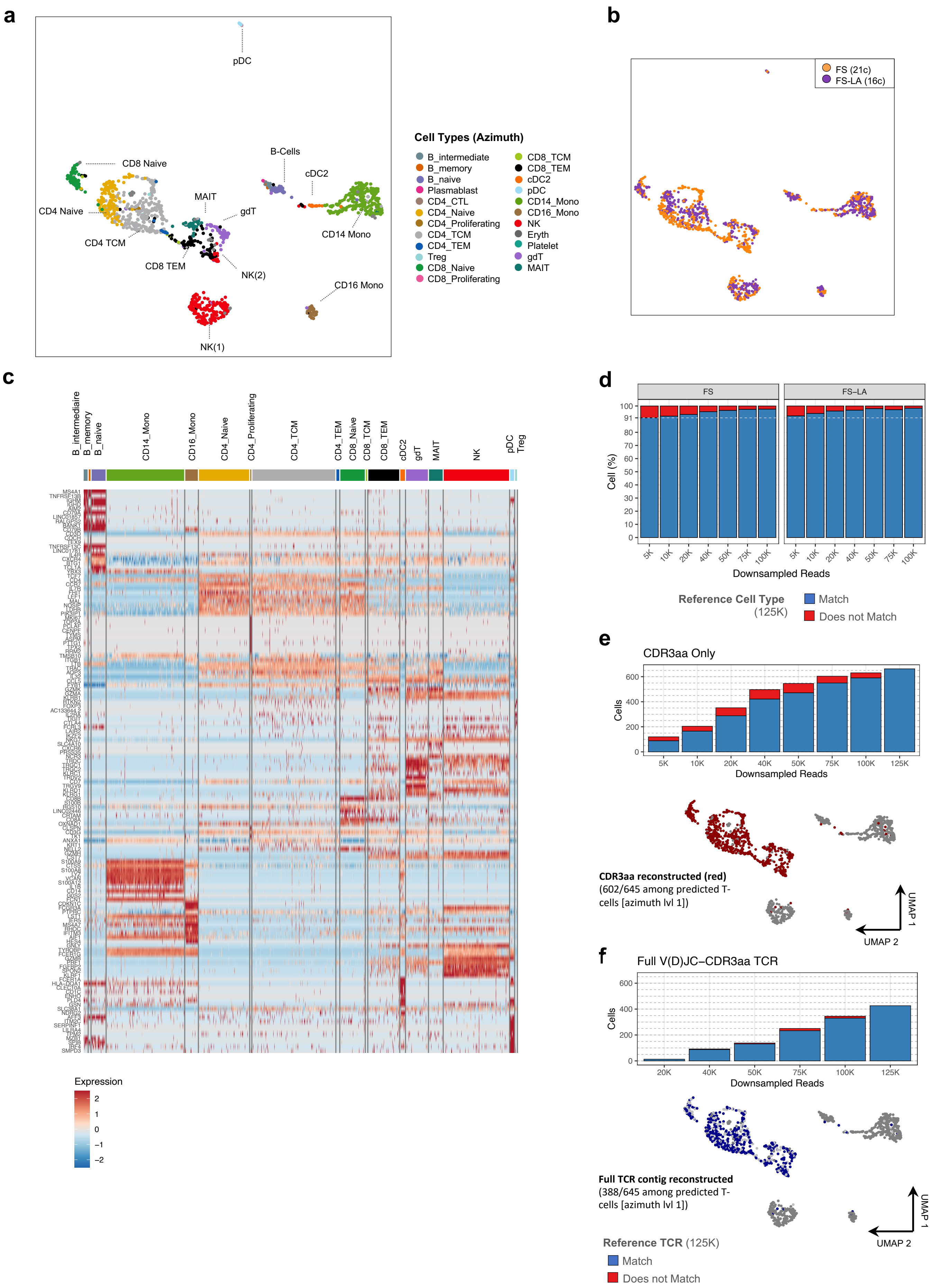

Supplementary Fig. 9

Analysis of FS and FS-LA hPBMCs data.

a. UMAP visualization of hPBMCs processed with FS (*n* = 612) and FS-LA (16 PCR cycles, *n* = 573), using 125K downsampled raw reads. Colored by cell-type (Azimuth, predicted.celltype.l2) or by **b.** method.

c. Heatmap of selected cell type marker genes in cells processed with either standard FS (21 PCR cycles, FS-21c, *n* = 612) or FS-LA (16 PCR cycles, FS-16c, *n* = 573). Marker genes were obtained from Azimuth (predicted.celltype.l2).

d. Stability of the predicted cell types at different downsampled read depths (5K, 10K, 20K, 40K, 50K, 75K, 100K raw reads) in both methods. Measured as the percentage of cells sharing the same predicted cell type (Azimuth, predicted.celltype.l2) as the 125K reads reference (blue) *vs* those which differ from it (red)

e. Upper panel: number of cells with a valid reconstructed CDR3aa sequence (TRUST4), at various downsampling depths, colored by cells with the same (blue) or different (red) CDR3aa as the 125K reference. Lower panel: UMAP visualization of the hPBMCs processed with FS and FS-LA, using 125K downsampled raw reads, colored by the reconstruction of the CDR3aa sequence using TRUST4.

f. Same as (d) but for reconstruction of the full V(D)JC-CDR3aa sequence.

Supplementary Fig. 10

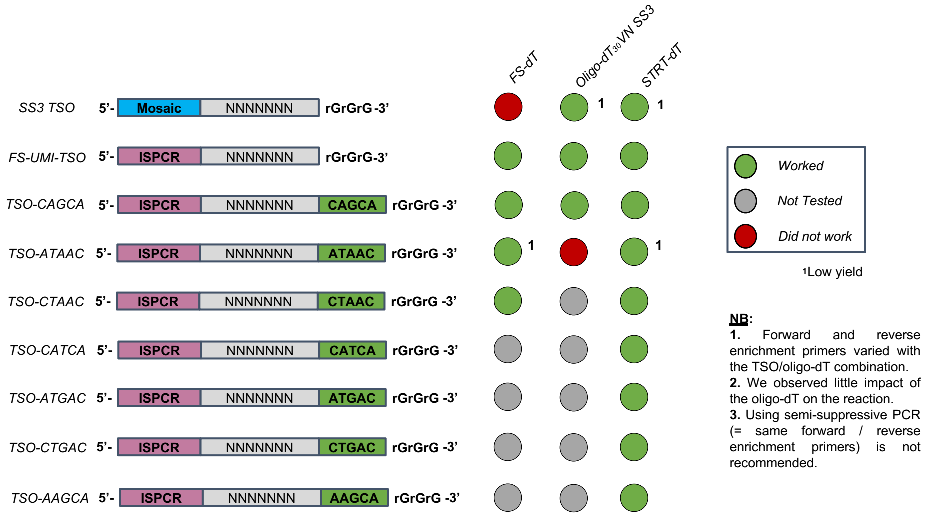

Supplementary Fig. 10

Oligo-dT / TSO combinations tested in this study.

Combinations of TSO and oligo-dT tested in this study. Not all successful libraries were sequenced.

Supplementary Fig. 11

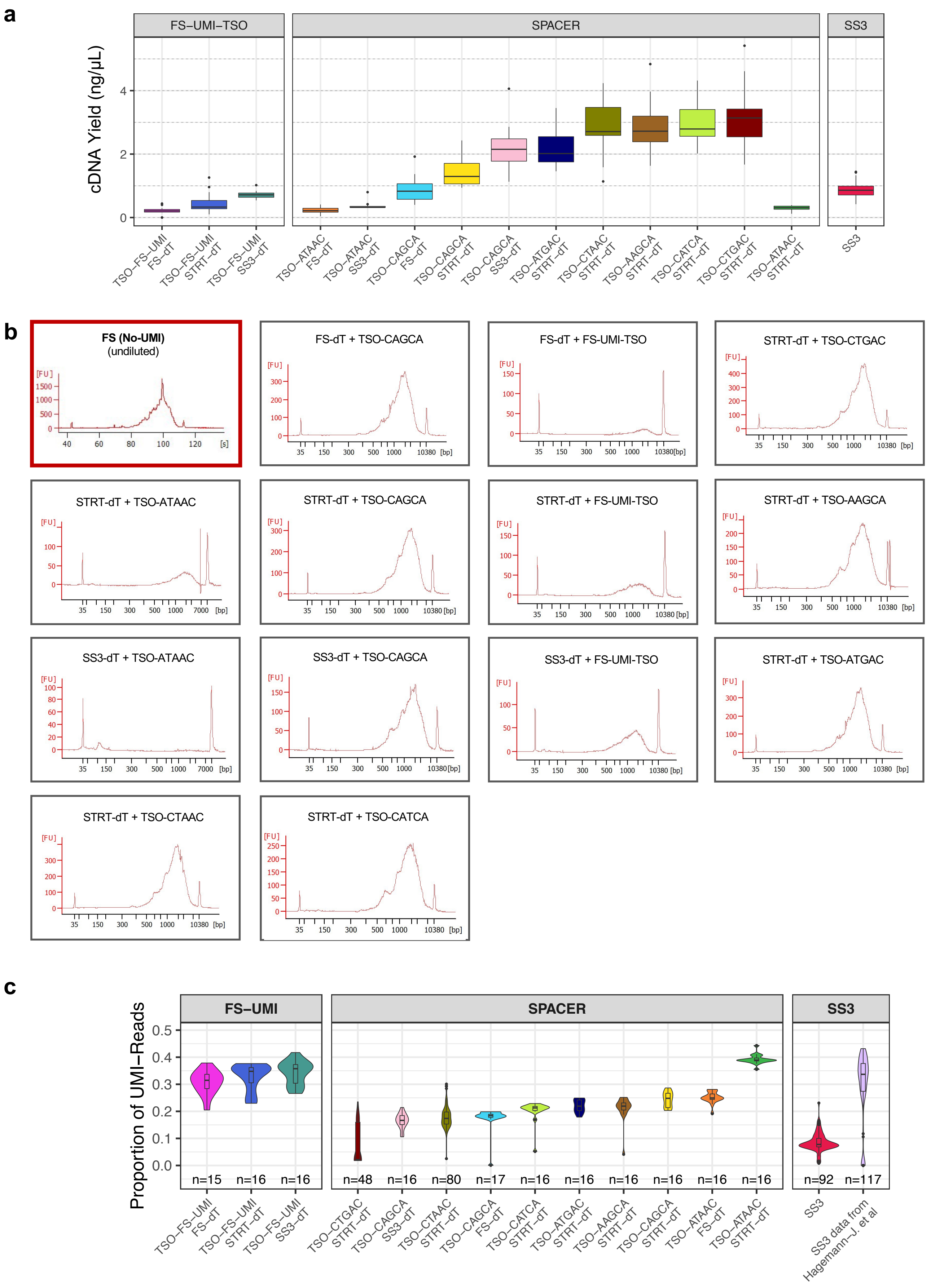

Supplementary Fig. 11

cDNA yield and length distribution of UMI-TSO / oligo-dT combinations.

a. cDNA yields.

b. Bioanalyzer traces of a representative sample from selected UMI-TSO / oligo-dT combinations. Control FS performed using a standard TSO (= without UMI) is highlighted in red.

c. Proportion of UMI reads among the different sequenced combinations of TSO-UMI / oligo-dT.

Supplementary Fig. 12

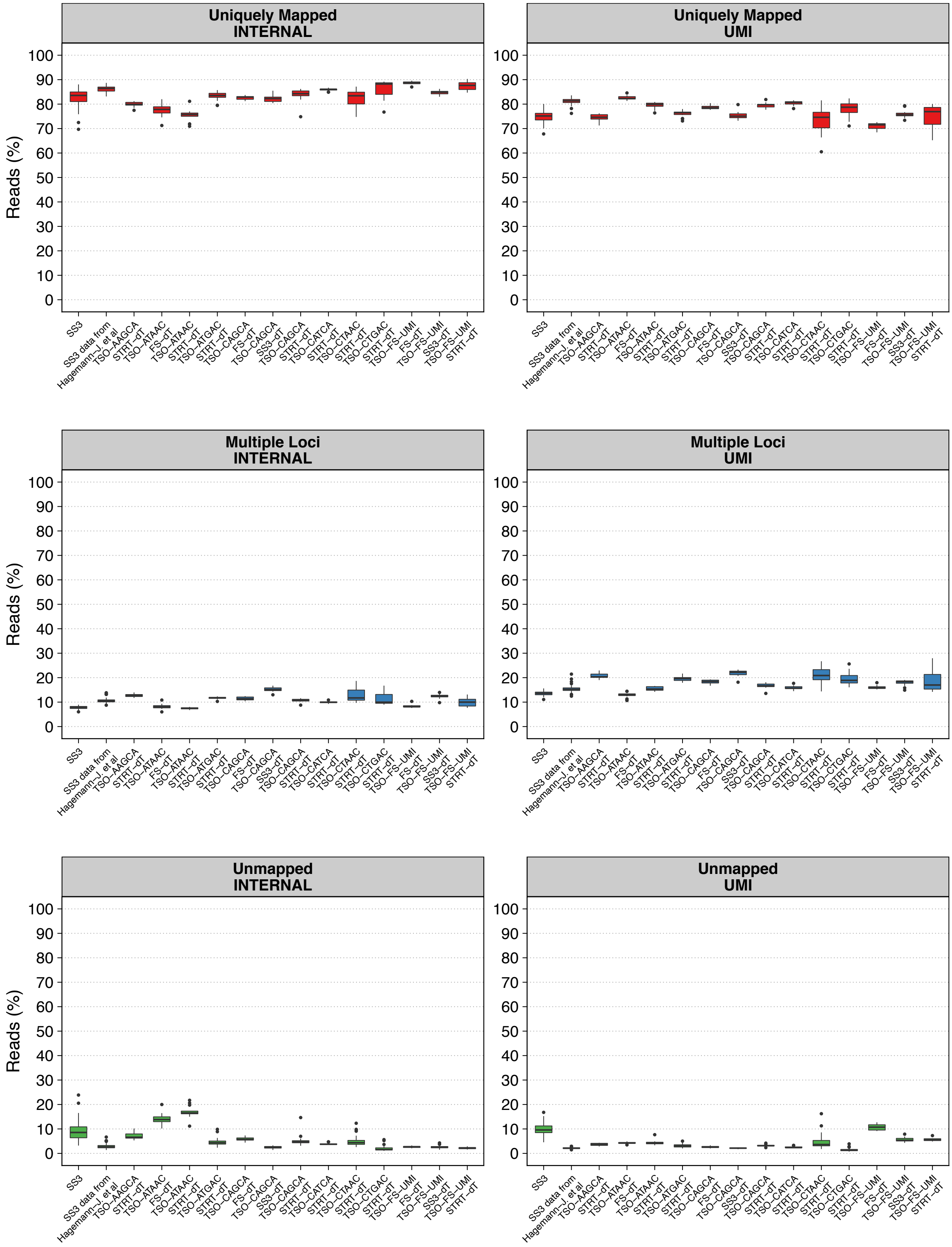

Supplementary Fig. 12

Internal and UMI reads mapping statistics of selected UMI-TSO / oligo-dT combinations. STAR mapping statistics showing the percentage of uniquely mapped, multi-mapped and unmapped reads using either internal or UMI-reads.

Supplementary Fig. 13

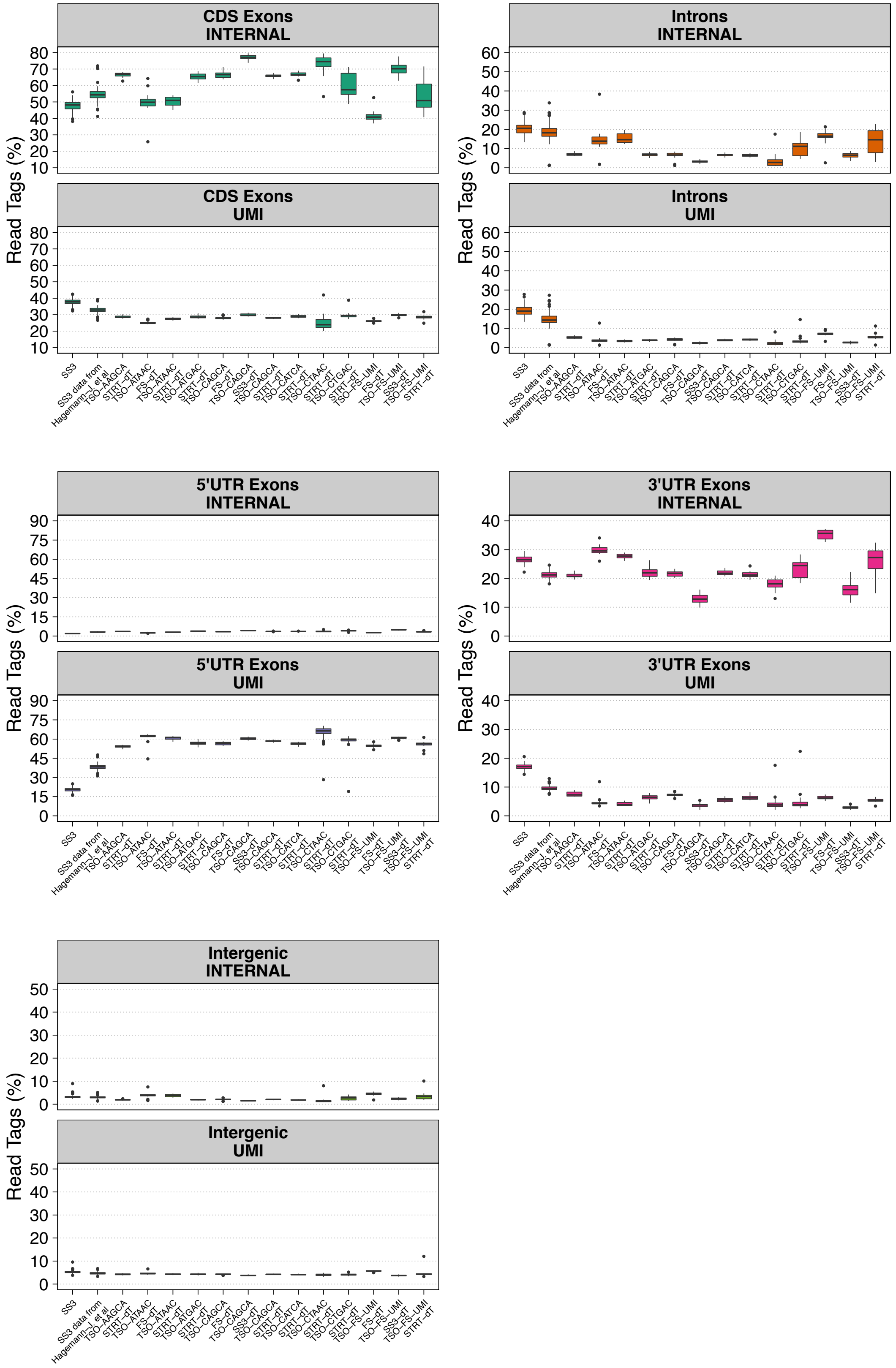

Supplementary Fig. 13

Mapped read distribution in FS-UMI and SS3. Distribution of mapped internal- or UMI-reads between introns, intergenic regions, 3'-UTR, 5'-UTR or coding sequence (= CDS). Expressed in percentage of read tags, computed with ReSQC.

Supplementary Fig. 14

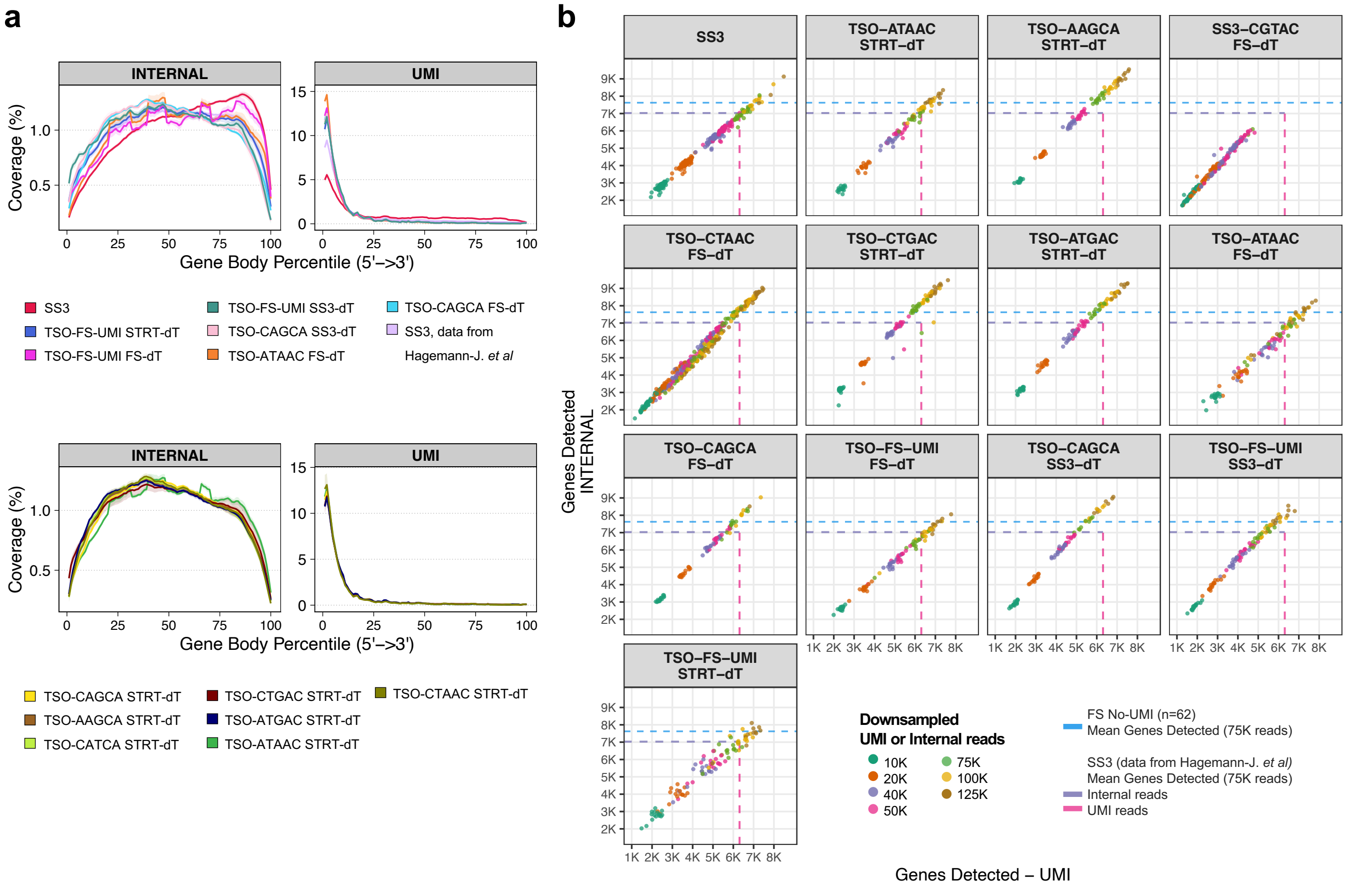

Supplementary Fig. 14

FLASH-seq with UMI protocol, complementary information.

a. Mean (± s.d.) gene-body coverages for internal (left) and UMI reads (right), split in 2 separate panels to facilitate readability.

b. Relationship between the number of genes detected using UMI- and internal-reads. UMI- and internal-reads were downsampled to 10K, 20K, 40K, 50K, 75K, 100K, 125K reads. Dashed blue lines represent the mean number of genes detected in HEK293T cells processed with FS-5µl (*n* = 85) and downsampled to 75K raw reads. Dashed purple and pink lines represent the mean number of detected genes from HEK293T cells generated by Hagemann-Jensen *et al.*, using UMI (pink) or internal (purple) reads, downsampled to 75K raw reads.

Supplementary Fig. 15

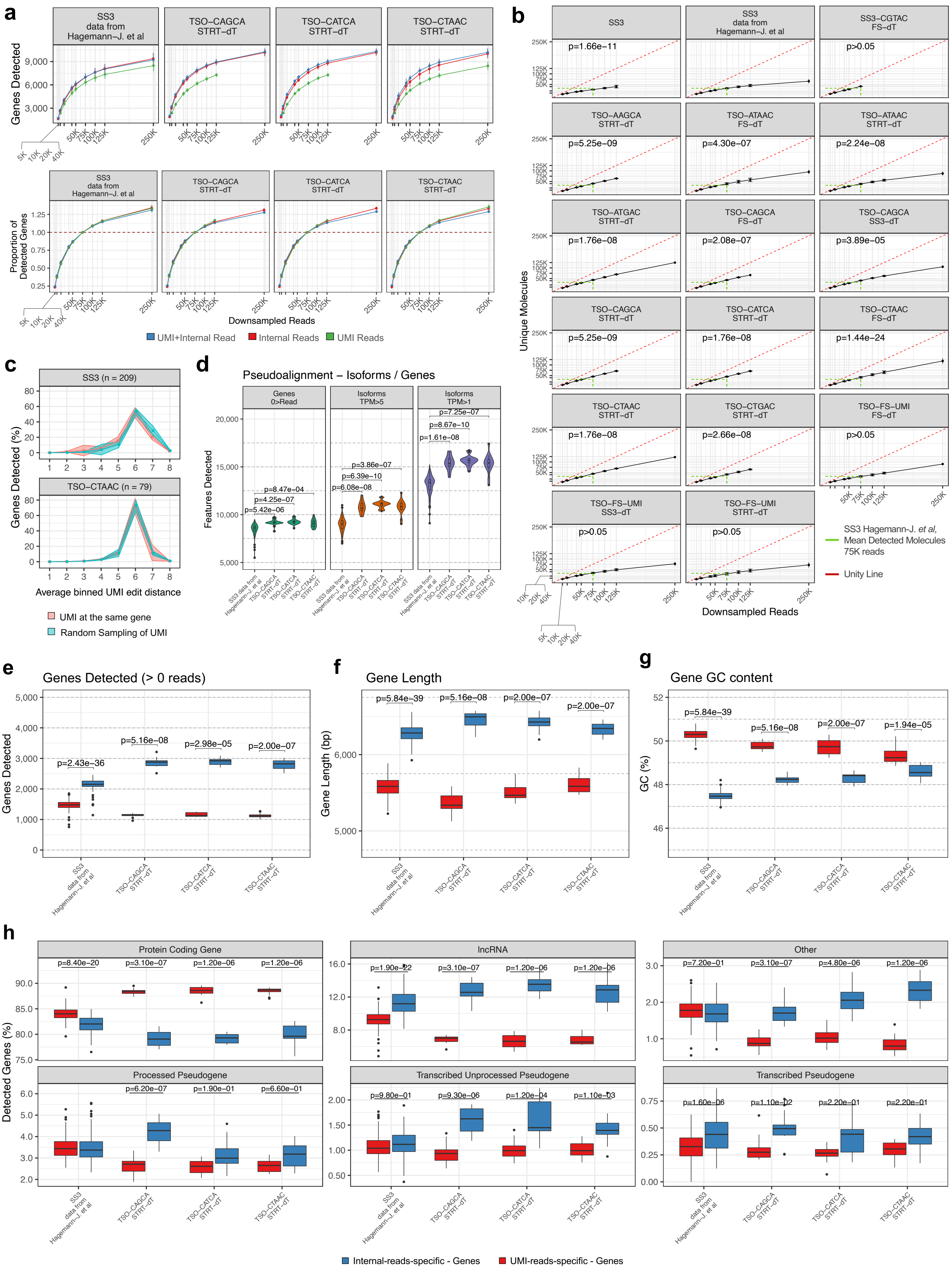

Supplementary Fig. 15

Comparison of UMI / Internal reads properties in SS3 and selected FS-UMI conditions.

a. Number of genes detected (>0 reads) using UMI, internal or both read types (top) at various sequencing depths. Proportion of genes detected (bottom), defined based on the number of genes detected at 75K raw downsampled reads (red line).

b. Mean (± s.d.) number of unique molecules detected with deduplicated UMI reads at seven downsampling read depths. Red lines mark the ideal case where every UMI read is a unique molecule. Green lines represent the mean number of unique molecules detected at 75K raw reads in HEK293T data from SS3 Hagemann-Jensen *et al.* . This value was compared to each group with a two-sided Wilcoxon rank sum test (Bonferroni correction, adj. P-value < 0.05 displayed).

c. Mean (± s.d.) binned edit distance of UMI post-deduplication in UMI associated to the same gene (blue) and UMI resampled from other genes (red) in FS-UMI (TSO-CTAAC) or SS3 (data from this manuscript and from Hagemann-Jensen *et al.*). No downsampling.

d. Number of features detected in a subset of conditions with pseudoaligment (salmon). Summarized at the gene- or isoform-feature level (*tximport*). Both UMI- and internal-reads were used (downsampled to 250K reads). Isoforms at two detection thresholds (library size scaled TPM >1 or >5). Statistical significance is evaluated with a two-sided Dunn's test with SS3 data from Hagemann-Jensen *et al.* as reference (Bonferroni correction, adj. P-value).

e. Properties of the genes detected (>0 reads) with 75K randomly selected internal- or UMI reads (two-sided Wilcoxon rank sum test, Bonferroni correction, adj. *P*-value).

f. Same as (e) but reporting the gene length.

g. Same as (e) but reporting the gene GC-content (%).

h. Same as (e) but subdividing the detected genes by gene types, in percentage of detected genes.

In downsampling figures (a-b), the number of cells may vary due to insufficient number of reads.

(a, d-h) graphics were made using $n_{SS3\_Hagemann\text{-}Jensen} = 117$, $n_{TSO\text{-}CTAAC} = 15$, $n_{TSO\text{-}CAGCA} = 16$, $n_{TSO\text{-}CATCA} = 15$.

Supplementary Fig. 16

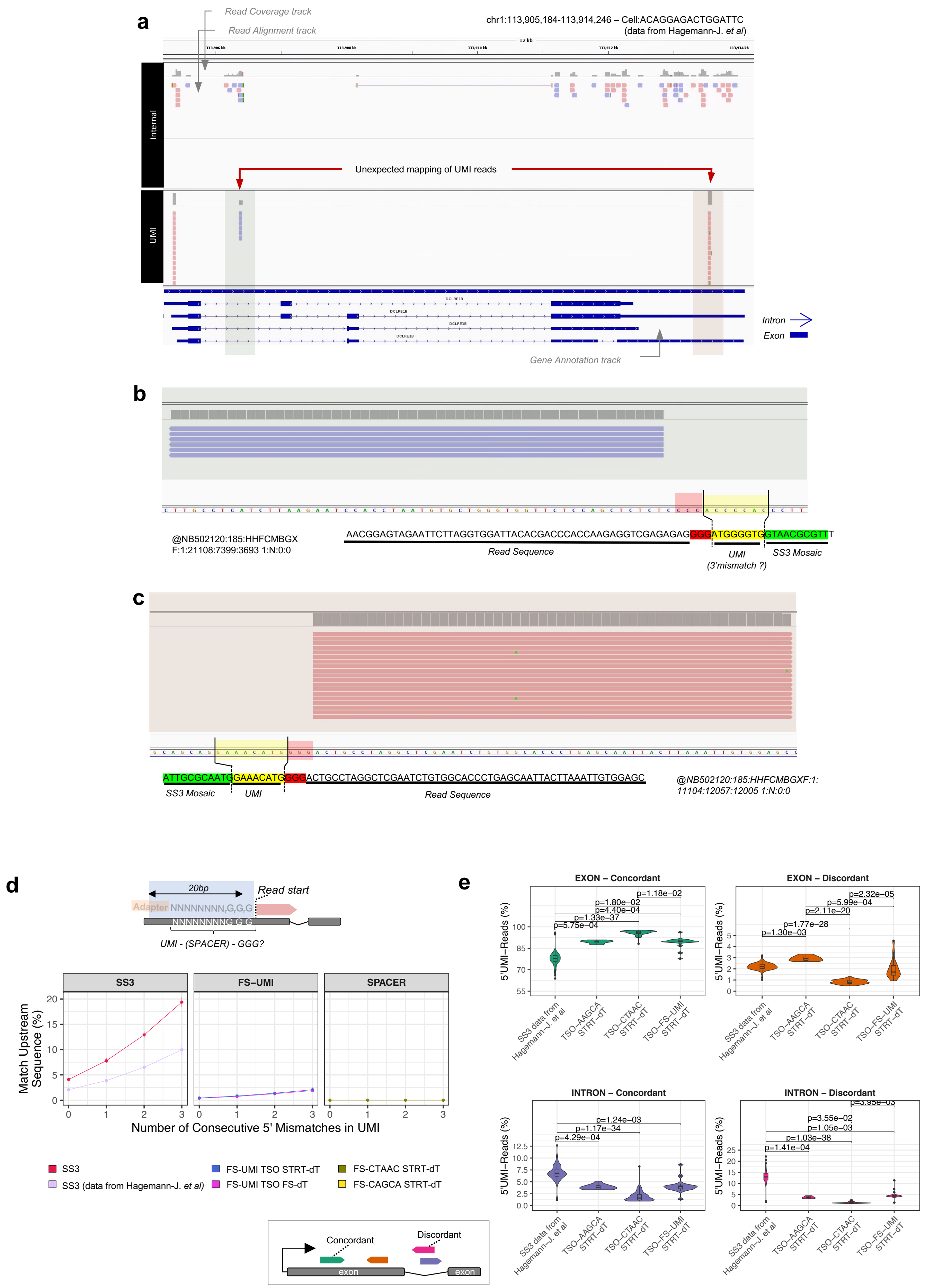

Supplementary Fig. 16

Strand-invasion.

a. Integrated Genome Viewer snapshot showing the reads of a HEK293T cell 'ACAGGAGACTGGATTC' (data from Hagemann-Jensen *et al.*) mapping to gene DCLRE1B. The upper part of the picture shows internal reads (= no UMI). The bottom part displays 5' UMI reads. Two piles of PCR duplicated 5' UMI reads are located within the gene-body rather than at the 5' end.

b. Zoomed view on the first read pile (green in [a]). These reads are located in an intronic sequence and are in discordant orientation with respect to the gene. The read sequence is displayed below. The read's GGG motif and UMI show almost a perfect complementarity with the genomic sequence.

c. Same as (b) but zooming on the second pile of PCR duplicated reads (red in [a]). The reads are in concordant orientation with respect to the gene. A perfect match between the GGG motif and UMI the genomic sequence is observed.

d. Percentage of deduplicated 5' UMI reads harboring a match between the UMI-(SPACER)-rGrGrG pattern and the upstream sequence within 20-bp, with 0 mismatch or 1-3 consecutive 5' mismatches (n_{SS3} = 2,089,581, $n_{SS3\_Hagemann\text{-}Jensen.}$ = 13,511,157, $n_{FS\text{-}UMI\text{-}TSO\_STRT\text{-}dT}$ = 1,404,599, $n_{FS\text{-}CTAAC\_STRT\text{-}dT}$ = 9,964,516, $n_{FS\text{-}CAGCA\_STRT\text{-}dT}$ = 1,189,676, deduplicated UMI reads). Colored by oligo-dT / TSO combination.

e. Distribution of UMI reads on exonic / intronic sequences, in selected conditions, colored by feature type (exon / intron) and read orientation compared to the gene. In percentage of UMI reads unambiguously assigned to a gene in an unstranded manner (n_{SS3} = 2,438,862, $n_{SS3\_Hagemann\text{-}Jensen.}$ = 17,494,648, $n_{FS\text{-}CTAAC\_STRT\text{-}dT}$ = 9,491,378, $n_{FS\text{-}CAGCA\_STRT\text{-}dT}$ = 1,166,430, UMI reads) (Dunn's test, two-sided, Bonferroni correction, adj. P-value < 0.05 displayed).

Supplementary Fig. 17

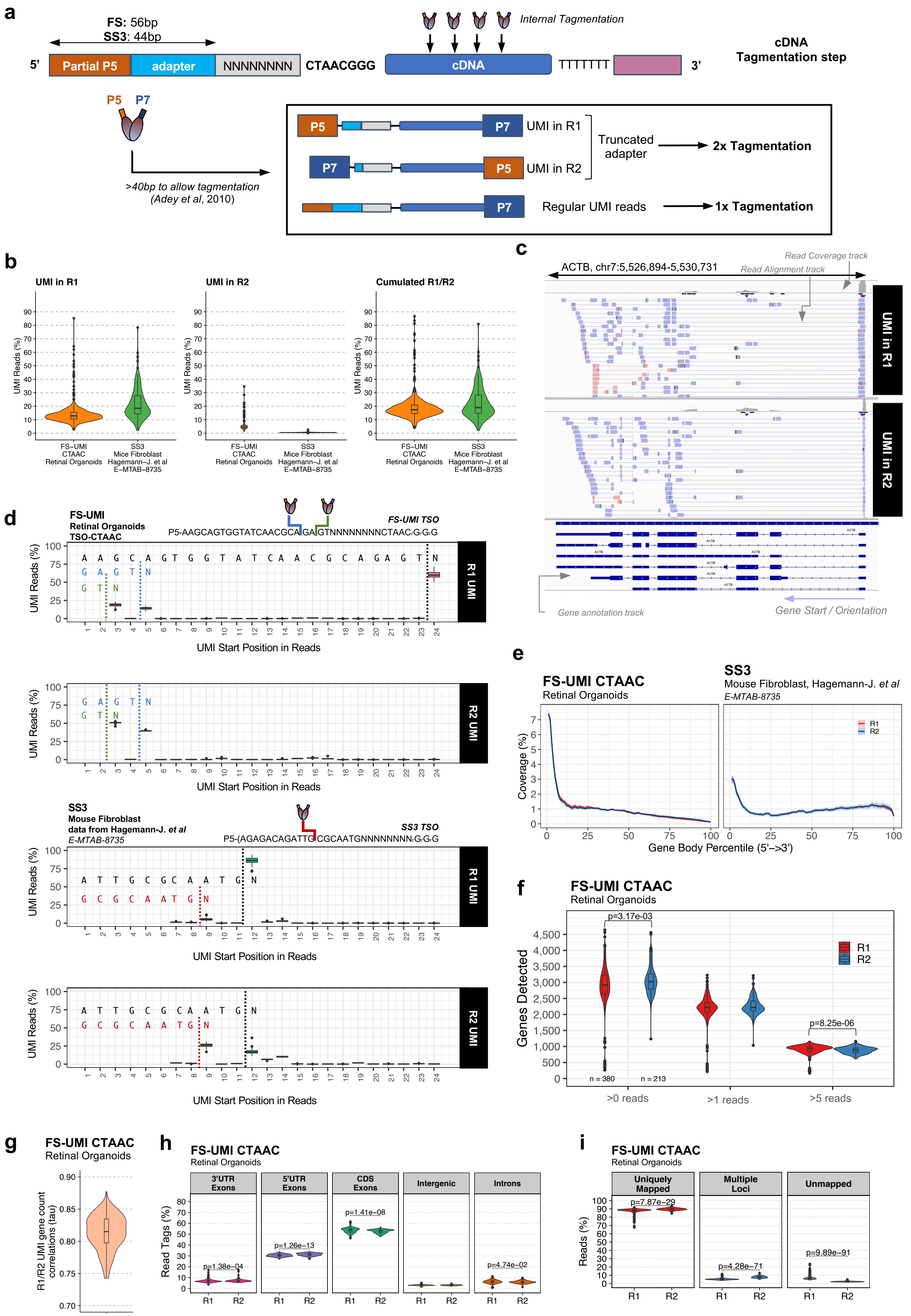

Supplementary Fig. 17

UMI information in read 2 from retinal organoids cells, plate #316.

a. Schematic representation of the 5' tagmentation process. The long 5' adapter sequence (TSO + partial P5) sequence in FS-UMI (56 bp) and SS3 (44 bp) can be cut by Tn5 which inserts either a P5 or P7 sequence at a random position upstream of the UMI. This results in a truncated 5' adapter sequence. If a P7 sequence is inserted, the UMI will be found in read 2 (= R2) instead of read 1 (= R1).

b. UMI reads (%) in R1, R2 or cumulated R1+R2 in FS-UMI (TSO-CTAAC, n = 380) or SS3 (n = 150, data from Hagemann-Jensen *et al*. 'Mouse Fibroblast Gel Cut').

c. Integrated Genome Viewer screenshot of R1-UMI (top) and R2-UMI (bottom) reads from FS-UMI, mapping on the gene ACTB. The majority of the reads are starting at the 5' of the gene. The color of the bar indicates the read orientation compared to the reference. Fine lines highlight split reads. The gene annotation is displayed below. Fine blue lines correspond to introns while bold blue lines represent exons.

d. Distribution of the UMI start position, in R1-UMI or R2-UMI for FS-UMI CTAAC (top) or SS3 (bottom), in 50 randomly selected cells per group. TSO sequence is shown above, with the most abundant Tn5 cuts in the 5' adapter highlighted.

e. Mean (±s.d.) gene body coverage of R1-UMI and R2-UMI reads in FS-UMI CTAAC or SS3 using reads trimmed to 30 x 30 bp to account for the difference in R1-UMI / R2-UMI read lengths.

f. Gene counts using deduplicated R1-UMI or R2-UMI in FS-UMI CTAAC or R1-UMI in SS3 at three thresholds (>0, >1 or >5 reads).

g. Kendall's tau correlation of the deduplicated R1-UMI *vs* R2-UMI gene counts obtained in each FS-UMI cell.

h. Percentage of read tags in 5' UTR, 3' UTR, exonic, intronic or intergenic features measured using ReSQC, for FS-UMI CTAAC R1-UMI, R2-UMI or SS3 R1-UMI.

i. Mapping statistics of R1-UMI and R2-UMI. Subdivided in uniquely mapped, multi-mapped or unmapped reads.

Analysis displayed in panels e-h were performed using 30K R1 and R2 UMI reads. (f-h) pairwise comparisons were performed using a Wilcoxon-rank sum test (Bonferroni correction, adj. *P*-value).

Supplementary Fig. 18

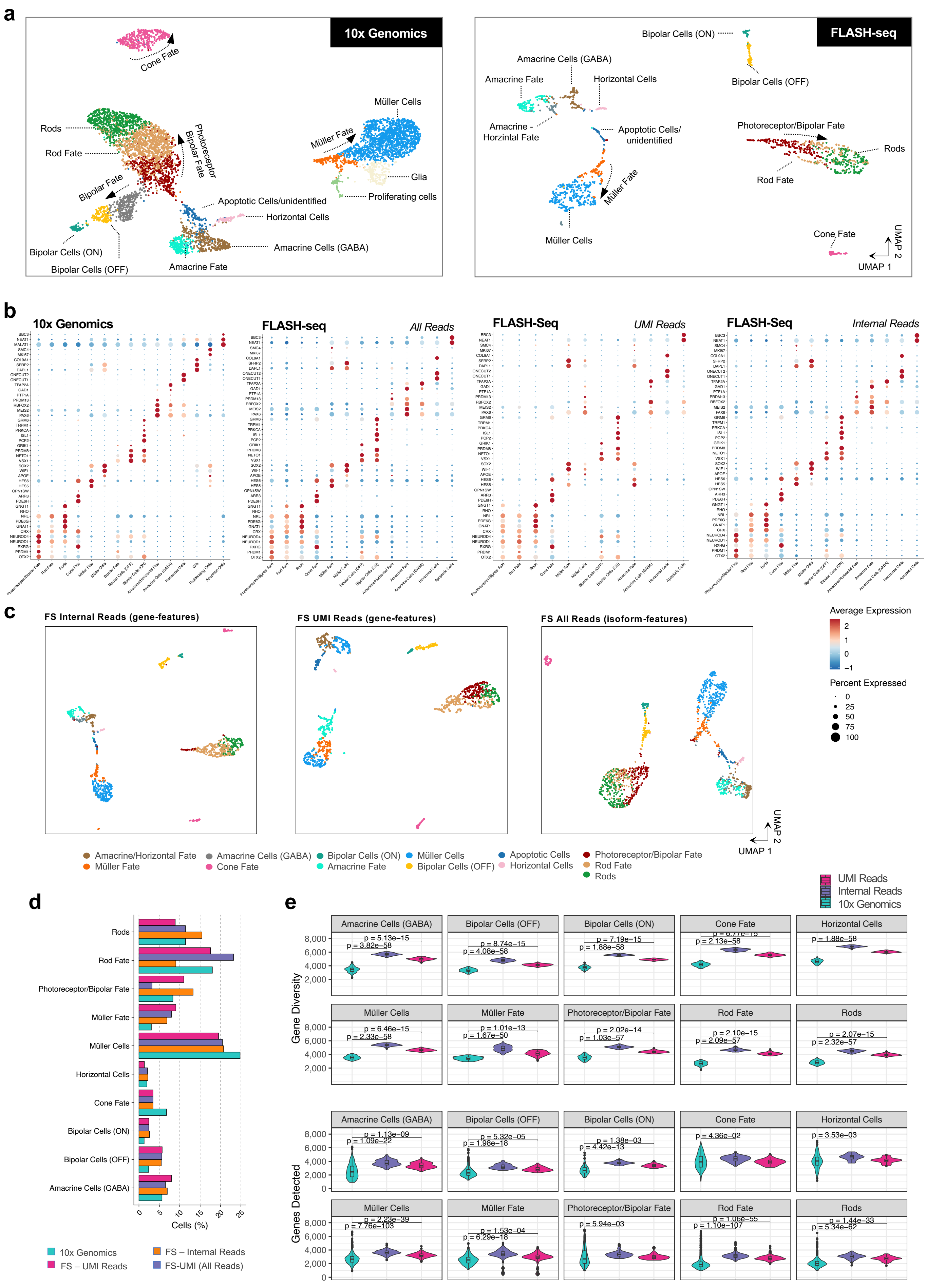

Supplementary Fig. 18

Cell types and gene diversity in retinal organoids processed with FLASH-seq and 10x Genomics 3' GEM kit.

a. UMAP of retinal cells from week-18 retinal organoid cells processed with 10x Genomics 3' GEM kit (left, *n* = 5561 cells) or FLASH-seq-UMI (right, UMI and internal reads combined, *n* = 1281)**.** Colored by cell-type.

b. Dotplots of selected cell type markers detected in retinal organoid cells processed with 10x Genomics 3' GEM kit or the FS-UMI protocol, subdivided into markers detected with UMI-reads only, internal-reads only or both read types.

c. UMAP of week-18 retinal organoid cells processed with FS-UMI and colored by cell-type. Generated using Internal reads (left) or UMI reads (middle) summarized at the gene-feature level. Cell types were manually annotated in both conditions. UMAP (right) generated using both read types aggregate at the isoform-feature level. Cell types from the isoform-feature level were directly transferred from the all-reads gene-feature level (Fig [Supplementary 18a]).

d. Detected cell-types in FS-UMI (internal-reads, UMI-reads or both) and 10x Genomics, in percentage of the total number of cells.

e. Gene diversity (upper panel) and number of genes detected (lower panel) in 10 representative cell-types. FS-UMI cells were downsampled to 33,800 downsampled raw reads to match the mean number of raw reads per cell in 10x Genomics. Gene diversity is defined separately in each cell-type by resampling 10 cells 100 times and calculating the number of genes expressed with >0 read, either UMI- or internal- reads, in >2 cells. Statistical significance is evaluated with a two-sided Dunn's test (Bonferroni correction, adj. *P*-value < 0.05 displayed).

Supplementary Fig. 19

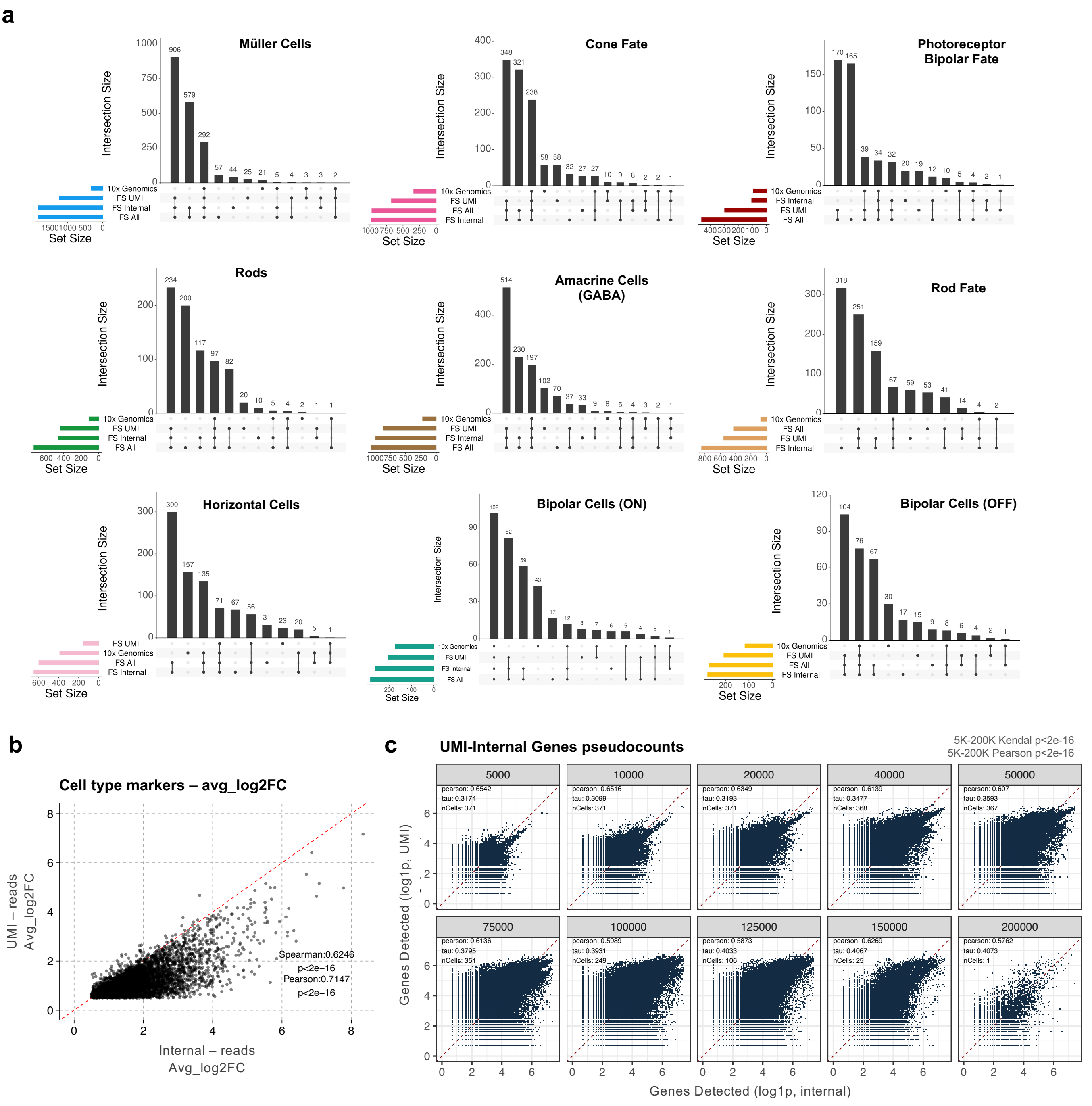

Supplementary Fig. 19

Cell type markers in retinal organoids and UMI-internal read counts relationship.

a. Upset plot displaying the overlap between cell-type markers obtained with FS-UMI (UMI-, internal- or both reads) and 10x Genomics, in 9 representative cell types.

b. Relationship between the cell-type markers's average log2-fold changes obtained with UMI- or internal-reads. The red line marks a theoretical identical relationship between the average log2-fold from both read types. Spearman and Pearson correlation coefficients are displayed.

c. Relationship between UMI- and internal-reads gene pseudocounts (log1p) at 8 raw read downsampling intervals (Plate #316, maximum n_{Cells}=371). The number of cells, Pearson correlation coefficient and Kendall's tau correlation coefficient are indicated on the top left corner of each panel.

Supplementary Fig. 20

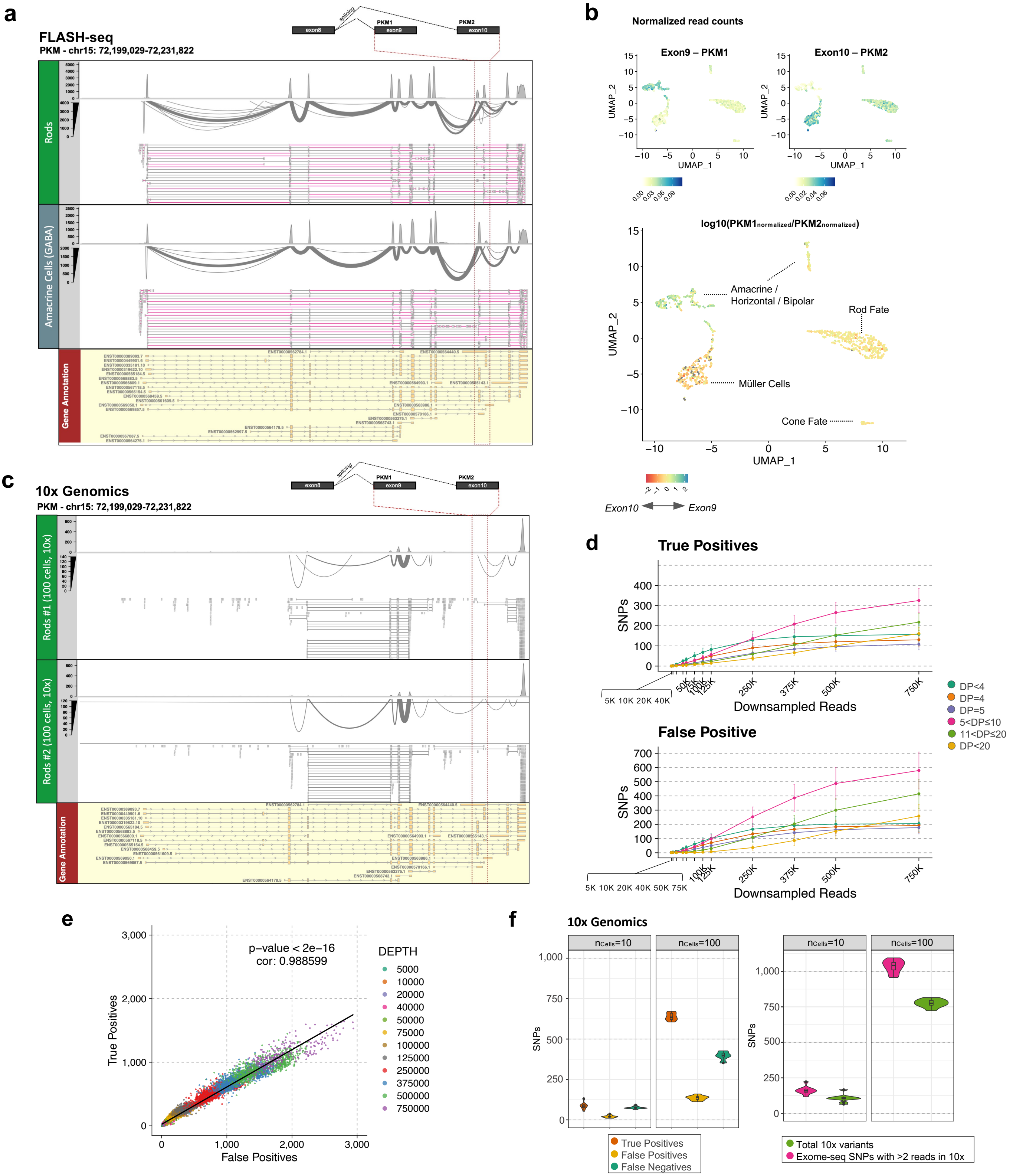

Supplementary Fig. 20

PKM Isoform expression and single nucleotide polymorphisms in FS-UMI and 10x Genomics.

a. Reads mapping to the PKM gene in 10 randomly selected rods (top) or amacrine (GABA) cells (mid) processed with FS-UMI. Isoforms of the gene depicted below (bottom). Defining features of PKM1 (= exon9) and PKM2 (= exon10) isoforms are shown at the top. Each cell-type panel is composed of the read coverage, a sashimi plot and a read mapping track. Read mapping track contains mapped reads (gray blocks), splicing (gray lines) and read-mates (pink lines).

b. UMAP of FS-UMI retinal organoid cells colored by PKM exon 9 (upper left), exon 10 (upper right) normalized coverages or the log10 ratio of both exon coverages (bottom). Exon coverages were normalized by the number of reads mapping to the entire PKM gene in each cell.

c. Reads mapping to the PKM gene in 100 randomly selected rods (μ_{mapped_reads} = 2536K±111K) processed with 10x Genomics. Gene's isoforms depicted below (bottom).

d. Evolution of the number of true positive (top) or false positives (bottom) single nucleotide polymorphisms (SNPs) as a function of the number of downsampled raw reads (5K-750K). Colored by the number of reads supporting the variant (= DP).

e. Relationship between the number of true and false positives SNPs. Colored by the number of downsampled reads. Pearson correlation coefficient.

f. SNPs detected in 10 or 100 randomly selected rods processed with 10x Genomics (10-times resampling). All cells were combined as a unique sample. Colored by variant category. Left, variants detected in 10x Genomics and exome sequencing (= true positive, DP>2 [orange]), variants detected in scRNA-seq but not in exome-sequencing (= false positives, DP > 2 [yellow]) or undetected but transcribed variants (> 2 reads, = false negative [green]). Right, total variants: exome-sequencing variants in transcribed exons detected in 10x Genomics (> 2 reads, [pink]) or number of detected variants per cell (green).

Supplementary Fig. 21

BD FACSDiva 8.0.2

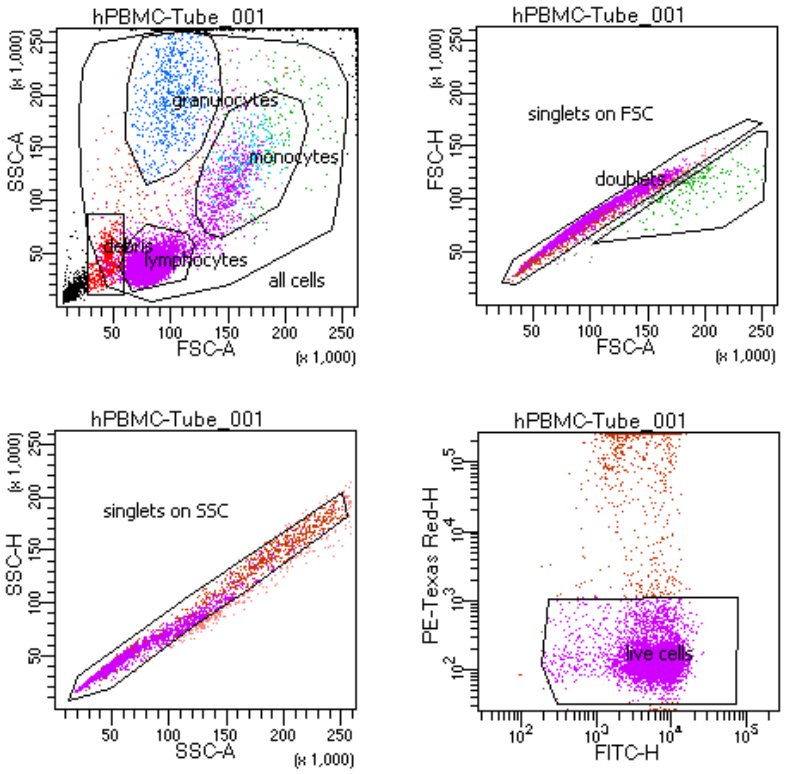

Tube: Tube_001

Population	#Events	%Parent	%Total
All Events	10,000	####	100.0
all cells	8,028	80.3	80.3
singlets on FSC	7,727	96.3	77.3
singlets on SSC	7,439	96.3	74.4
live cells	6,231	83.8	62.3
doublets	163	2.0	1.6
lymphocytes	5,484	54.8	54.8
monocytes	895	9.0	9.0
debris	634	6.3	6.3
granulocytes	690	6.9	6.9

Supplementary Fig. 21

hPBMCs FACS gating strategy.

Representative flow cytometry analysis of CD45+PI- hPBMC. Particles smaller than cells (debris) were eliminated with an area plot of forward-scatter (FSC-A) *vs* side-scatter (SSC-A) by gating for cell-sized particles inside the gate (upper-left panel). A 2-steps doublet discrimination was carried out to remove cell aggregates by making plots of area *vs* height, both in the forward- and side-scatter channel, FSC-A *vs* FSC-H and SSC-A *vs* SSC-H (upper-right and lower-left panels, respectively). For the hPBMCs we plotted FITC-H (for CD45) *vs* TexasRed-H (for PI), sorting all singlets that were FITC+PI- (lower-right panel).

Supplementary Discussion

Supplementary Data #1

In the following paragraphs we summarize the results of the tests we performed to establish FLASH-seq (FS), reporting only those that we considered of sufficient quality for sequencing. We evaluated the benefits of new additives and reaction conditions, by using HEK 293T cells as well as more challenging cells, such as human peripheral blood mononuclear cells (hPBMCs), which better reflect a real scientific experiment than cell lines. The cell type used in each experiment and the reaction volume (25 µl or 5 µl) are indicated in square brackets. We seeked to develop a protocol that was fast, affordable and that could provide excellent gene detection even in the most challenging cells. Whenever possible, we compared gene expression between conditions on cells sorted on the same plate or, at least, belonging to the same batch, to minimize potential batch effects. All comparisons were performed using 100K downsampled raw reads, unless stated otherwise. The gene expression threshold was set to >0 read. Only *P*-values < 0.05 or adjusted *P*-values < 0.05 are displayed.

Box Plots:

- Center: Median.
- lower/higher hinges: 25th percentile, median, 75th percentile.
- Whiskers: 1.5 interquartile range.
- Points: outliers.

Condition tested	Effect	Comment
**Lysis Mix**		
1.2% Triton		Increases the amount of multi-mapped reads
0.2% Triton		Optimal condition
1 mg/mL BSA		Unclear, might depend on reaction volume and/or cell type
1 mg/mL BSA + 0.2% Triton		No benefit vs Triton alone
250 mM GuHCl		Might be beneficial, skip RNAse inhibitor / nuclear lysis (?)
FS oligodT - 2 uM1		Works for large cells but might not be necessary
FS oligodT - 0.4 uM (1)		Optimal condition for large and small cells
FS oligodT - 0.2 uM (1)		Works for small cells only
FS oligodT - 0.04 uM (1)		Insufficient even for small cells
dNTP conc - 0.3 mM (5)		Significant reduction in the number of detected genes
dNTP conc - 1.5 mM (5)		Optimal condition
**Lysis & mRNA denaturation**		
72°C - 3 min		Optimal condition
72°C - 10 min		No benefit or slightly deleterious
95°C - 3 min		May favor rna degradation
**RT-PCR reaction**		
RT 50°C - 60 min		Optimal condition for Superscript IV or Maxima H-
RT 37°C - 60 min		Increases the amount of multi-mapped reads
Extension step pre-ampl PCR - 4 min		Lower number of detected genes
Extension step pre-ampl PCR - 6 min		Optimal condition

Note:
If not clearly stated, concentration of reagents and reaction conditions are the same as in the standard FLASH-seq protocol.

Table E1: Summary of all conditions tested in this study.

Condition tested	Effect	Comment
**RT-PCR Mix**		
Addition of 20 mM NaCl (2)		No benefit
Addition of 30 mM NaCl (2)		No benefit
Addition of 40 mM NaCl (2)		Inhibits the reaction
Addition of 1 mM GTP / no dCTP		Decreases reaction efficiency
Superscript IV in RT		Optimal enzyme
Maxima H- in RT		Optimal enzyme
FS TSO - 2 uM (3)		Optimal condition for most applications
FS TSO - 3 uM (3)		Performs just slightly worse than 2 uM
FS TSO - 4 uM (3)		Increases the amount of multi-mapped reads
FS TSO - 6 uM (3)		Increases the amount of multi-mapped reads
FS TSO - 8 uM (3)		Increases the amount of multi-mapped reads
FS TSO - 10 uM (3)		Increases the amount of multi-mapped reads.
Ficoll-400 - 4% v/v (4)		No benefit
Betaine - 1M final		Optimal condition
Betaine - complete removal		Significantly worse performance
dCTP - no extra dCTP added		Works fine but not optimal
dCTP - 1.65 mM dCTP extra		Works fine but not optimal
dCTP - 3.3 mM dCTP extra		Optimal condition
dCTP - 5.5 mM dCTP extra		Works very well but can slightly decreases the uniquely mapped reads
dCTP - 8.25 mM dCTP extra		Increases the amount of multi-mapped and intergenic reads
dCTP - 11.1 mM dCTP extra		Increases the amount of multi-mapped and intergenic reads
Pfu DNA Pol - 0.25 U/rxn		No benefit
Pfu DNA Pol - 0.375 U/rxn		No benefit
ET SSB - 40 ng		No benefit
ET SSB - 80 ng		No benefit
ET SSB - 160 ng		No benefit
ET SSB - 250 ng		Inhibits the reaction
T4g32 protein - 5 ug + RT at 50°C - 25 ul rxn		No benefit
T4g32 protein - 5 ug - 25 ul rxn		Increases the amount of multi-mapped and intergenic reads
T4g32 protein - 0.125 ug - 5 ul rxn		No benefit
T4g32 protein - 0.25 ug - 5 ul rxn		No benefit
T4g32 protein - 0.50 ug - 5 ul rxn		No benefit
T4g32 protein - 0.75 ug - 5 ul rxn		No benefit
T4g32 protein - 1 ug - 5 ul rxn		Increases the amount of unmapped reads
T4g32 protein - 0.25 ug + 0.4 uM FS oligodT1 - 5 ul rxn		No benefit
T4g32 protein - 0.25 ug + 0.02 uM FS oligodT1 + 5 ul rxn		No benefit
T4g32 protein - 0.25 ug + Maxima - 5 ul rxn		No benefit
T4g32 protein - 0.50 ug + Maxima - 5 ul rxn		No benefit
T4g32 protein - 0.75 ug + Maxima - 5 ul rxn		No benefit
T4g32 protein - 0.25 ug - 5 ul rxn - RT 30 min		Reduction in the number of detected genes
T4g32 protein - 0.50 ug - 5 ul rxn - RT 30 min		Reduction in the number of detected genes
T4g32 protein - 0.75 ug - 5 ul rxn - RT 30 min		Reduction in the number of detected genes
T4g32 protein - 0.25 ug - 5 ul rxn - 1.2% Triton (lysis)		Reduction in the number of detected genes
T4g32 protein - 0.5 ug - 5 ul rxn - 1.2% Triton (lysis)		Reduction in the number of detected genes
T4g32 protein - 1 ug - 5 ul rxn - 1.2% Triton (lysis)		Reduction in the number of detected genes
T4g32 protein - 0.25 ug - 5 ul rxn - 4 uM FS TSO3		Increases the amount of multi-mapped and intergenic reads
T4g32 protein - 0.25 ug - 5 ul rxn - 6 uM FS TSO3		Increases the amount of multi-mapped and intergenic reads
T4g32 protein - 0.25 ug - 5 ul rxn - 8 uM FS TSO3		Increases the amount of multi-mapped and intergenic reads
T4g32 protein (home-made) - 5 ug - 25 ul rxn		Increases the amount of multi-mapped and intergenic reads

Note:
If not clearly stated, concentration of reagents and reaction conditions are the same as in the standard FLASH-seq protocol.
[1] Concentration in the Lysis Mix;
[2] As FLASH-seq is performed in the KAPA HiFi mix and not in a standard RT mix, we could only add NaCl on top and not instead of the reaction buffer.
;
[3] Even if the TSO is added with the Lysis Mix, the value reported here refers to the final concentration in the RT-PCR reaction. ;
[4] Due to volume constraints, Ficoll addition required the removal of betaine from the RT-PCR reaction mix. ;
[5] KAPA HiFi HotStart readyMix contains 0.3 mM dNTPs. FS Lysis Mix contains 1.2 mM dNTPs. ;

Cell lysis step

An ideal lysis buffer should break the cell membrane and free the mRNA content without damaging it or affecting the downstream reactions.

In this study we tested the following conditions:

Triton X-100, 1.2% [hPBMCs-5µl], Fig E1: increasing the Triton X-100 concentration in the lysis buffer resulted in an increased percentage of multi-mapped reads.

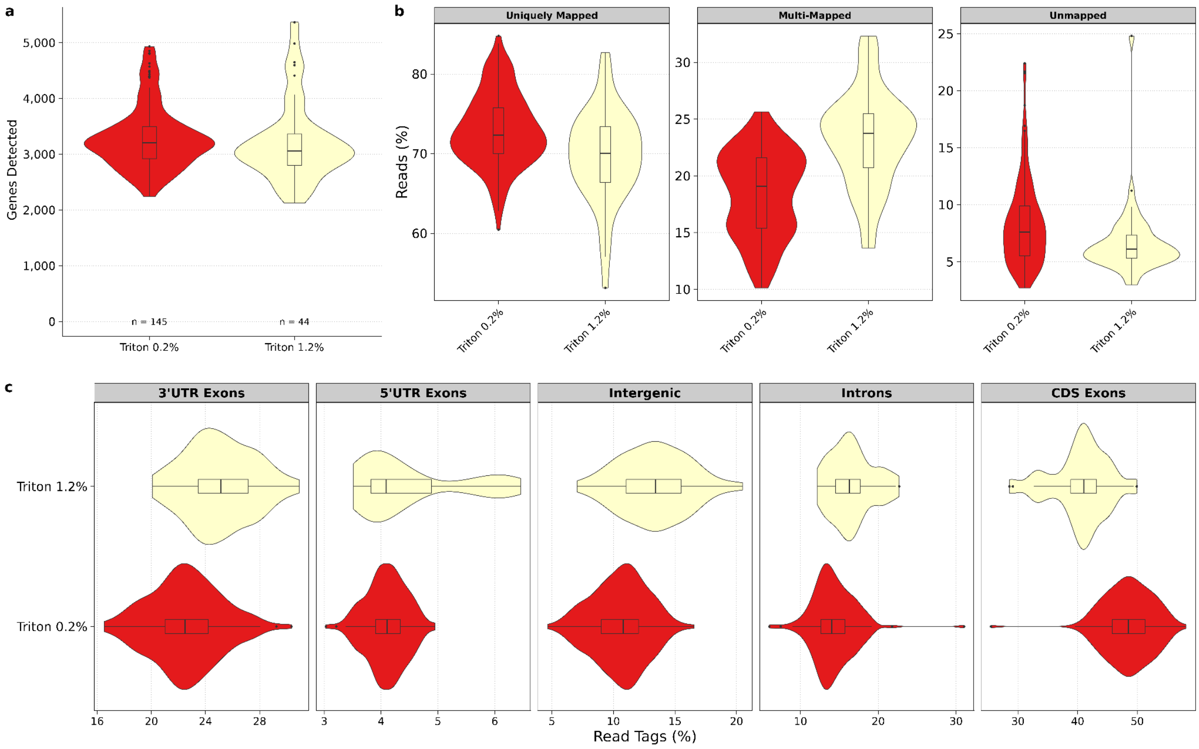

Fig E1 | hPBMCs - 5µl - Triton X-100, 1.2% a. Number of genes detected. No significant difference was observed between 0.2% (*n*=145) and 1.2% Triton X-100 (*n*=44) (Wilcoxon rank sum test, two-sided). **b.** STAR mapping statistics showing the percentage of uniquely mapped, multi-mapped and unmapped reads. **c.** Distribution of mapped reads between introns, intergenic regions or 3'-UTR / 5'-UTR / coding sequence (=CDS) exons. Expressed in percentage of read tags and computed using ReSQC.

Bovine serum albumin (BSA, 1 mg/ml) [HEK-25µl / hPBMCs-5µl], Fig E2, E3: BSA is a long-known PCR additive used to facilitate the reaction when inhibitors are present[1]. It also displays macromolecular crowding properties[2] and can lyse cells if used at high concentration[3]. Replacing Triton X-100 with BSA (1 mg/ml final concentration in lysis buffer) had different effects in our two cell-type models. In HEK 293T cells (25 µl), BSA increased the number of genes detected. However, BSA failed to provide the same benefit in hPBMCs (5 µl). We hypothesize that its macromolecular crowding properties may play a role when the reaction is carried out in larger reaction volumes, but becomes negligible in smaller volumes. Of note, BSA proved difficult to be dispensed on plates with the I.DOT, making it less user-friendly than Triton X-100. Combining BSA with Triton (1 mg/ml BSA + 0.2% Triton X-100, final concentration in lysis) did not improve the number of detected genes in hPBMCs.

As our main purpose was to develop a reliable and easy-to-implement protocol, we decided not to use BSA. More tests are required to determine whether BSA can offer an advantage in particular reaction settings.

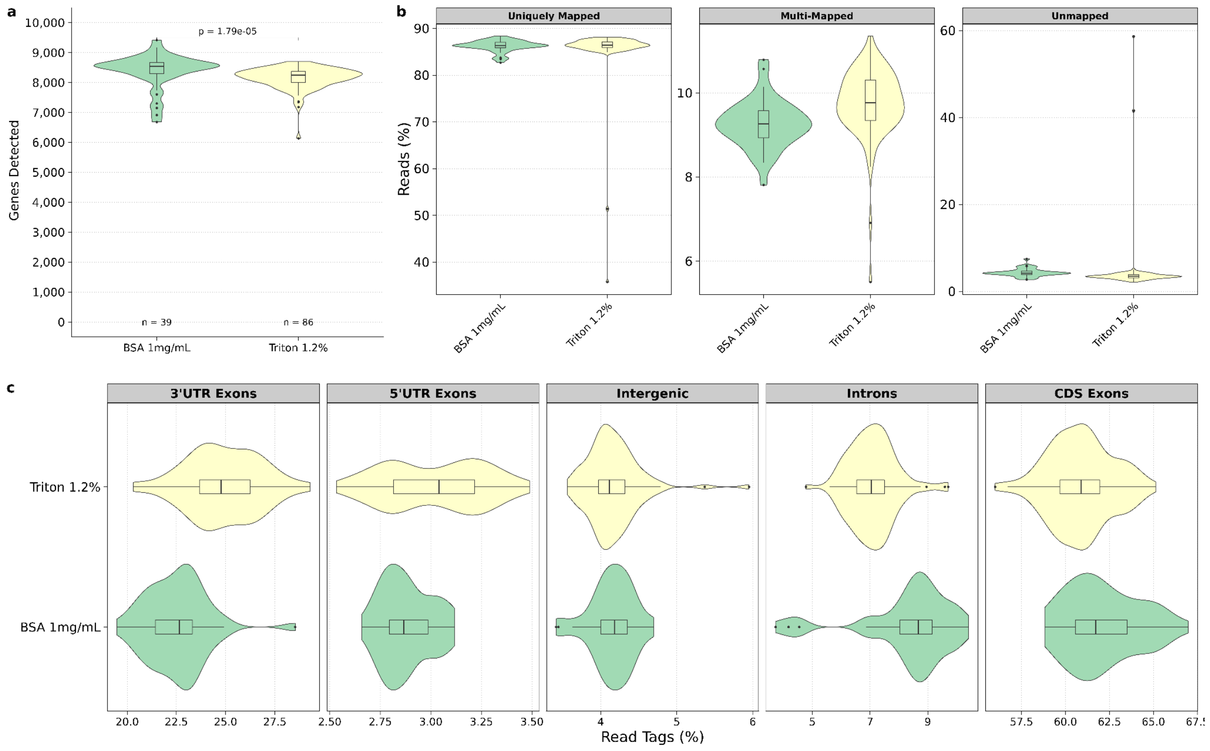

Fig E2 | HEK 293T - 25µl - Bovine serum albumin (BSA, 1 mg/ml) a. Number of genes detected in cells lysed with 1 mg/ml BSA (*n*=39) or triton 1.2% (*n*=86) (Wilcoxon rank sum test, two-sided, *P*-value). **b.** STAR mapping statistics showing the percentage of uniquely mapped, multi-mapped and unmapped reads. **c.** Distribution of mapped reads between introns, intergenic regions or 3'-UTR / 5'-UTR / coding sequence (=CDS) exons. Expressed in percentage of read tags and computed using ReSQC.

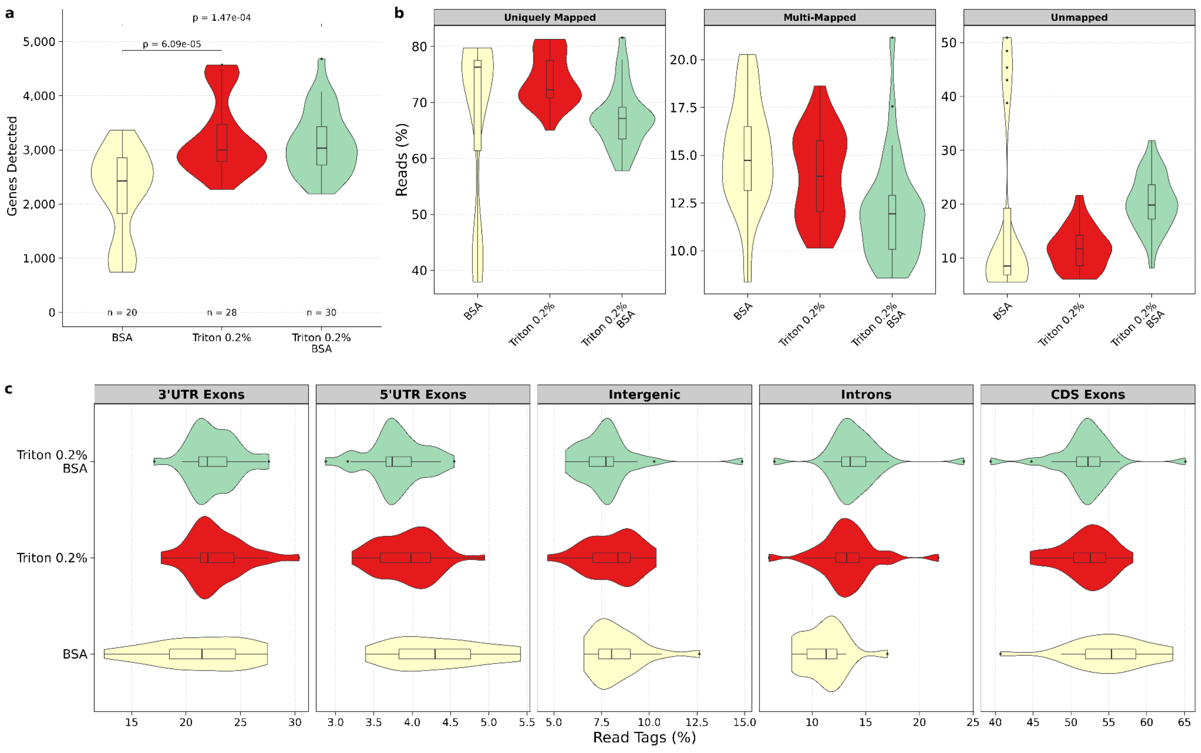

Fig. E3 | hPBMCs - 25 µl - Bovine serum albumin (BSA, 1 mg/ml) a. Number of detected genes in cells lysed with 1 mg / ml BSA (*n*=20), 0.2% triton (*n*=28) or a mixture of 1 mg/ml BSA and 0.2% triton (*n*=30) (Dunn's test, two-sided, Bonferroni correction, adj. *P*-value). **b.** STAR mapping statistics showing the percentage of uniquely mapped, multi-mapped and unmapped reads. **c.** Distribution of mapped reads between introns, intergenic regions or 3'-UTR / 5'-UTR / coding sequence (=CDS) exons. Expressed in percentage of read tags and computed using ReSQC.

Guanidine hydrochloride (GuHCl, 250 mM, 250K downsampled reads) [HEK-5µl], Fig E4: GuHCl is a chaotropic agent commonly used in DNA/RNA extraction protocols (i.e., Qiagen DNA/RNA extraction kit). Its strong denaturing properties are sufficient to inhibit nucleases, therefore making the addition of RNAse inhibitors in the lysis buffer superfluous, as indirectly confirmed by the average size of pre-amplified cDNA (Fig E4a, right panel). GuHCl could be particularly useful when working with cells prone to RNA degradation or which resist Triton X-100 lysis.

Replacing Triton X-100 with 250 mM GuHCl in the lysis buffer (= 50 mM in the final RT-PCR reaction) slightly improved the number of genes being detected.

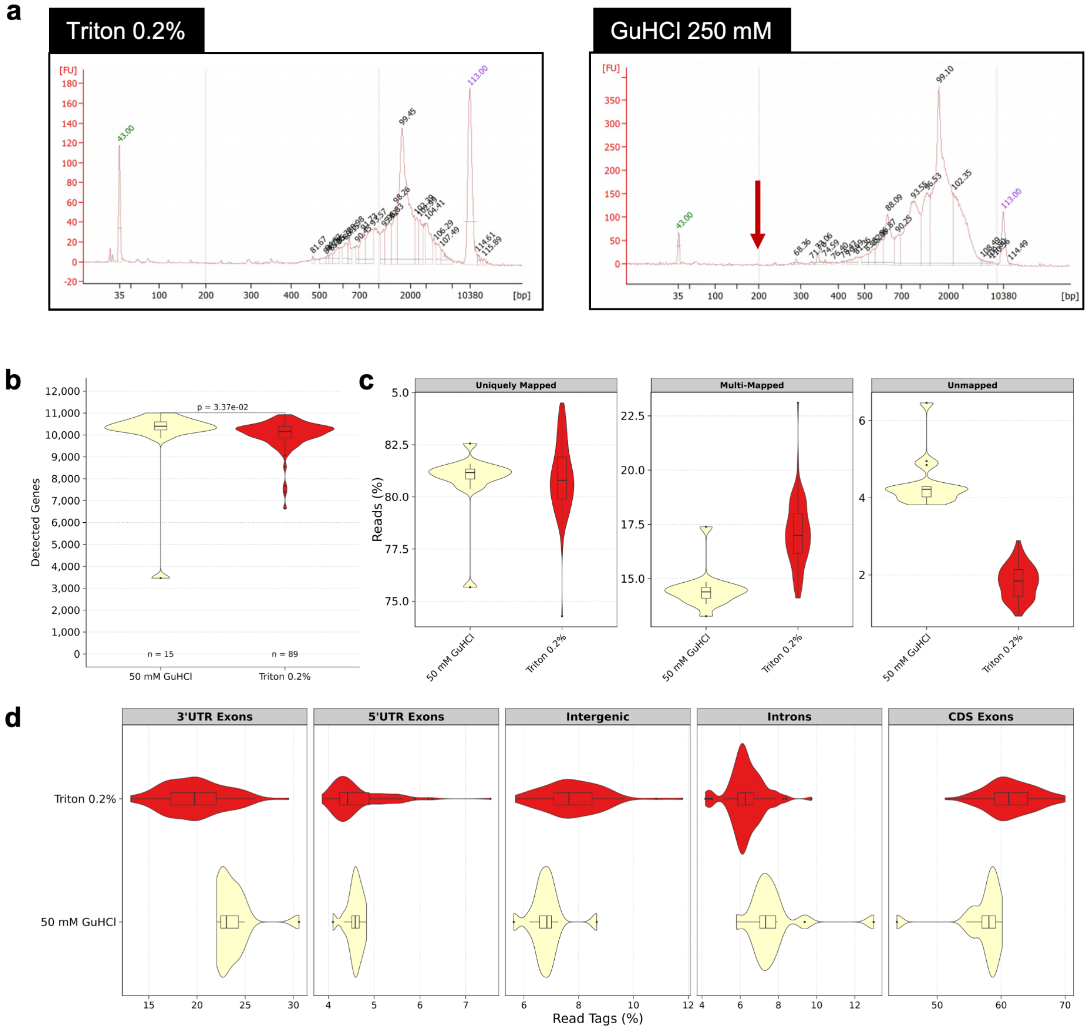

Fig E4 | HEK - 5µl - Guanidine hydrochloride (GuHCl, 250 mM), 250K raw reads a. Bioanalyzer traces of two selected cells lysed with either 0.2% Triton X-100 (left) or 250 mM GuHCl (right). The arrow highlights the absence of RNA degradation (fragments <400bp) despite the RNAse inhibitor not being present. **b.** Number of genes detected in cells processed with a lysis buffer containing 250 mM GuHCl (*n*=15) or 0.2% Triton (*n*=89). Final concentration of GuHCl in the RT reaction is 50 mM. No significant difference was observed (Wilcoxon rank sum test, two-sided, *P*-value). **c.** STAR mapping statistics showing the percentage of uniquely mapped, multi-mapped and unmapped reads. **d.**

Distribution of mapped reads between introns, intergenic regions or 3’-UTR / 5’-UTR / coding sequence (=CDS) exons. Expressed in percentage of read tags and computed using ReSQC.

Denaturation step

A denaturation step is performed prior to reverse transcription, to resolve RNA secondary structures. We tested different denaturation conditions (duration and temperature) with the aim of improving cell lysis and mRNA recovery rates.

3 vs 10 min RNA denaturation (72°C) [hPBMCs-5µl], Fig E5: increasing the denaturation time from 3 to 10 min, as reported in other protocols[4], did not affect the number of genes detected. We observed, however, a small increase in the proportion of intronic reads.

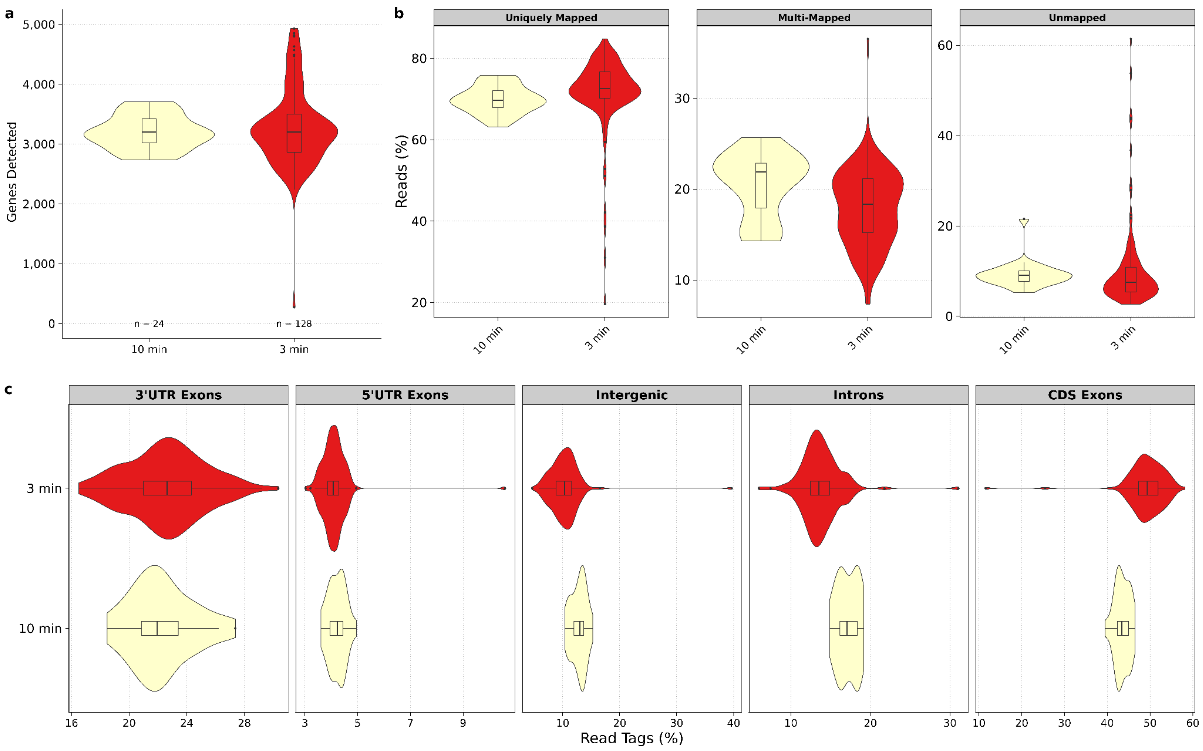

Fig E5 | hPBMCs - 5µl - RNA denaturation timing a. Number of genes detected in cells that underwent a 3 (n=128) or 10 (n=24) minutes RNA denaturation. No significant difference was observed (Mann-Whitney U test, two-sided, P-value). **b.** STAR mapping statistics showing the percentage of uniquely mapped, multi-mapped and unmapped reads. **c.** Distribution of mapped reads between introns, intergenic regions or 3’-UTR / 5’-UTR / coding sequence (=CDS) exons. Expressed in percentage of read tags and computed using ReSQC.

Denaturation temperature (72°C vs 95°C) [hPBMCs-5µl, 250K downsampled reads], Fig E6: the denaturation step is performed at 72°C in most scRNA-seq protocols. In the STRT-seq-2i publication the authors used 95°C instead, reporting higher cDNA yield and longer average cDNA length[5]. In our study, carrying out the denaturation at 95°C resulted in a higher proportion of multi-mapped reads, a 3'-end gene body coverage bias, a lower average number of genes detected and a striking increase in the proportion of intronic *vs* exonic features, compared to the standard 72°C denaturation (Fig E6). However, when assessing the number of expressed genes using both exonic and intronic features, 95°C denaturation showed better gene detection (Fig E6c). Among protein-coding genes, 95°C denaturation resulted in 2.38-times more genes with only intronic and no exonic reads ($\mu_{onlyIntronics\_72°C}$=429 and $\mu_{onlyIntronics\_95°C}$=1022).

The origin of these intronic reads is not yet clear. While we cannot exclude that they are the result of a better denaturation of mRNAs with strong secondary structures or a more complete nuclear lysis , we hypothesize that higher temperatures might result in a mild fragmentation of the mRNA. This is also supported by the observed 3'-end gene body coverage bias and greater proportion of intergenic reads. Due to these conflicting observations, we kept using the standard 72°C denaturation, as RNA fragmentation may significantly bias differential expression, isoform or any UMI-based analysis.

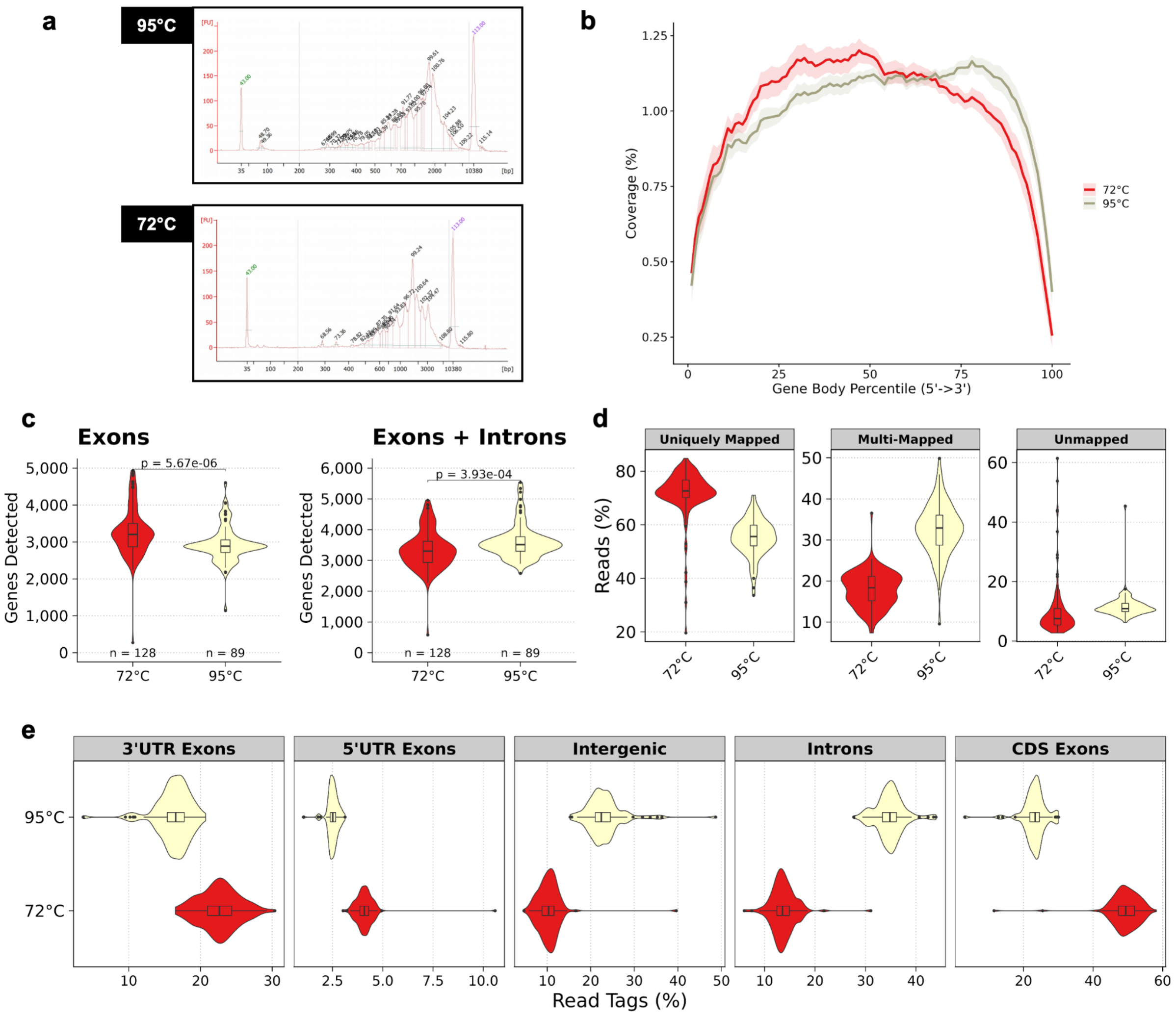

Fig E6 | RNA denaturation temperature [hPBMCs - 5µl] a. Bioanalyzer traces of two selected cells where the denaturation step was carried out at 95°C (top) or 72°C (bottom) for 3 min. **b.** Mean±(SD) gene body coverage percentage. **c.** Number of genes detected in cells that underwent a denaturation at 72°C (*n*=128) or at 95°C (*n*=89) for 3 minutes, using exonic (left) or exonic+intronic (right) reads (Wilcoxon rank sum test, two-sided, *P*-value). **d.** STAR mapping statistics showing the percentage of uniquely mapped, multi-mapped and unmapped reads. **e.** Distribution of mapped reads between introns, intergenic regions or 3'-UTR / 5'-UTR / coding sequence (=CDS) exons. Expressed in percentage of read tags and computed using ReSQC.

Reverse transcription

5-, 10-, 50-times lower FS SMART-dT$_{30}$VN oligonucleotide [hPBMCs-5µl], Fig E7: in hPBMCs, we observed large amounts of leftover primer dimers after pre-amplification which could not be completely removed, even with a very stringent magnetic bead cleanup. As reported in the literature, their length make them a potential substrate for the Tn5 transposase and care should be therefore taken in minimizing their presence [6,7] (Fig E7a). FS SMART-dT$_{30}$VN is present in the lysis buffer at a concentration of almost 10 µM. Upon addition of the RT-PCR mix (4:1 volume ratio RT-PCR mix:lysis buffer) the concentration decreases to 2 µM. Reducing the amount of FS SMART-dT$_{30}$VN by 5- or 10-times (0.4 / 0.2 µM final) eliminated the primer dimer peaks in hPBMCs, while preserving similar cDNA yields and gene detection (Fig E7b-c). A 50-times reduction in concentration (0.04 µM final) significantly reduced the cDNA yield and was therefore not pursued further. As larger cells may require higher amounts of oligos we decided to use the 0.4 µM final concentration (i.e., 5-times reduction) as standard condition in all the following experiments.

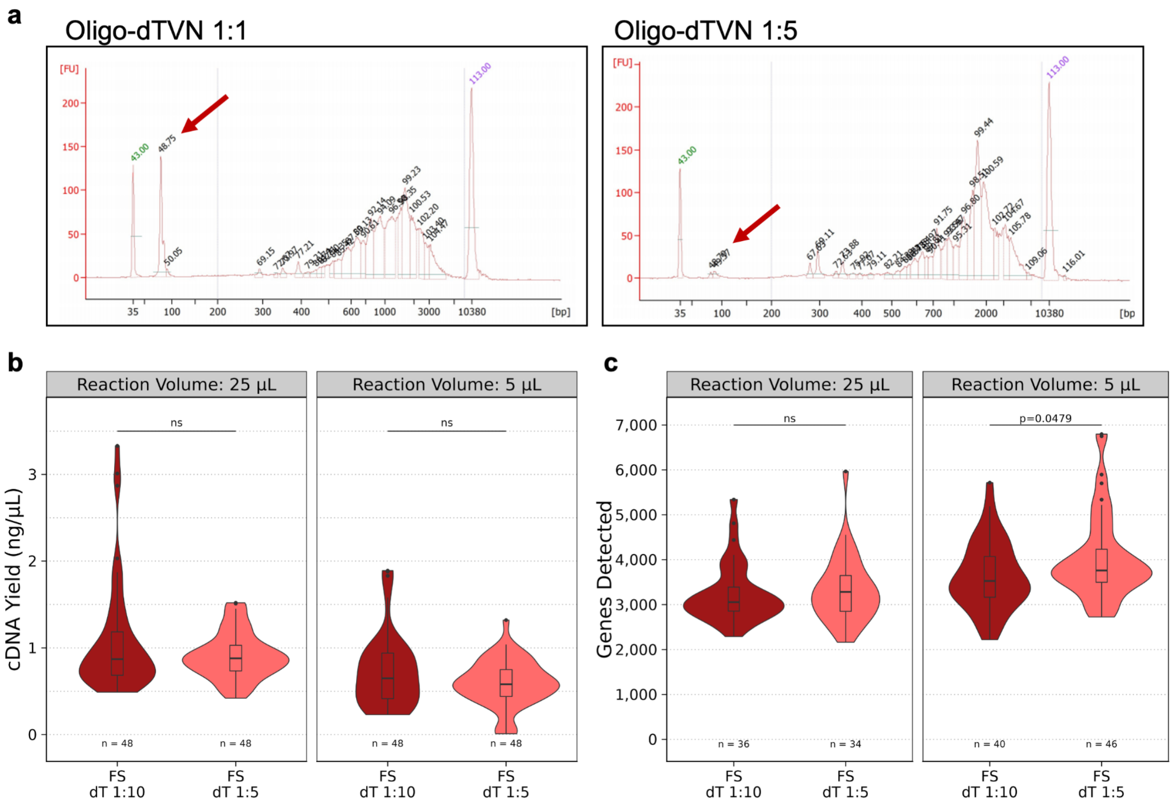

Fig E7 | hPBMCs - 5µl or 25µl- FS SMART-dT$_{30}$VN oligo concentration a. cDNA size distribution of two selected cells processed with the full amount (left) or a 5-times reduction (right) of FS SMART-dT$_{30}$VN oligo. The red arrow indicates the primer dimer peak. **b.** cDNA yields using 5- (*n*=48) or 10-times less (*n*=48) FS SMART-dT$_{30}$VN in a 25 µl or 5 µl reaction (Wilcoxon rank sum test, two-sided, *P*-value). **c.** Number of detected genes using 5- or 10-times less FS SMART-dT$_{30}$VN in a 25 µl or 5 µl reaction ($n_{FS\_25\mu L\_1:10dT}$=36, $n_{FS\_25\mu L\_1:5dT}$=34, $n_{FS\_5\mu L\_1:10dT}$=40, $n_{FS\_5\mu L\_1:5dT}$=46, Wilcoxon rank sum test, two-sided, *P*-value).

0, 20, 30, 40 mM NaCl [hPBMCs-5µl], Fig E8: NaCl has been shown to improve the performance of Maxima H- reverse transcriptase in the SS3 protocol[4]. Adding 20 or 30 mM NaCl to the FS RT-PCR mix containing Superscript™ IV did not increase the number of genes detected. Forty millimolar NaCl inhibited the reaction completely and these samples could therefore not be sequenced.

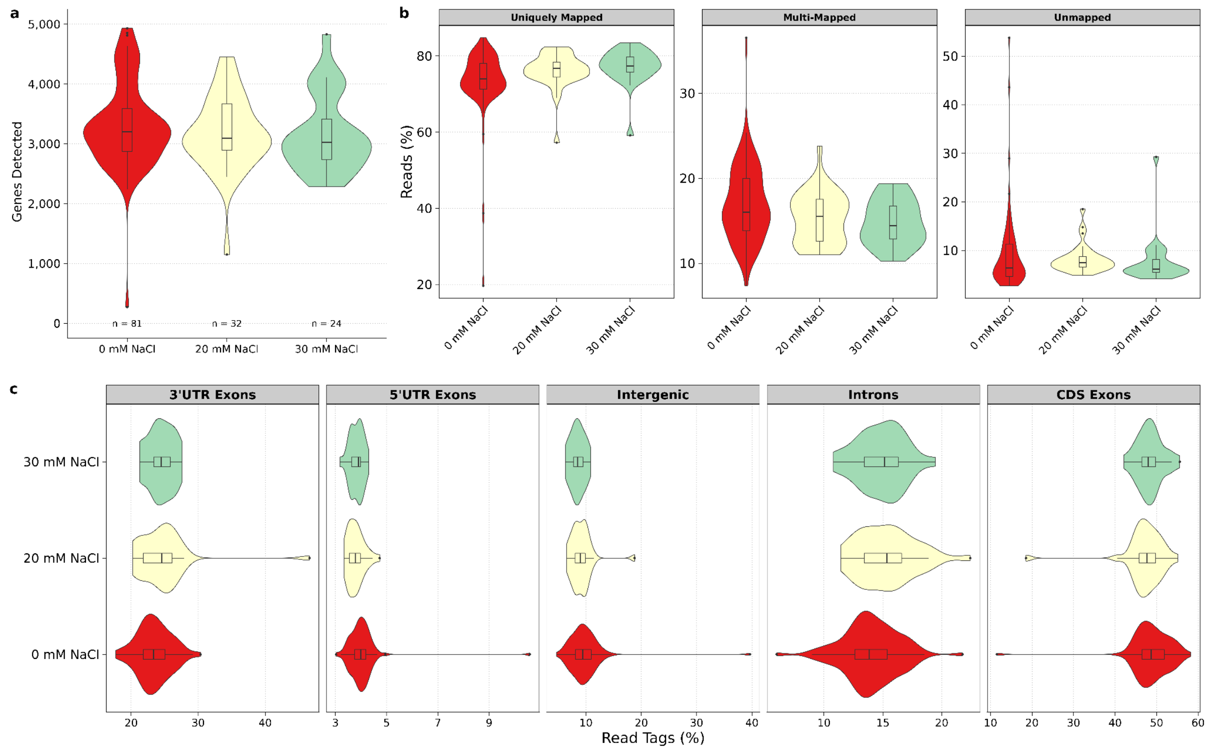

Fig E8 | hPBMCs - 25µl - NaCl in RT-PCR a. Number of genes detected in cells processed with 0 (*n*=81), 20 (*n*=32) or 30 (*n*=24) mM NaCl (Dunn's test, two-sided, Bonferroni correction, adj. *P*-value). **b.** STAR mapping statistics showing the percentage of uniquely mapped, multi-mapped and unmapped reads. **c.** Distribution of mapped reads between introns, intergenic regions or 3'-UTR / 5'-UTR / coding sequence (=CDS) exons. Expressed in percentage of read tags and computed using ReSQC.

RT temperature (37°C *vs* 50°C) [hPBMCs-5µl], Fig E9: Moloney murine leukemia virus (MMLV)-derived reverse transcriptases work at a wide range of temperatures (37°C - 55°C). While higher temperatures are generally recommended for resolving RNA secondary structures, we did not observe any significant decrease in the number of genes detected when carrying out the reaction at 37°C. However, the percentage of multi-mapped reads (e.g., ribosomal reads) increased, which could indicate a lower specificity of the poly-A priming of Superscript™ IV at 37°C.

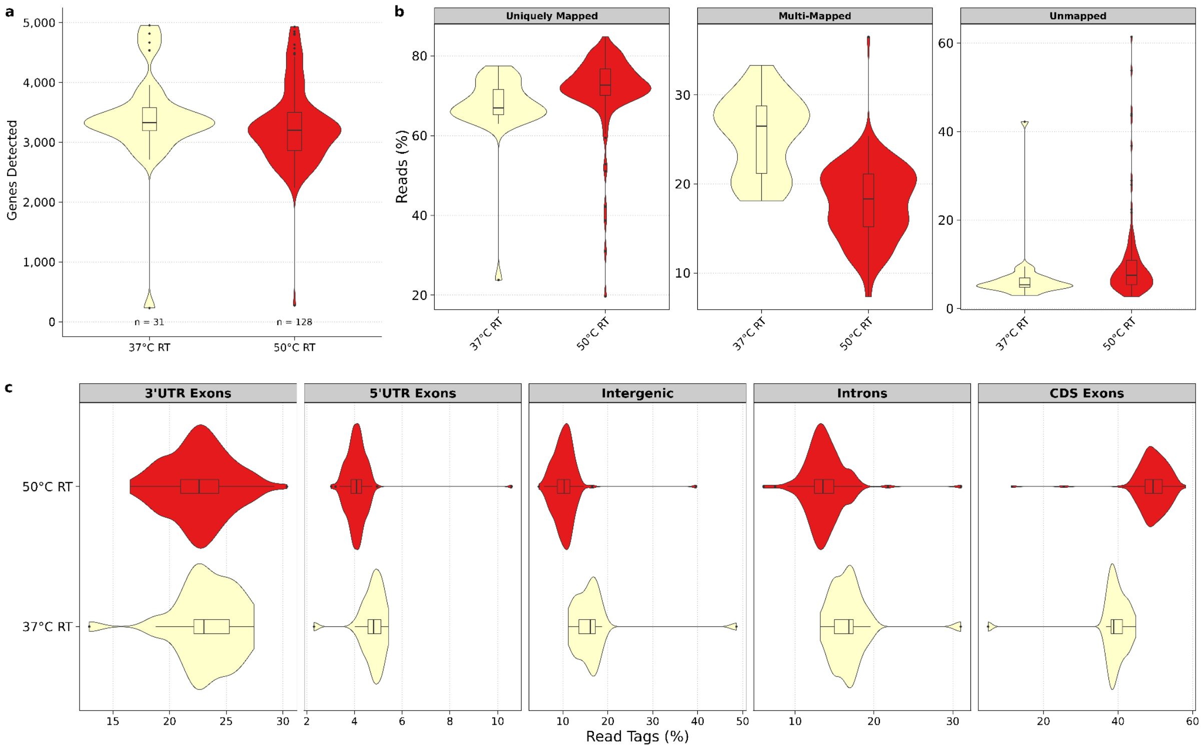

Fig E9 | hPBMCs - 5µl - RT temperature a. Number of genes detected in cells that underwent a reverse transcription at 37°C (*n*=31) or 50°C (*n*=128). No significant difference was observed between temperatures (Wilcoxon rank sum test, two-sided, *P*-value). **b.** STAR mapping statistics showing the percentage of uniquely mapped, multi-mapped and unmapped reads. **c.** Distribution of mapped reads between introns, intergenic regions or 3'-UTR / 5'-UTR / coding sequence (=CDS) exons. Expressed in percentage of read tags and computed using ReSQC.

RT enzyme (Superscript™ IV *vs* Maxima H-) [hPBMCs-5µl], Fig E10: two of the most widely used reverse transcriptases in scRNA-seq are Superscript™ IV and Maxima H-. Both perform well in the FS reaction buffer without any significant difference in gene detection.

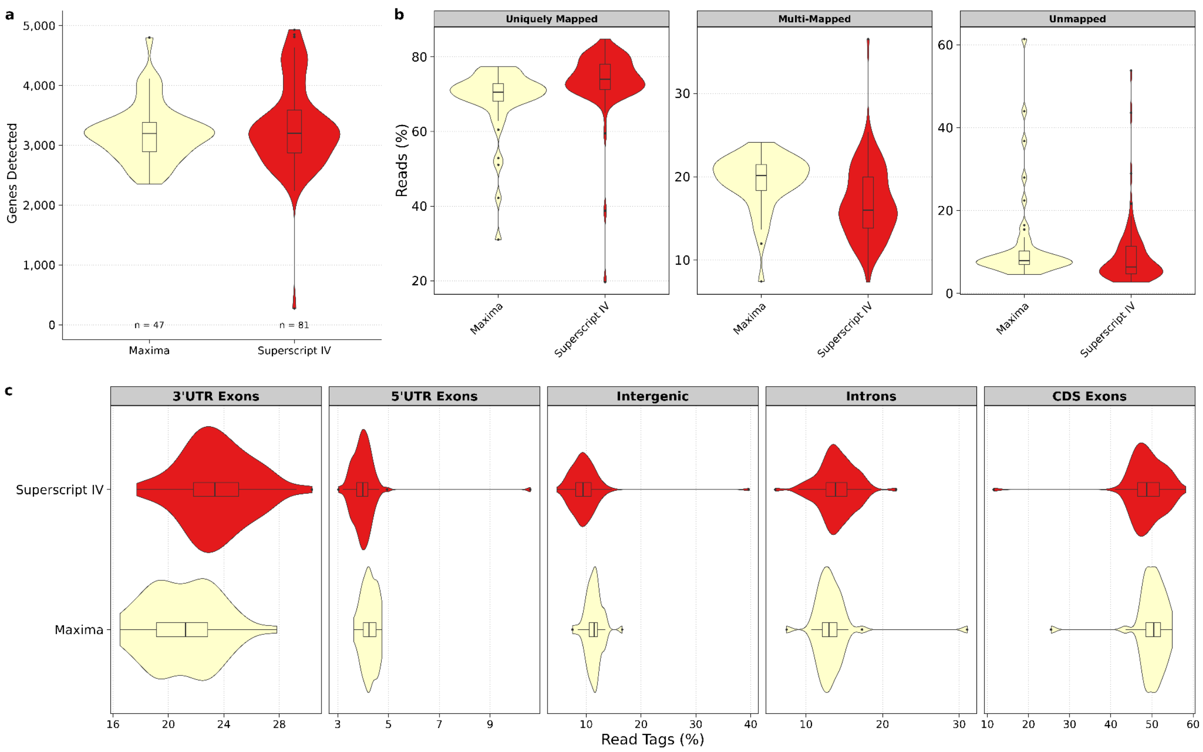

Fig E10 | hPBMCs - 5µl - RT enzyme a. Number of genes detected in cells that underwent a reverse transcription with Maxima H- (*n*=47) or Superscript IV (*n*=81). No significant difference was observed (Wilcoxon rank sum test, two-sided, *P*-value). **b.** STAR mapping statistics showing the percentage of uniquely mapped, multi-mapped and unmapped reads. **c.** Distribution of mapped reads between introns, intergenic regions or 3'-UTR / 5'-UTR / coding sequence (=CDS) exons. Expressed in percentage of read tags and computed using ReSQC.

Template-switching oligonucleotide (TSOs) [hPBMCs-5µl, 250K downsampled raw reads] Fig E11: several MMLV-based reverse transcriptases have been shown to exhibit a template-switching activity. Upon reaching the 5´-end of the mRNA molecule, these enzymes add a short stretch of untemplated nucleotides (generally CCC) at the 5'-end of the newly synthesized cDNA molecule. These nucleotides are then used as an anchor to anneal the TSO. The enzymes are then able to switch template: while they initially use the mRNA as template to generate a complementary DNA molecule, they later use the DNA-based TSO to generate an additional portion of cDNA, covalently linked to the cDNA derived from the mRNA. In the end, all cDNA molecules carry a known sequence at both the 5'- and 3´-end [8]. However, the very short and variable length as well as the varying base composition of these untemplated nucleotides makes the annealing very inefficient[9] and a large excess of TSO is usually required to ensure a high conversion rate to cDNA[10].

Similarly to SS2, FS is a highly robust protocol that can accommodate some variations in concentration of all its reagents with negligible efficiency losses. The only exception is represented by large shifts in TSO concentration. For this reason, we titrated the amount of TSO required to efficiently process hPBMCs, by evaluating gene detection when using 1-, 1.5-, 2-, 3-, 4- or 5-times more TSO, with 1 x TSO corresponding to a final concentration of about 2 µM in the final RT-PCR reaction. We observed a linear increase in the number of genes detected with increasing TSO concentrations (median increase = 0 - 8.74%). However, this improvement was accompanied by a significantly higher proportion of multi-mapped reads at the expense of exonic features. The increase in intergenic and multi-mapped reads generated a cumulated excess of up to ~17% of unusable raw reads (4 x TSO, about 8 µM). In addition, the increased percentage of intergenic reads may indicate some level of strand-invasion (the reader is referred to the main manuscript for further details about the strand invasion phenomenon). We therefore decided not to investigate this aspect further. Of note, larger cells (i.e., HEK 293T, not tested in these settings) may lead to different conclusions.

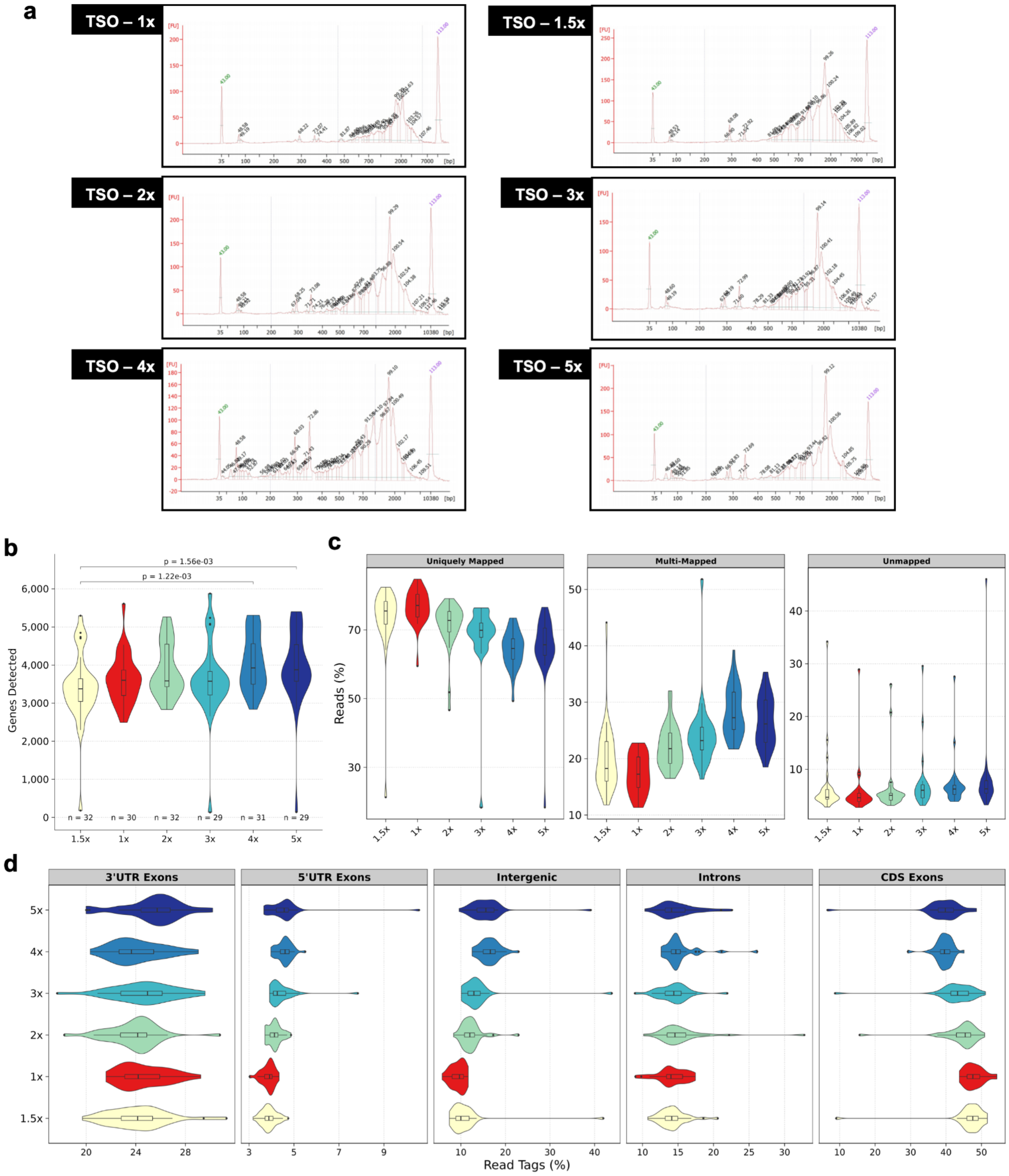

Fig E11 | hPBMCs - 5µl - TSO titration a. Bioanalyzer traces of selected cells processed with different amounts of TSO. **b.** Number of genes detected in cells processed with 1- (*n*=30), 1.5- (*n*=32), 2- (*n*=32), 3- (*n*=29), 4- (*n*=31) or 5-times (*n*=29) the standard amount of TSO (Dunn's test, two-sided, Bonferroni correction, adj. *P*-value). **c.** STAR mapping statistics showing the percentage of uniquely mapped, multi-mapped and unmapped reads. **d.** Distribution of mapped reads between introns, intergenic regions or 3'-UTR / 5'-UTR / coding sequence (=CDS) exons. Expressed in percentage of read tags and computed using ReSQC.

Ficoll-400, 4% w/v [hPBMCs-5µl], Fig E12: Ficoll-400 is a highly branched molecule formed by the copolymerization of sucrose and epichlorohydrin, known for its macromolecular crowding properties[2]. In our settings, the addition of Ficoll-400 to a final concentration of 4% w/v did not improve the reaction. However, we have to make the reader aware that betaine (generally present in the RT-PCR mix at 1 M final concentration) had to be removed in order to use Ficoll-400, due to volume constraints. Betaine is one of the key additives in the SS2 protocol[10]. These results suggest that the effects of Ficoll-400 could compensate for the betaine removal (see also Fig E13).

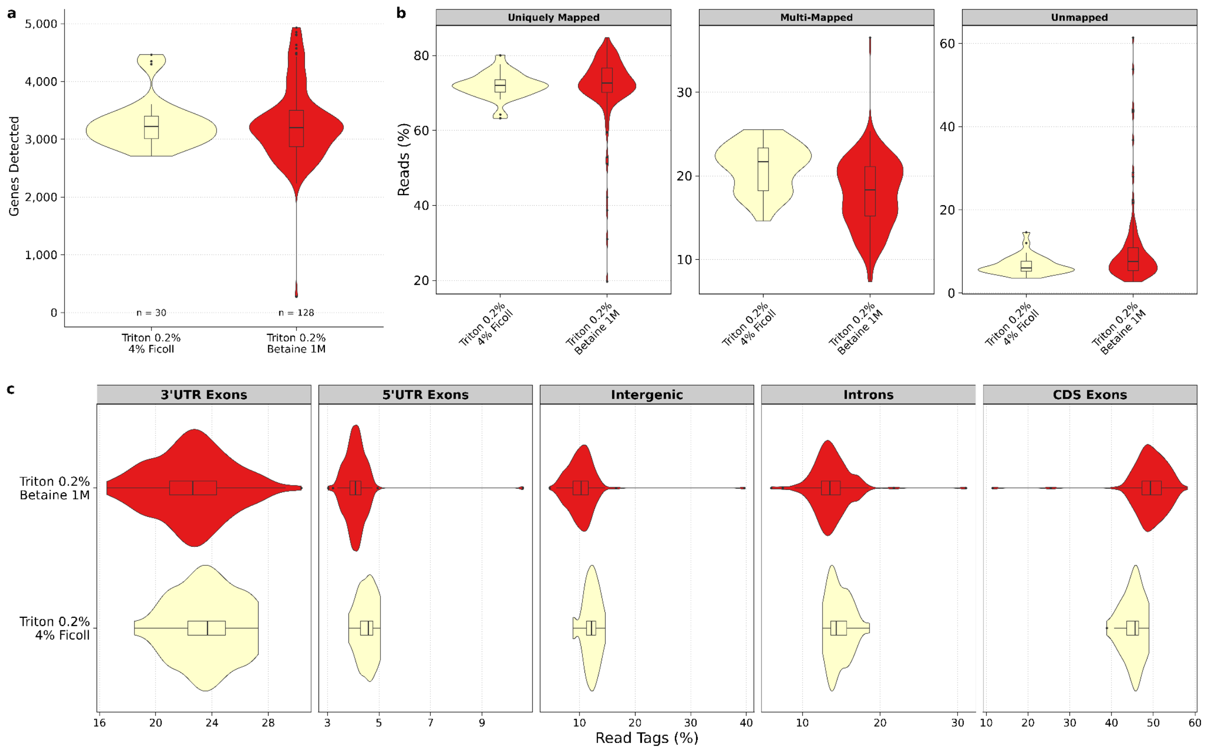

Fig E12 | hPBMCs - 5µl - Ficoll-400 4% w/v a. Number of genes detected in cells in a RT-PCR buffer containing 4% w/v Ficoll and no betaine (*n*=30) or 1 M betaine (*n*=128). No significant difference was observed (Wilcoxon rank sum test, two-sided, *P*-value). **b.** STAR mapping statistics showing the percentage of uniquely mapped, multi-mapped and unmapped reads. **c.** Distribution of mapped reads between introns, intergenic regions or 3'-UTR / 5'-UTR / coding sequence (=CDS) exons. Expressed in percentage of read tags and computed using ReSQC.

Betaine (1 M vs 0 M betaine) [HEK-5µl], Fig E13: betaine (N,N,N-trimethylglycine) is a methyl group donor which increases RT efficiency, mitigates RNA refolding during RT and acts as a cryoprotectant[10]. The addition of 1 M betaine was one of the key steps introduced by SS2[10]. As described above, we did not observe a significant difference in the number of genes detected when performing FS with 4% w/v Ficoll-400 without betaine (Fig E12) and the 1 M betaine control. These results suggested that either the addition of 4% w/v Ficoll-400 could compensate for the loss of betaine or that betaine is no longer needed in the FS reaction. To test these hypotheses we processed HEK 293T cells in a RT-PCR buffer with or without betaine. The number of genes detected decreased when betaine was omitted, showing that the protocol still benefits from its addition.

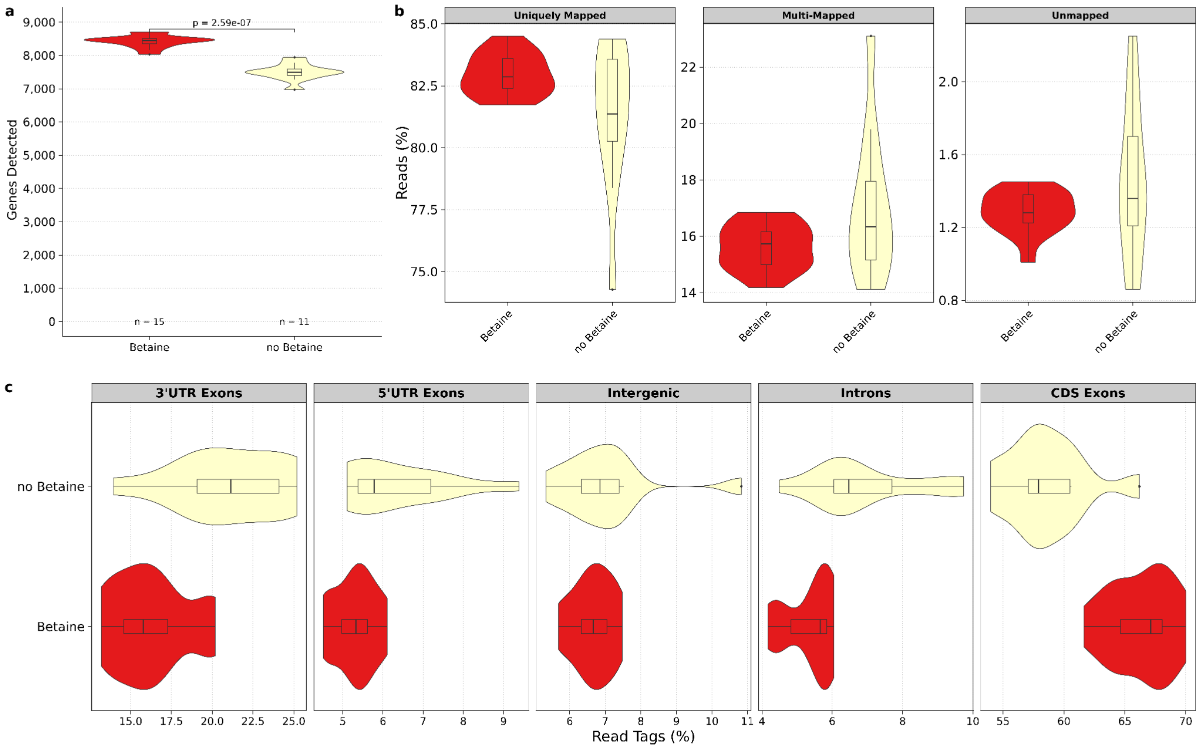

Fig E13 | HEK - 5µl - Betaine a. Number of genes detected in cells processed with 1 M (*n*=15) or no betaine (*n*=11). The addition of betaine increases the number of genes detected (Wilcoxon rank sum test, two-sided, *P*-value). **b.** STAR mapping statistics showing the percentage of uniquely mapped, multi-mapped and unmapped reads. **c.** Distribution of mapped reads between introns, intergenic regions or 3'-UTR / 5'-UTR / coding sequence (=CDS) exons. Expressed in percentage of read tags and computed using ReSQC.

dCTP (no extra dCTP, 1.65, 3.3, 5.5, 8.25 or 11.1 mM dCTP) [HEK-5µl], Fig E14: the template switching activity of the MMLV-derived reverse transcriptase results in the introduction of a short non-template nucleotide sequence at the 3'-end of the cDNA. These nucleotides are generally three cytosines[9]. To favor the addition of cytosines and therefore the efficiency of template switching[11], we increased the concentration of dCTP in the RT reaction by adding 1.65, 3.3, 5.5, 8.25 or 11.1 mM extra dCTP to the reaction (Fig E14). This significantly increased cDNA yield, indicating a more efficient template-switching reaction (Fig E14a). Interestingly, the addition of a larger excess of dCTP (11.1 mM) was counterproductive. The gene body coverage was not affected by an increase in dCTP (Fig E14b). Similarly to the trend observed for the cDNA yield, gene detection improved with the addition of extra dCTP (3.3, 5.5, 8.25 mM) (Fig E14c). However, the gain was correlated with a higher proportion of multi-mapped (Fig E14d) and intergenic (Fig E14e) reads. The addition of extra dCTP positively shifted the read GC-content closer to the average GC-content of the expressed genes compared to the "no dCTP" condition (Fig E14f). This translated into an improved detection of genes with a higher GC-content when using at least 3.3 mM dCTP (Fig E14g).

Our results do not indicate that the addition of ≤5.5 mM dCTP favors RT / PCR errors that could negatively impact the detection of new variants. First, we observed a lower percentage of mismatch per mapped base in uniquely mapped reads with the addition of dCTP (Fig E14h). Second, ≤5.5 mM dCTP had a significant but relatively minor effect on the number of substitutions detected in both lower and higher quality variants (Fig E14i). However, the addition of >5.5 mM strongly increased the number of lower quality variants. Third, the percentage of A/T -> C/G substitutions remained stable among the higher quality variants with the addition of ≤5.5 mM dCTP (Wilcoxon-rank test, two-sided, Bonferroni correction, adj. P-value > 0.5) (Fig E14j). The predominant A/T -> C/G substitutions among the lower quality variants are progressively replaced by C/G -> A/T substitutions at a concentration of >3.3 mM dCTP. We hypothesize that the intrinsic RT/PCR errors in FLASH-seq are predominantly C/G -> A/T substitutions and that they could be removed by the addition of dCTP.

These results indicate that a dCTP excess offers clear benefits in terms of cDNA yield, gene detection, lower RT-PCR errors as well as improving capture rate of GC-rich genes. However, it also illustrates the trade-offs that introducing novel additives has on the overall performance. Although our data indicate that a 5.5 mM excess of dCTP provides the highest rate of gene detection, we eventually settled on 3.3 mM (+57%) excess for two reasons. On the one hand, the difference between 3.3 and 5.5 mM extra dCTP was minimal. On the other hand, our aim was to limit the loss of uniquely mapped reads while providing a superior performance in all situations.

These results also suggest that increasing dCTP is not going to impair variant calling. However, given the complexity of calling variants with scRNA-seq, we recommend validating the newly discovered variants and/or using a whole-genome / exome reference to tell apart RT-PCR errors from real variants / mutations.

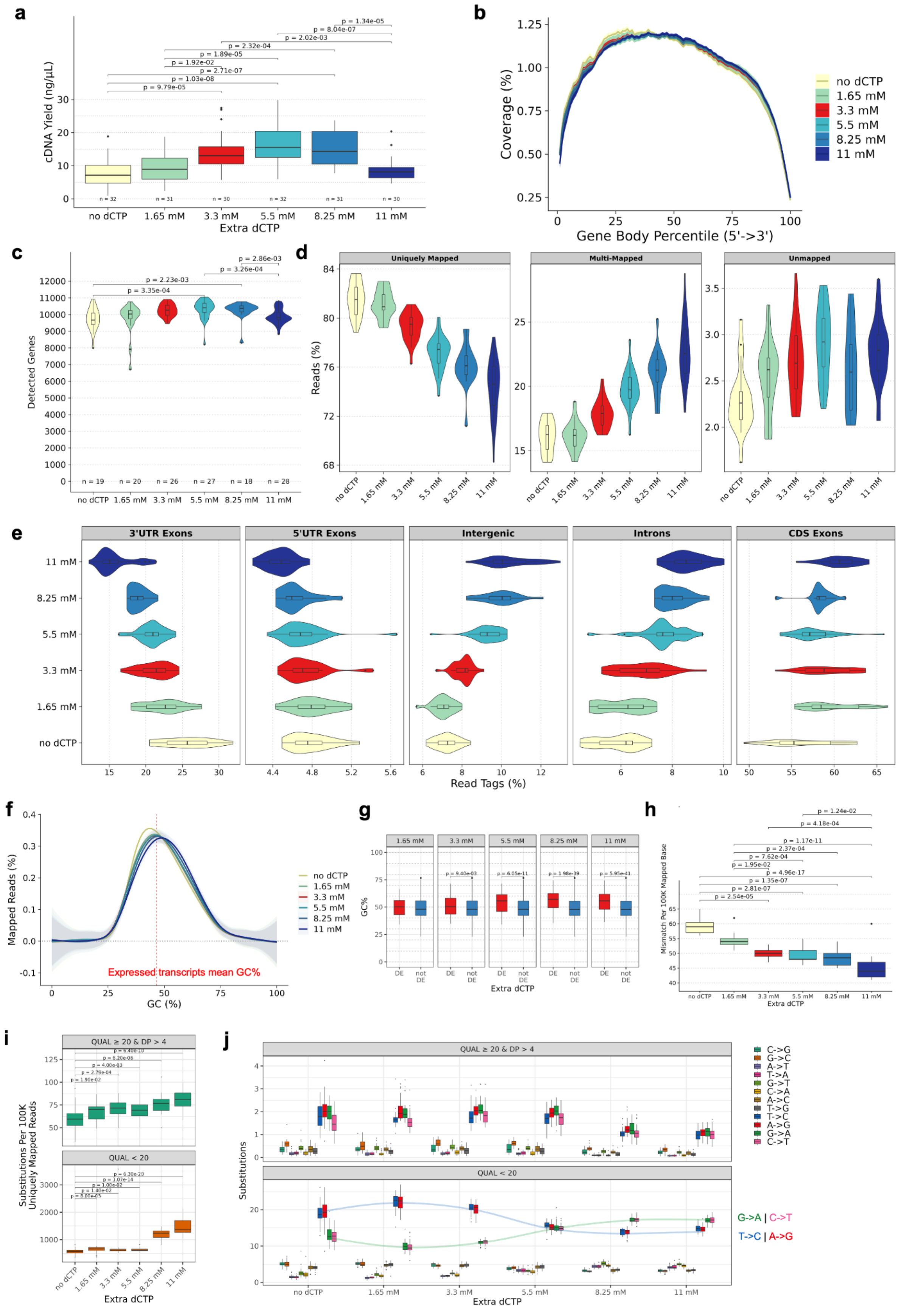
a
cDNA Yield (ng/μL)
Extra dCTP
no dCTP
1.65 mM
3.3 mM
5.5 mM
8.25 mM
11 mM
n = 32
n = 31
n = 30
n = 32
n = 31
n = 30
p = 1.34e-05
p = 8.04e-07
p = 2.02e-03
p = 2.32e-04
p = 1.89e-05
p = 1.92e-02
p = 2.71e-07
p = 1.03e-08
p = 9.79e-05
b
Coverage (%)
Gene Body Percentile (5'->3')
c
Detected Genes
n = 19
n = 20
n = 26
n = 27
n = 18
n = 28
p = 2.86e-03
p = 3.26e-04
p = 2.23e-03
p = 3.35e-04
d
Uniquely Mapped
Multi-Mapped
Unmapped
Reads (%)
e
3'UTR Exons
5'UTR Exons
Intergenic
Introns
CDS Exons
Read Tags (%)
f
Mapped Reads (%)
GC (%)
Expressed transcripts mean GC%
g
GC%
DE
not DE
p = 9.40e-03
p = 6.05e-11
p = 1.98e-39
p = 5.95e-41
h
Mismatch Per 100K Mapped Base
p = 1.24e-02
p = 4.18e-04
p = 1.17e-11
p = 2.37e-04
p = 7.62e-04
p = 1.95e-02
p = 4.96e-17
p = 1.35e-07
p = 2.81e-07
p = 2.54e-05
i
QUAL ≥ 20 & DP > 4
QUAL < 20
Substitutions Per 100K Uniquely Mapped Reads
j
Substitutions
C->G
G->C
A->T
T->A
G->T
C->A
A->C
T->G
T->C
A->G
G->A
C->T
G->A | C->T
T->C | A->G

Fig E14 | HEK - 5µl - dCTP **a.** FS cDNA yields obtained using varying amounts of dCTP (n_{0mM}=32, $n_{1.65mM}$=31, $n_{3.3mM}$=30, $n_{5.5mM}$=32, $n_{8.25mM}$=31, n_{11mM}=30) **b.** Mean±(SD) gene body coverage percentage. **c.** Number of genes detected (n_{0mM}=19, $n_{1.65mM}$=20, $n_{3.3mM}$=26, $n_{5.5mM}$=27, $n_{8.25mM}$=18, n_{11mM}=28). **d.** STAR mapping statistics showing the percentage of uniquely mapped, multi-mapped and unmapped reads. **e.** Distribution of mapped reads between introns, intergenic regions and 3'-UTR / 5'-UTR / coding sequence (=CDS) exons. Expressed in percentage of read tags and computed using ReSQC. **f.** General trend in GC-content distribution expressed in percentage of mapped reads and measured using ReSQC. Individual variations were summarised by fitting a generalized additive model implemented in *geom_smooth* (ggplot2) using the formula [y ~ s(x, bs = "cs")]. The vertical red line represents the average GC-content (%) in genes expressed ≥3 cells sequenced in this experiment (downsampled to 250K reads). **g.** GC-content (%) of genes with improved (logFC≥1, $n_{1.65mM}$=35, $n_{3.3mM}$=143, $n_{5.5mM}$=194, $n_{8.25mM}$=316, n_{11mM}=439) or unchanged detection in the presence of various dCTP amounts. Differential expression was performed using a t-test on log-normalized and library size normalized gene counts from cells downsampled to 250K raw reads (*scater*). Genes expressed in <5 cells were discarded ($n_{Total\_expressed\_genes}$=17,916).The difference between DE and non-DE genes was evaluated using a Wilcoxon-rank sum test (two-sided, Bonferroni adj. *P*-value). **h.** Mismatch per 100K mapped base measured with STAR (n_{0mM}=19, $n_{1.65mM}$=20, $n_{3.3mM}$=26, $n_{5.5mM}$=27, $n_{8.25mM}$=18, n_{11mM}=28) **i.** Number of detected substitutions in each condition per 100K uniquely mapped reads (n_{0mM}=32, $n_{1.65mM}$=31, $n_{3.3mM}$=30, $n_{5.5mM}$=32, $n_{8.25mM}$=31, n_{11mM}=30). Comparisons were performed with the 'no dCTP' condition as reference using a two-sided Wilcoxon rank sum test (Bonferroni adj. *P*-value), subdivided by substitution quality. **j.** Percentage of each detected substitution ($n_{total\_substitutions}$=513235). Top panel shows higher quality variants (QUAL ≥ 20 and read depth > 4).

Multiple comparisons in (a), (c), (h) were performed using a Kruskal-Wallis test followed by a two-sided Dunn's test. All *P*-values were adjusted for multiple-testing with Bonferroni correction.

GTP (1 mM) [HEK-5µl, 250K raw reads], Fig E15: SS3 reported that using GTP can be beneficial to the reaction, helping stabilize and/or promote template-switching. Therefore, we replaced the dCTP in the FS protocol (3.3 mM final) with 1 mM GTP. Our results indicate that GTP does not seem to bring any benefit to the FS reaction.

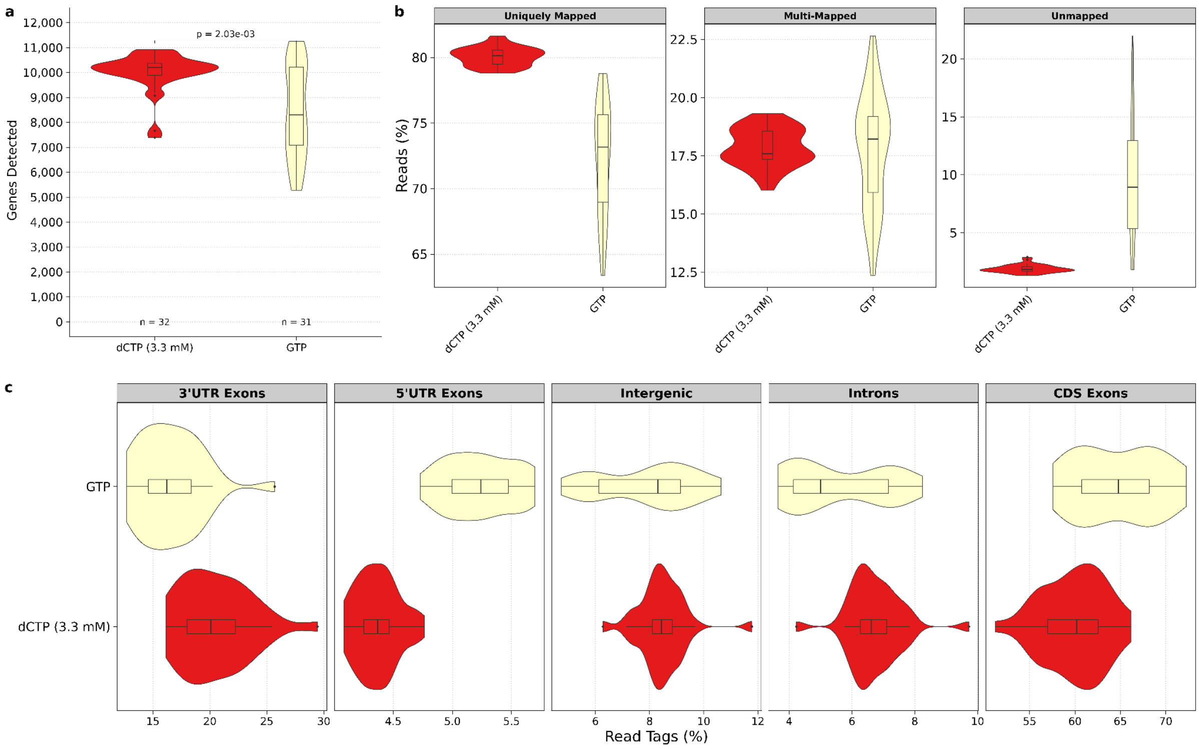

Fig E15 | HEK - 5µl - GTP (1 mM) a. Number of genes detected in cells processed with a RT-PCR buffer containing 3.3 mM dCTP (*n*=32) or 1 mM GTP (*n*=31) (Wilcoxon rank sum test, two-sided, *P*-value). **b.** STAR mapping statistics showing the percentage of uniquely mapped, multi-mapped and unmapped reads. **c.** Distribution of mapped reads between introns, intergenic regions or 3'-UTR / 5'-UTR / coding sequence (=CDS) exons. Expressed in percentage of read tags and computed using ReSQC.

cDNA amplification

Pfu DNA polymerase (0.25-0.375 U / reaction) [hPBMCs-5µl], Fig E16: the addition of *Pyrococcus furiosus* DNA polymerase (a high-fidelity DNA polymerase, NEB) to the RT-PCR mix did not significantly increase the number of genes detected.

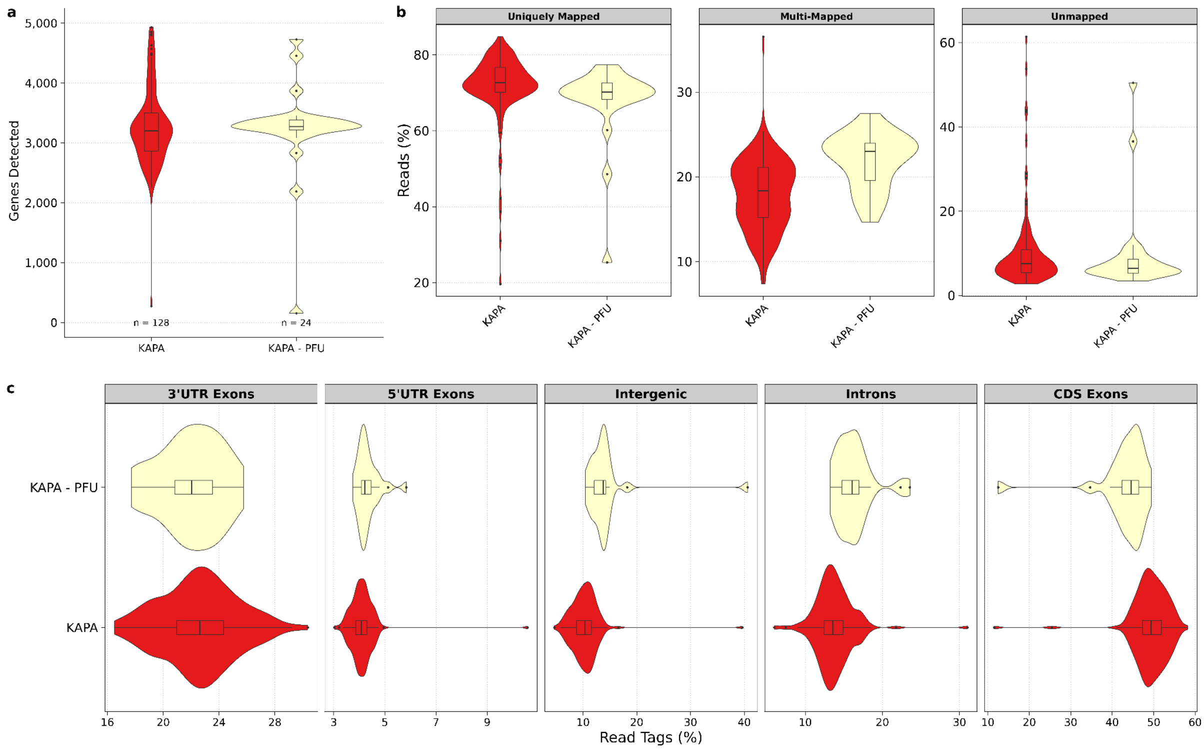

Fig E16 | hPBMCs - 5µl - Pfu DNA polymerase a. Number of detected genes. No significant difference was observed between KAPA polymerase (*n*=128) alone and KAPA+Pfu polymerase (*n*=24) (Wilcoxon rank sum test, two-sided, *P*-value). **b.** STAR mapping statistics showing the percentage of uniquely mapped, multi-mapped and unmapped reads. **c.** Distribution of mapped reads between introns, intergenic regions or 3'-UTR / 5'-UTR / coding sequence (=CDS) exons. Expressed in percentage of read tags and computed using ReSQC.

PCR extension time (4 *vs* 6 min) [hPBMCs-5µl], Fig E17: while other methods (i.e., SS3[4], SSsc [Takara Bio]) perform a 4-min extension step in the pre-amplification reaction, we detected more genes when using 6 minutes.

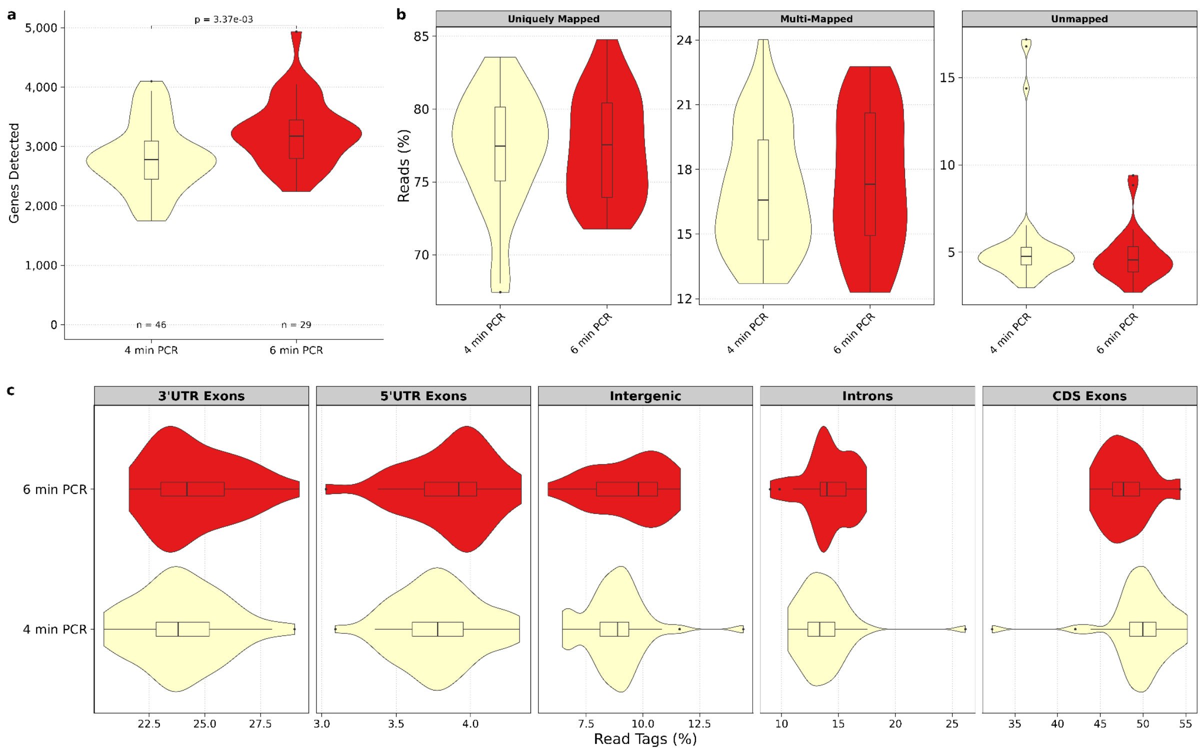

Fig E17 | hPBMCs - 5µl - PCR extension time a. Number of genes detected in cells processed with a PCR cycle duration of 4 (*n*=46) or 6 minutes (*n*=29) (Wilcoxon rank sum test, two-sided, *P*-value). **b.** STAR mapping statistics showing the percentage of uniquely mapped, multi-mapped and unmapped reads. **c.** Distribution of mapped reads between introns, intergenic regions or 3'-UTR / 5'-UTR / coding sequence (=CDS) exons. Expressed in percentage of read tags and computed using ReSQC.

Extreme thermostable single-stranded DNA binding protein (ET SSB, NEB: 0, 40 ng, 80 ng, 160 ng, 250 ng) [hPBMCs-5µl], Fig E18: ET SSB is reported to be a PCR additive enhancing DNA polymerase activity and increasing PCR yield. It remains active even at high temperatures and for prolonged periods (~60 min, 95°C). In our settings, the addition of ET SSB did not improve cDNA yield nor the number of genes recovered. Using 250 ng ET SSB/samples appeared to inhibit the reaction altogether.

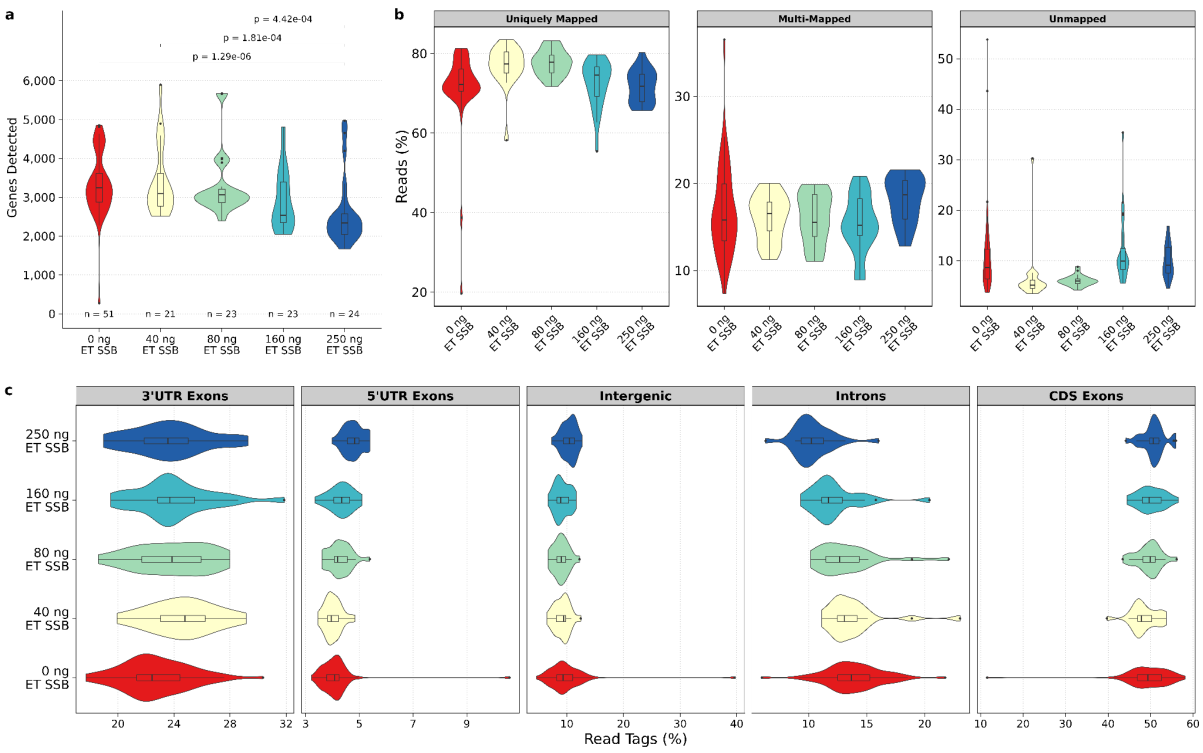

Fig E18 | hPBMCs - 5 µl - ET SSB titration a. Number of genes detected in cells processed in a RT-PCR buffer containing of 0 ng (*n*=51), 40 ng (*n*=21), 80 ng (*n*=23), 100 ng (*n*=23), 250 ng (*n*=24) ET SSB (Dunn's test, two-sided, Bonferroni correction, adj. *P*-value). **b.** STAR mapping statistics showing the percentage of uniquely mapped, multi-mapped and unmapped reads. **c.** Distribution of mapped reads between introns, intergenic regions or 3'-UTR / 5'-UTR / coding sequence (=CDS) exons. Expressed in percentage of read tags and computed using ReSQC.

Extra dNTPs (1.2 mM *vs* no additional dNTP) [HEK-5µl, 50K] Fig E19: the dNTPs used in FLASH-seq come from 2 different sources: the lysis buffer mix (final concentration of 1.2 mM in RT-PCR) and the 2 x KAPA HiFi HotStart ReadyMix (0.3 mM each in the final 1 x mix). The addition of dNTPs to the lysis buffer had previously been shown to stabilize mRNA molecules during denaturation, although the minimum amount required for an efficient RT reaction was never tested[10].

Therefore, we sequenced HEK 293T cells processed with either 1.5 mM dNTPs (1.2 mM + 0.3 mM, "full dNTPs") or just the 0.3 mM cotained in the KAPA ReadyMix ("no dNTPs"). We observed a significant reduction in the number of genes detected when omitting dNTPs in the lysis buffer.

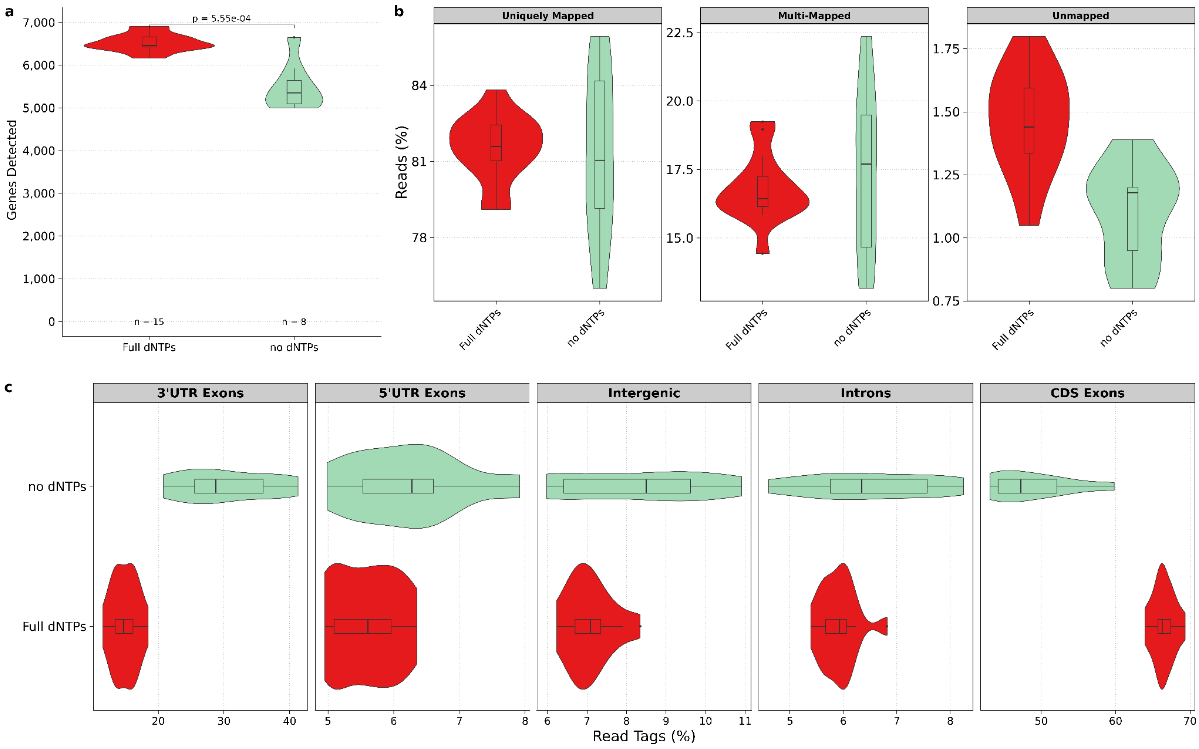

Fig E19 | HEK - 5µl - Extra dNTPs a. Number of genes detected in cells processed with dNTPs (*n*=15) or no dNTP (*n*=8) in the lysis buffer (Wilcoxon rank sum test, two-sided, *P*-value). **b.** STAR mapping statistics showing the percentage of uniquely mapped, multi-mapped and unmapped reads. **c.** Distribution of mapped reads between introns, intergenic regions or 3'-UTR / 5'-UTR / coding sequence (=CDS) exons. Expressed in percentage of read tags and computed using ReSQC.

T4 gene 32 protein (T4g32p)

T4g32p is a single-strand binding (SSB) protein derived from the T4 bacteriophage, which stabilizes single-strand DNA and RNA and has been shown to enhance the SMART-seq reaction efficiency in the first iterations of the method[12]. More recently, it has been used in the single-cell protocol “RAMDA-seq” to promote strand-displacement and protect newly synthesized cDNA from exonuclease treatment[13]. T4g32p has an optimal temperature of 37°C. The addition of 5 µg T4g32p (NEB) to the RT-PCR mix had a dramatic impact on the 25 µl reaction, increasing the cDNA yield by 2.8-fold in HEK 293T cells and by 4.3-fold in hPBMCs (Fig E20a). However, this was accompanied by a significant increase in shorter cDNA fragments, which could be eliminated in the following bead cleanup only by decreasing the cDNA / magnetic bead ratio from the standard 1:0.8 to 1:0.6 (Fig E20b).

The addition of 5 µg T4g32p to the reaction had a positive impact on the number of genes detected in hPBMCs (Fig E20c). Unfortunately, it also increased the percentage of multi-mapped and unmapped reads (Fig E20d). The origin of multi-mapped reads can likely be traced back to the lower RT temperature (see Fig E9) and decreased stringency. On the other hand, we were unfortunately unable to assess the source of the unmapped reads.

We did not observe an increase in cDNA yield when performing the RT at 50°C in the presence of T4g32p, indicating that T4g32p is likely inactivated at higher temperatures. Interestingly, T4g32p increased the cDNA yield only marginally in a 5-µl reaction, without improving gene detection (see T4g32p titration in hPBMCs, Fig E20). We made several attempts to modify the reaction conditions with no significant increase in the number of genes detected. We conclude that the benefits of miniaturizing the reaction may compensate for the addition of T4g32p. Perhaps equally important, T4g32p increases the cost per 96 cells by ~$230 (25-µl reaction), which could instead be used for deeper sequencing. We therefore do not recommend its use.

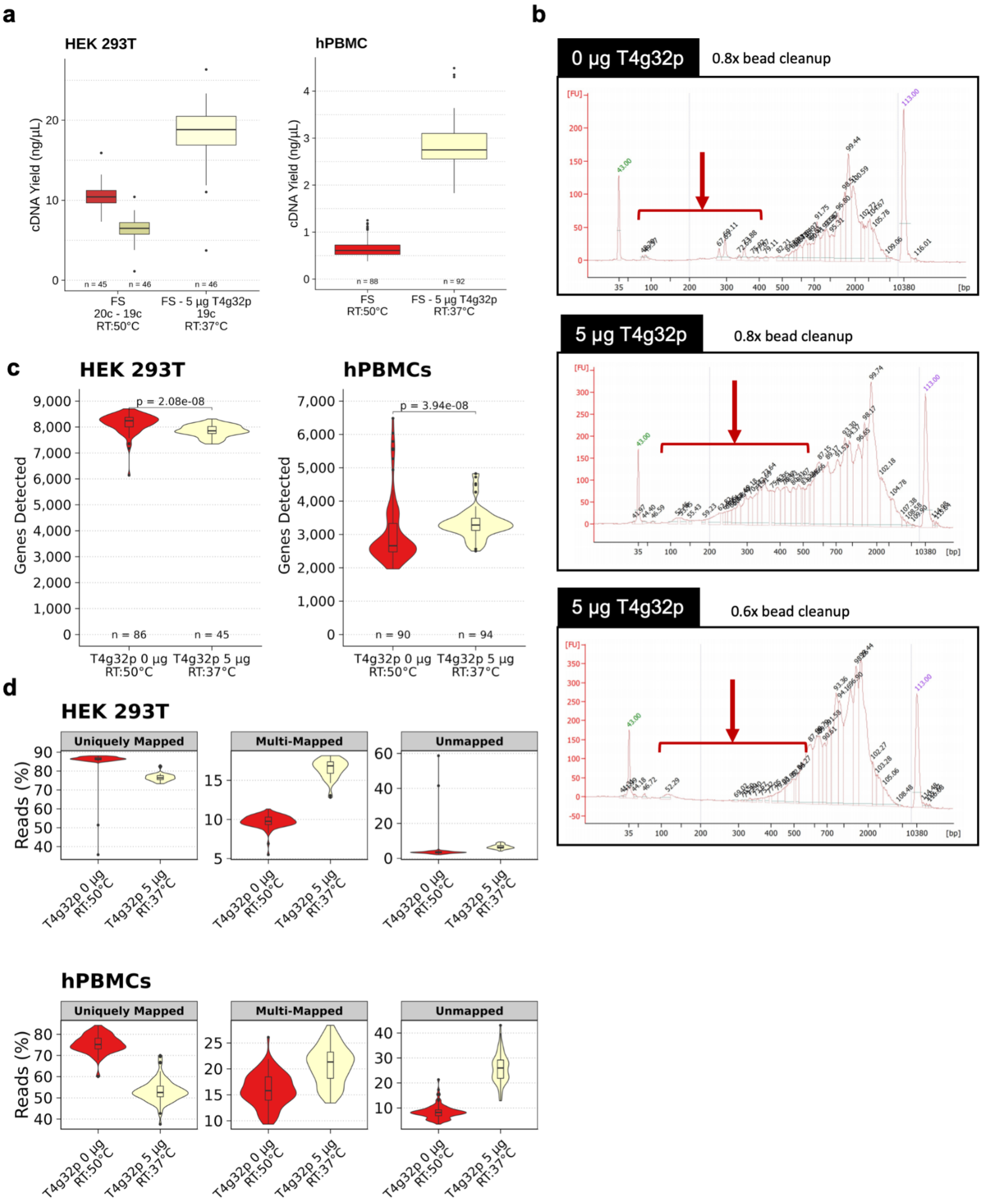

Fig E20 | hPBMCs & HEK 293T - 25 µl - Effect of T4g32p on FS reaction. a.cDNA yield in HEK 293T cells (left) or hPBMCs (right) in the presence or absence of 5 µg T4g32p (25 µl reaction volume). **b.** Bioanalyzer traces of selected cells processed in the absence of T4g32p (top) or with 5 µg T4g32p and cleaned using a ratio of magnetic beads / cDNA of either 1:0.8 (mid) or 1:0.6 (bottom). Red arrows highlight the excess of short fragments (<500 bp). **c.** Number of genes detected in either HEK 293T cells (left, $n_{0µg\_T4g32}$=86, $n_{5µg\_T4g32}$=45) or hPBMCs (250K raw reads, right, $n_{0µg\_T4g32}$=90, $n_{5µg\_T4g32}$=94) in the presence or absence of T4g32p (Wilcoxon rank sum test, two-sided, *P*-value) (25 µl final volume). **d.** STAR mapping statistics showing the percentage of uniquely mapped, multi-mapped and unmapped reads in either HEK 293T cells (left) or hPBMCs (right) in the presence or absence of T4g32p.

T4g32p titration [hPBMCs-5µl], Fig E21: The amount of T4g32p was titrated in a 5-µl reaction by adding 0.125, 0.25, 0.50, 0.75 or 1 µg of T4g32p to the reaction and comparing the results with standard FS (no T4g32p, RT-50°C). In contrast to the 25-µl reaction volume, the addition of T4g32p in a smaller volume did not significantly increase the number of genes detected. These results seem to suggest that in a final volume of 5 µl, the addition of T4g32p is not required.

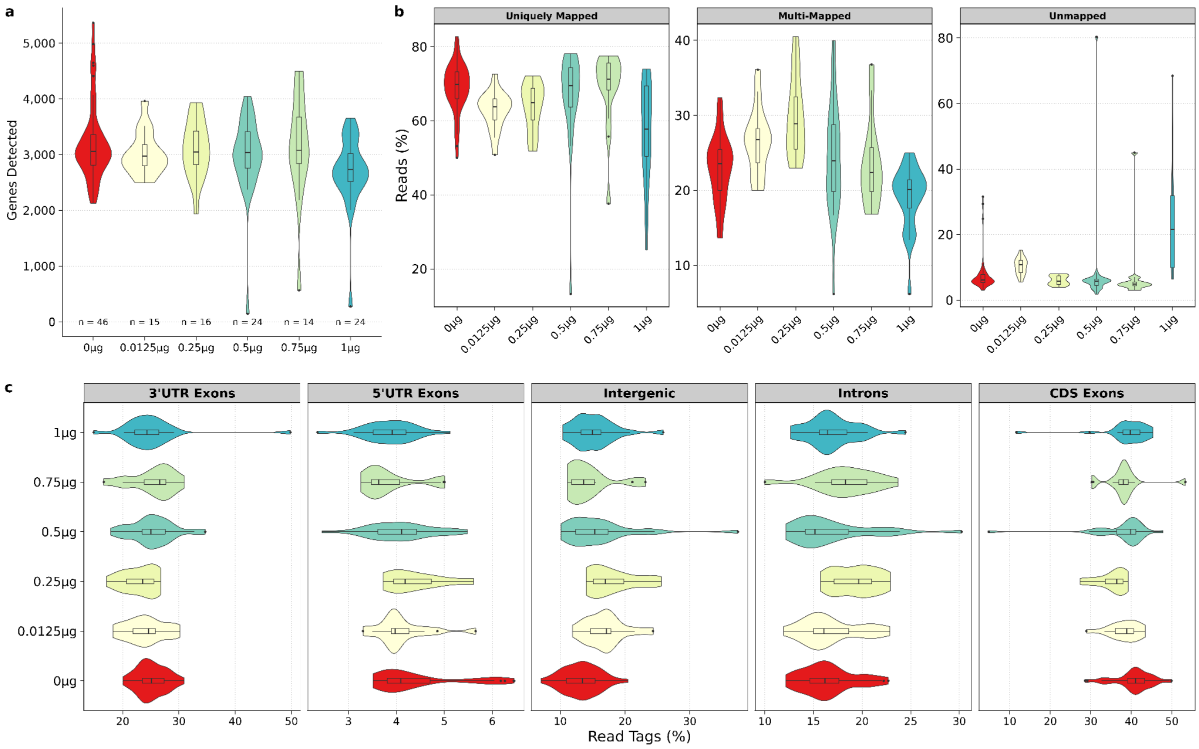

Fig E21 | hPBMCs - 5µl - T4g32p titration a. Number of genes detected in cells processed with 0 µg (*n*=46), 0.0125 µg (*n*=15), 0.25 µg (*n*=16), 0.5 µg (*n*=24), 0.75 µg (*n*=14), 1 µg (*n*=24) T4g32p (Dunn's test, two-sided, Bonferroni correction, adj. *P*-value). **b.** STAR mapping statistics showing the percentage of uniquely mapped, multi-mapped and unmapped reads. **c.** Distribution of mapped reads between introns, intergenic regions or 3'-UTR / 5'-UTR / coding sequence (=CDS) exons. Expressed in percentage of read tags and computed using ReSQC.

T4g32p 0.25 µg + FS SMART-dT$_{30}$VN (5- or 10-times less than the standard FS [1.8µM]) [hPBMCs-5µl], Fig E22: similarly to the reaction without T4g32p, we did not observe a difference in the number of genes detected when using 5- or 10-times less FS SMART-dT$_{30}$VN oligo. We decided to continue working with 5-times less FS SMART-dT$_{30}$VN oligo, as the leftover primer dimers can be efficiently removed by magnetic bead cleanup.

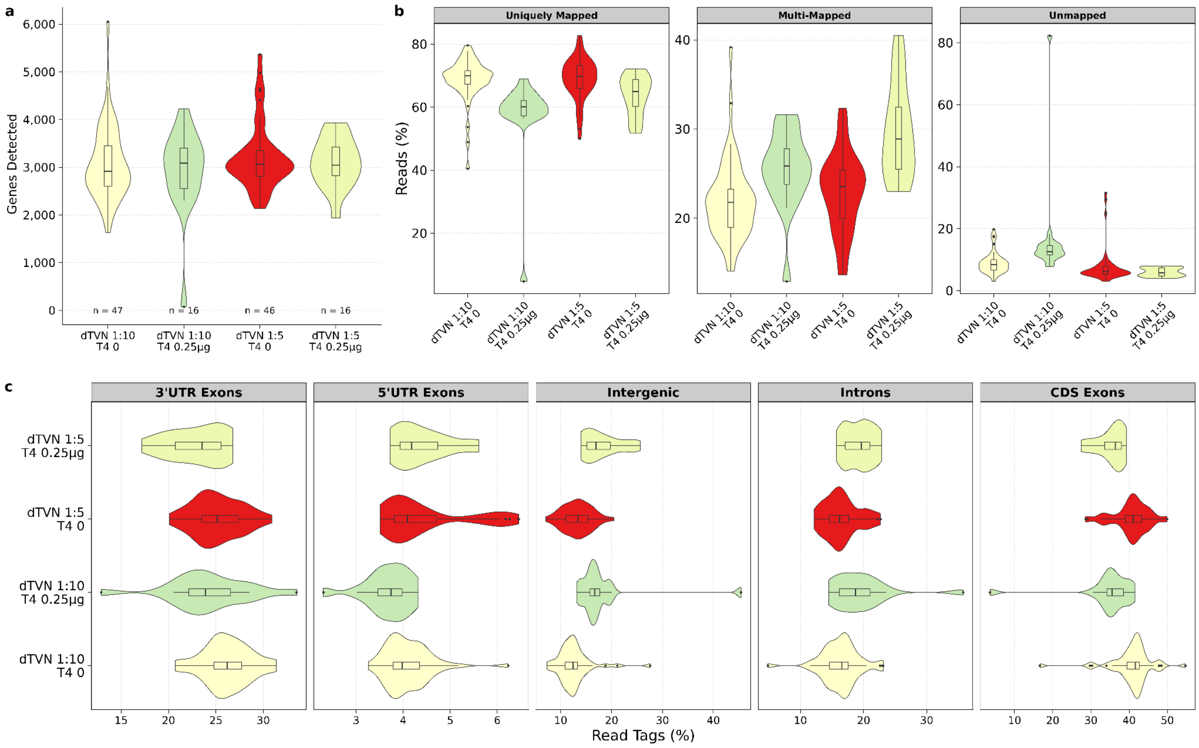

Fig E22 | hPBMCs - 5µl - T4g32p and FS SMART-dT$_{30}$VN a. Number of genes detected in cells processed in the presence of 0 µg T4g32p / 1:10 FS SMART-dT$_{30}$VN (*n*=47), 0.25 µg T4g32p / 1:10 FS SMART-dT$_{30}$VN (*n*=16), 0 µg T4g32p / 1:5 FS SMART-dT$_{30}$VN (*n*=46), 0.25 µg T4g32p / 1:5 FS SMART-dT$_{30}$VN (*n*=16) (Dunn's test, two-sided, Bonferroni correction, adj. *P*-value). **b.** STAR mapping statistics showing the percentage of uniquely mapped, multi-mapped and unmapped reads. **c.** Distribution of mapped reads between introns, intergenic regions or 3'-UTR / 5'-UTR / coding sequence (=CDS) exons. Expressed in percentage of read tags and computed using ReSQC.

T4g32p 0.25/0.50/0.75 µg + Maxima H- or Superscript IV [hPBMCs-5µl], Fig E23: Similarly to the reaction without T4g32p, we did not observe a significant difference between the 2 enzymes.

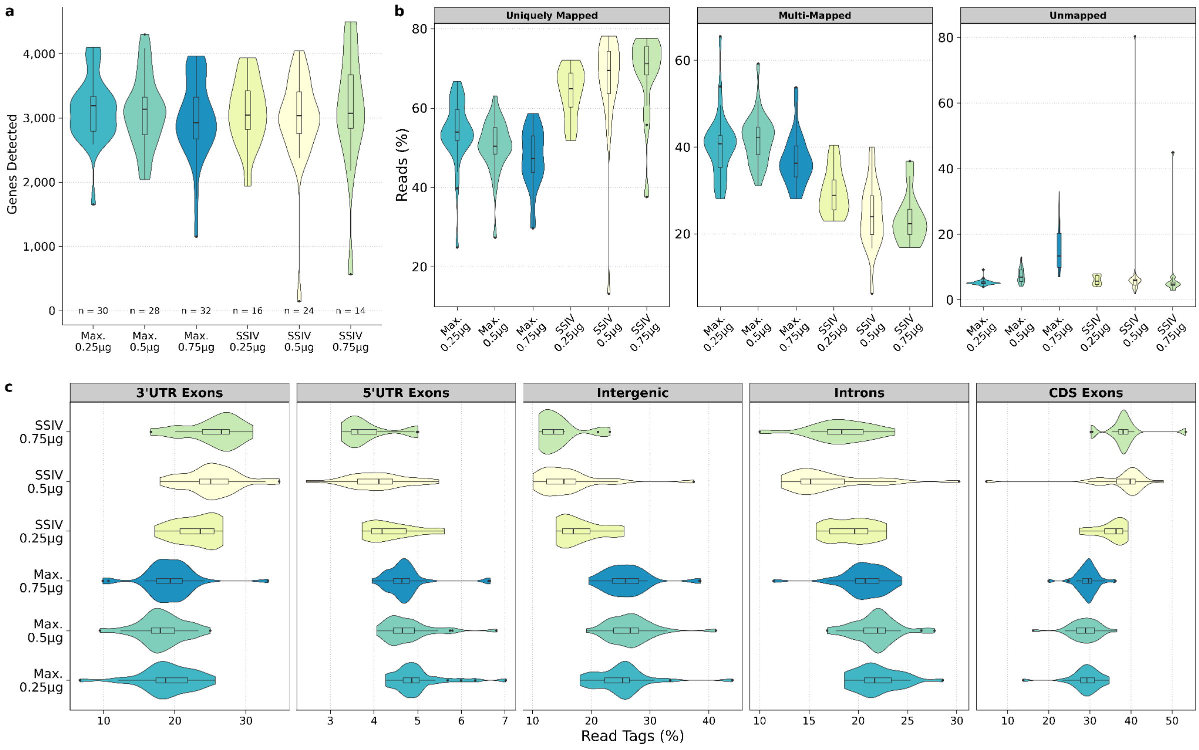

Fig E23 | hPBMCs - 5µl - T4g32p titration and reverse transcriptase a. Number of genes detected in cells reverse transcribed with 0.25 µg T4g32p + Maxima H- (*n*=30), 0.5 µg T4g32p + Maxima H- (*n*=28), 0.75 µg T4g32p + Maxima H- (*n*=32), 0.25 µg T4g32p + Superscript IV(*n*=16), 0.5 µg T4g32p + Superscript IV (*n*=24) or 0.75 µg T4g32p + Superscript IV (*n*=14) (Dunn's test, two-sided, Bonferroni correction, adj. *P*-value). **b.** STAR mapping statistics showing the percentage of uniquely mapped, multi-mapped and unmapped reads. **c.** Distribution of mapped reads between introns, intergenic regions or 3'-UTR / 5'-UTR / coding sequence (=CDS) exons. Expressed in percentage of read tags and computed using ReSQC.

T4g32p 0.25 / 0.5 / 0.75 µg + variable RT duration (30 or 60 min) [hPBMCs-5µl], Fig E24: we observed a trend towards a lower number of genes detected when performing the RT for 30 min compared to 60 min in the presence of T4g32p. Of note, the libraries for the "30 min RT" samples were prepared with the plexWell standard input kit (seqWell) while the libraries for the "60 min RT samples" were prepared with the Nextera XT kit (Illumina).

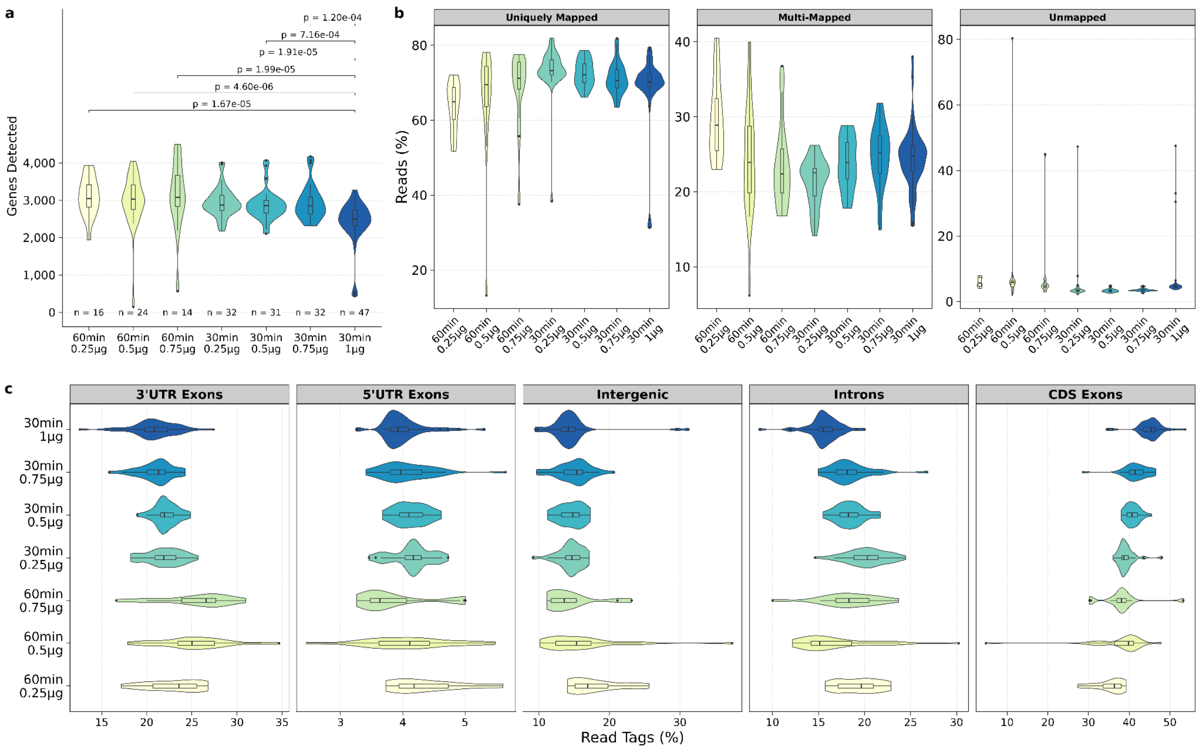

Fig E24 | hPBMCs - 5µl - T4g32p titration and RT duration a. Number of genes detected in seven reaction conditions with varying RT duration, T4g32p amounts and library preparation methods ($n_{60min\_0.25µgT4\_Next.}$=16, $n_{60min\_0.5µgT4\_Next.}$=24, $n_{60min\_0.75µgT4\_Next.}$=14, $n_{30min\_0.25µgT4\_Plex.}$=32, $n_{30min\_0.5µgT4\_Plex.T}$=31, $n_{30min\_0.75µgT4\_Plex.}$=32, $n_{30min\_1µgT4\_Plex.}$=47) (Dunn's test, two-sided, Bonferroni correction, adj. *P*-value). **b.** STAR mapping statistics showing the percentage of uniquely mapped, multi-mapped and unmapped reads. **c.** Distribution of mapped reads between introns, intergenic regions or 3'-UTR / 5'-UTR / coding sequence (=CDS) exons. Expressed in percentage of read tags and computed using ReSQC.

T4g32p 0.25 / 0.5 /1 µg + Triton X-100 1.2% / 0.2% [hPBMCs-5µl], Fig E25: in general, we detected a higher number of genes when using 0.2% Triton X-100, regardless of the amount of T4g32p.

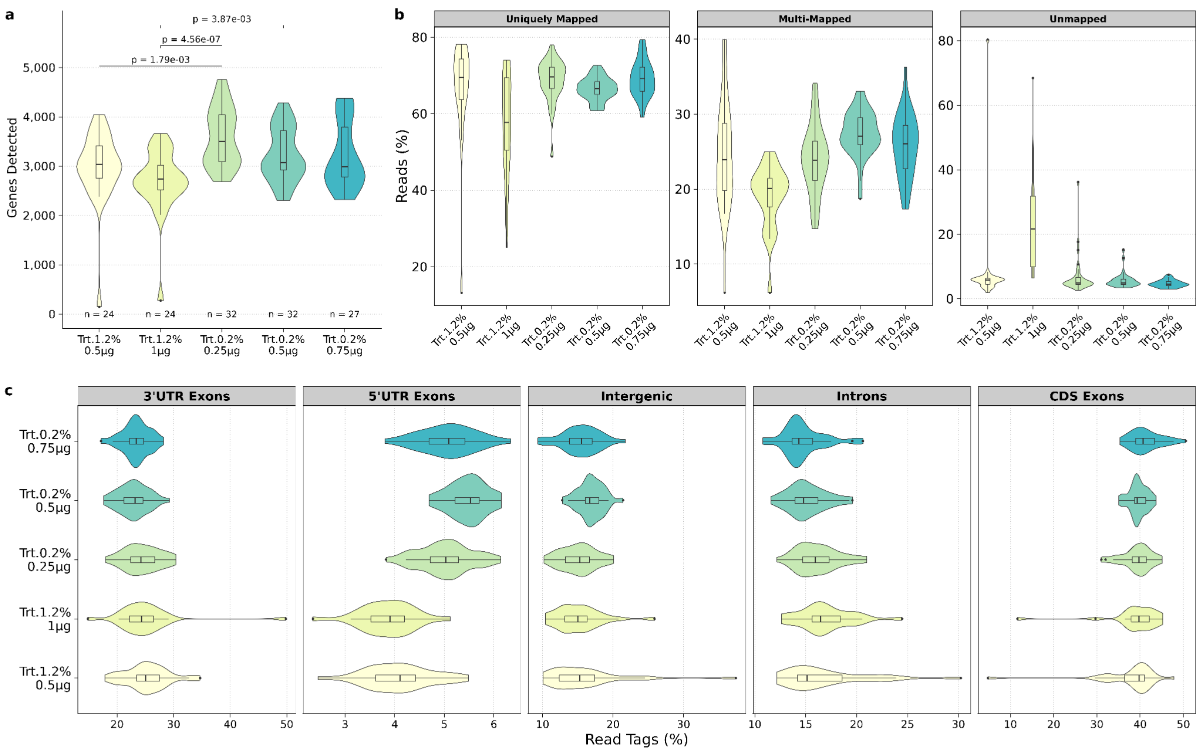

Fig E25 | hPBMCs - 5µl - T4g32p titration and lysis buffer a. Number of genes detected in seven reaction conditions with varying amount of Triton X-100 and T4g32p ($n_{triton1.2\%\_0.5\mu gT4}$=24, $n_{triton1.2\%\_1\mu gT4}$=24, $n_{triton0.2\%\_0.25\mu gT4}$=32, $n_{triton0.2\%\_0.5\mu gT4}$=32, $n_{triton0.2\%\_1\mu gT4}$=27) (Dunn's test, two-sided, Bonferroni correction, adj. *P*-value). **b.** STAR mapping statistics showing the percentage of uniquely mapped, multi-mapped and unmapped reads. **c.** Distribution of mapped reads between introns, intergenic regions or 3'-UTR / 5'-UTR / coding sequence (=CDS) exons. Expressed in percentage of read tags and computed using ReSQC.

T4g32p 0.25 µg + TSO (1 x, 2 x, 3 x, 4 x) [hPBMCs-5µl], Fig E26: as in the no-T4g32p condition, we observed a linear relationship between amount of TSO and number of genes detected. This effect appeared to be less pronounced than in the no-T4g32p condition (i.e., adj. P-value > 0.05) but was still associated with an increased number of multi-mapped and intergenic reads and seem to indicate that increasing the amount of TSO does not bring any benefits.

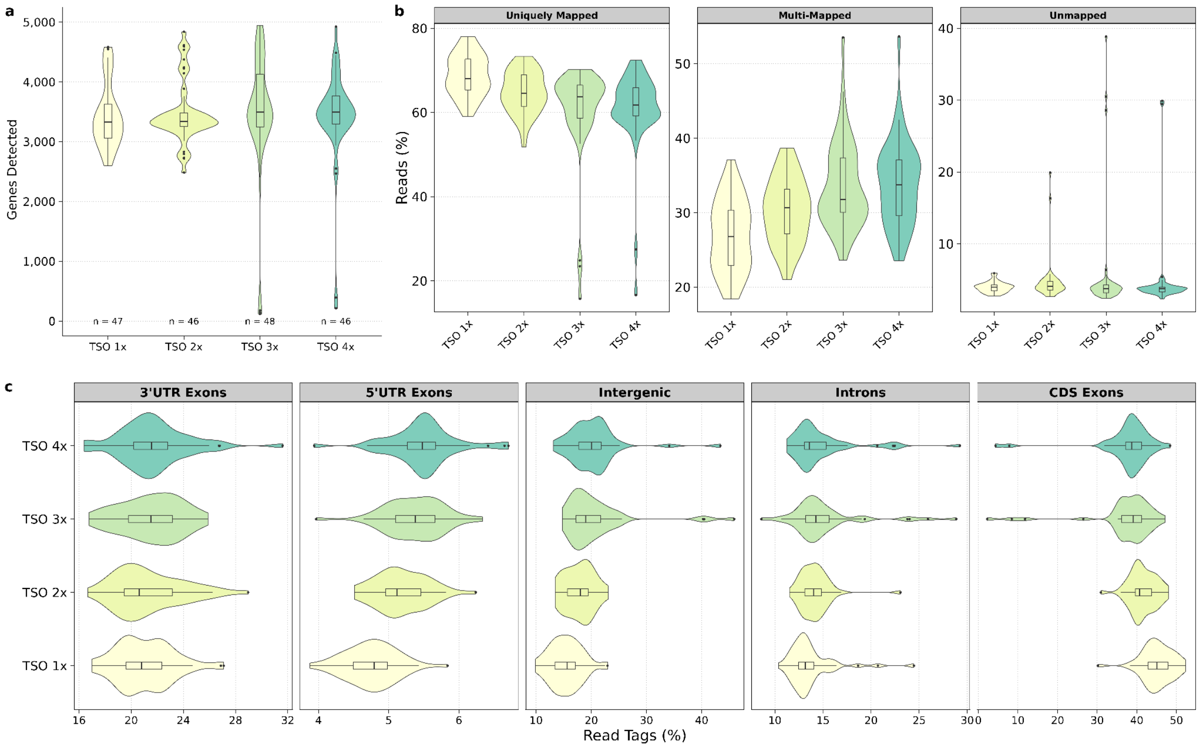

Fig E26 | hPBMCs - 5µl - T4g32p and TSO titration **a.** Number of genes detected in the presence of T4g32p and 1- (*n*=47), 2- (*n*=46), 3- (*n*=48) or 4-times (*n*=46) the standard amount of TSO (Dunn's test, two-sided, Bonferroni correction, adj. *P*-value). **b.** STAR mapping statistics showing the percentage of uniquely mapped, multi-mapped and unmapped reads. **c.** Distribution of mapped reads between introns, intergenic regions or 3'-UTR / 5'-UTR / coding sequence (=CDS) exons. Expressed in percentage of read tags and computed using ReSQC.

Home-made T4g32p [hPBMCs-25µl], Fig E27: in our tests we always observed a higher percentage of unmapped reads when using T4g32p. To rule out contaminations by residual DNA/RNA related to the NEB T4g32p production (e.i. bacterial), we used a home-made T4g32p which had been thoroughly cleaned by dialysis. Similarly to the commercial enzyme, we observed a higher percentage of multi-mapped, unmapped and intergenic reads. We conclude that these undesired side-effects are intrinsic to the T4g32p activity and are not related to the enzyme manufacturing process.

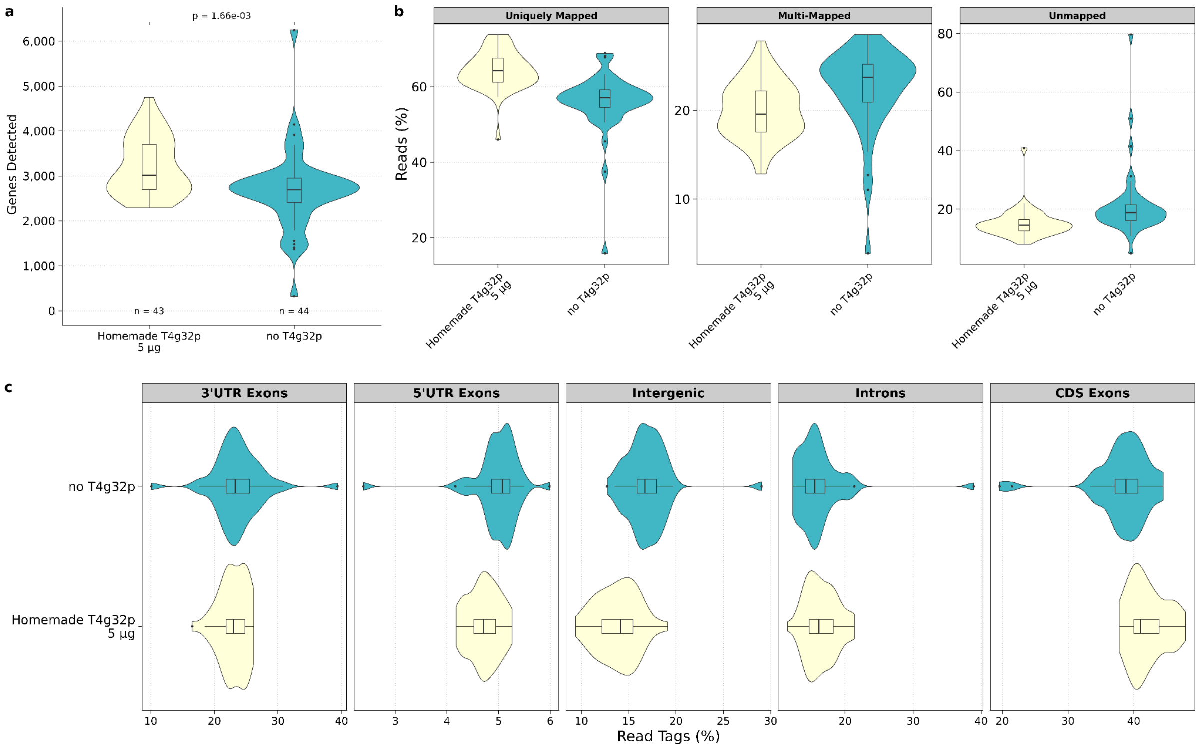

Fig E27 | hPBMCs - 25µl - home-made T4g32p a. cDNA size distribution **b.** Number of genes detected in cells processed with home-made (*n*=43) or commercial (*n*=44) T4g32p (Wilcoxon rank sum test, two-sided, *P*-value). **c.** STAR mapping statistics showing the percentage of uniquely mapped, multi-mapped and unmapped reads. **d.** Distribution of mapped reads between introns, intergenic regions or 3'-UTR / 5'-UTR / coding sequence (=CDS) exons. Expressed in percentage of read tags and computed using ReSQC.

Conclusions

The development of FLASH-seq is the result of years of observations accumulated after publishing Smart-seq2[10]. Building on this knowledge, we explored many conditions before settling on the protocol described in this study. As perhaps to be expected, the vast majority of these changes did not bring any significant benefit in terms of number of genes detected or overall data quality. Our results indicate that the improvement of single-cell protocols through additives combinations or optimized reaction conditions may have reached its limit. A new generation of enzymes with higher processivity and sensitivity when dealing with sub-picogram levels of RNA is therefore urgently needed to investigate very lowly expressed genes.

In several instances, carrying out the reactions in smaller volumes often annulled the effect that several additives showed in larger volumes. In addition, we also observed significant differences between large (i.e., HEK 293T) and small cells (i.e., hPBMCs), highlighting the need to perform the tests directly in the conditions which better reflect the “real” experiment.

In conclusion, several additives/reaction conditions such as variable TSO concentrations or different lysis buffers (i.e., BSA or GuHCl) may give an advantage in specific reaction settings, but will likely require more in-depth testing.

Nonetheless, we hope that the results presented here can provide valuable information and help build the next generation of single-cell sequencing methods.

References Supplementary Discussion

1. Kreader, C. A. Relief of amplification inhibition in PCR with bovine serum albumin or T4 gene 32 protein. *Appl. Environ. Microbiol.* **62**, 1102–1106 (1996).
2. Ellis, R. J. Macromolecular crowding: obvious but underappreciated. *TRENDS Biochem. Sci.* **26**, 8 (2001).
3. Svec, D. *et al.* Direct Cell Lysis for Single-Cell Gene Expression Profiling. *Front. Oncol.* **3**, (2013).
4. Hagemann-Jensen, M. *et al.* Single-cell RNA counting at allele and isoform resolution using Smart-seq3. *Nat. Biotechnol.* **38**, 708–714 (2020).
5. Hochgerner, H. *et al.* STRT-seq-2i: dual-index 5' single cell and nucleus RNA-seq on an addressable microwell array. *Sci. Rep.* **7**, (2017).
6. Picelli, S. *et al.* Tn5 transposase and tagmentation procedures for massively scaled sequencing projects. *Genome Res.* **24**, 2033–2040 (2014).
7. Adey, A. & Shendure, J. Ultra-low-input, tagmentation-based whole-genome bisulfite sequencing. *Genome Res.* **22**, 1139–1143 (2012).
8. Zhu, Y. Y., Machleder, E. M., Chenchik, A., Li, R. & Siebert, P. D. Reverse Transcriptase Template Switching: A SMART™ Approach for Full-Length cDNA Library Construction. **30**, 6 (2001).
9. Zajac, P., Islam, S., Hochgerner, H., Lönnerberg, P. & Linnarsson, S. Base Preferences in Non-Templated Nucleotide Incorporation by MMLV-Derived Reverse Transcriptases. *PLoS ONE* **8**, e85270 (2013).
10. Picelli, S. *et al.* Smart-seq2 for sensitive full-length transcriptome profiling in single cells. *Nat. Methods* **10**, 1096–1098 (2013).
11. Wulf, M. G. *et al.* Non-templated addition and template switching by Moloney murine leukemia virus (MMLV)-based reverse transcriptases co-occur and compete with each other. *J. Biol. Chem.* **294**, 18220–18231 (2019).
12. Villalva, C. *et al.* Increased Yield of PCR Products by Addition of T4 Gene 32 Protein to

the SMART^{TM} PCR cDNA Synthesis System. *BioTechniques* **31**, 81–86 (2001).

13. Hayashi, T. *et al.* Single-cell full-length total RNA sequencing uncovers dynamics of recursive splicing and enhancer RNAs. *Nat. Commun.* **9**, 619 (2018).

Supplementary Notes

Supplementary Note 1

Guidelines - FLASH-seq Low-Amplification

When processing cells using FLASH-seq Low-Amplification (FS-LA) protocol, 3 important parameters must be taken into account:

- The cell RNA content, which determines how many pre-amplification cycles are required to generate enough cDNA for the tagmentation, while minimizing unmapped / intergenic reads. We hypothesise that the excess of intergenic/unmapped reads may originate from a mixture of tagmentation of the genomic DNA (gDNA) and overtagmentation of the messenger RNA (mRNA).
- The reaction volume. As the cDNA is not purified prior to tagmentation, it is important to find the right balance between diluting enough, but not too much, the salts and additives of the RT-PCR mix, in order to avoid an unnecessary waste of reagents, which ultimately results in a higher cost per cell. We recommend diluting the unpurified cDNA 10 times for the best results, although lower dilutions might also work.
- The amount of Tn5 needs to be adjusted case by case.

In the following paragraphs we provide some recommendations regarding the adjustment of these parameters.

cDNA pre-amplification

To determine the adequate number of PCR cycles we relied on estimating the ratio between the cell mRNA and gDNA. We first estimated the mRNA content based on the amount of total RNA recovered from $1x10^6$ cells. For instance, extracting total RNA from HEK 293T cells generates ~16 µg[1] of RNA while hPBMCs give ~8 µg[1]. Among hPBMCs, dendritic cells are closer to ~4 µg[1]. We then assumed the worst and unlikely scenario, in which the entire cell gDNA would be available for tagmentation (= 3.1 pg gDNA). Assuming 5% of the cell RNA is mRNA and that we have a ~60% PCR efficiency (see Fig 2b), we can draw the following table to estimate the amount of cDNA generated after each PCR cycle:

	HEK 293T Cells (~16 pg total RNA)		hPBMCs (~ 4 pg total RNA)	
PCR Cycles	mRNA	Ratio mRNA / gDNA	mRNA	Ratio mRNA / gDNA
Starting Material	0.8	0.3	0.2	0.1
1	1.3	0.4	0.3	0.1
2	2.0	0.7	0.5	0.2
3	3.3	1.1	0.8	0.3
4	5.2	1.7	1.3	0.4
5	8.4	2.7	2.1	0.7
6	13.4	4.3	3.4	1.1
7	21.5	6.9	5.4	1.7
8	34.4	11.1	8.6	2.8
9	55.0	17.7	13.7	4.4
10	88.0	28.4	22.0	7.1
11	140.7	45.4	35.2	11.3
12	225.2	**72.6**	56.3	18.2
13	360.3	116.2	90.1	29.1
14	576.5	186.0	144.1	46.5
15	922.3	297.5	230.6	74.4
16	1475.7	476.0	368.9	**119.0**
17	2361.2	761.7	590.3	190.4
18	3777.9	1218.7	944.5	304.7
19	6044.6	1949.9	1511.2	487.5

As shown in the paper, we titrated the number of PCR cycles required to minimize the percentage of intergenic/unmapped reads in HEK 293T cells. These values stabilized around 10-12 PCR cycles. This would be equivalent to a mRNA / gDNA ratio of ~73-fold.

We then tested hPBMCs, assuming all cells had an mRNA content comparable to the smallest ones in the population (i.e., dendritic cells, 4 µg from $1x10^6$ cells). Applying the same line of reasoning, we settled on 16 PCR cycles, to reach a ratio similar to HEK 293T cells. We obtained high-quality libraries on the first test.

When processing a new cell type, an initial titration experiment is always recommended. This experiment aims to compare the number of intergenic/unmapped/uniquely mapped reads obtained when processing the cells with standard FS (i.e., 19-23 cycles) and FS-LA (10-16 cycles).

We foresee that the adequate number of PCR cycles for most cell types will be around 12-14 PCR cycles for most cell types.

Tagmentation

Similarly to other Smart-seq protocols, the amount of Tn5 used may have to be adjusted, to generate sequencing libraries with an insert size between 300 and 600 bp. When working with minute amounts of cDNA, overtagmentation can become an issue, as illustrated below:

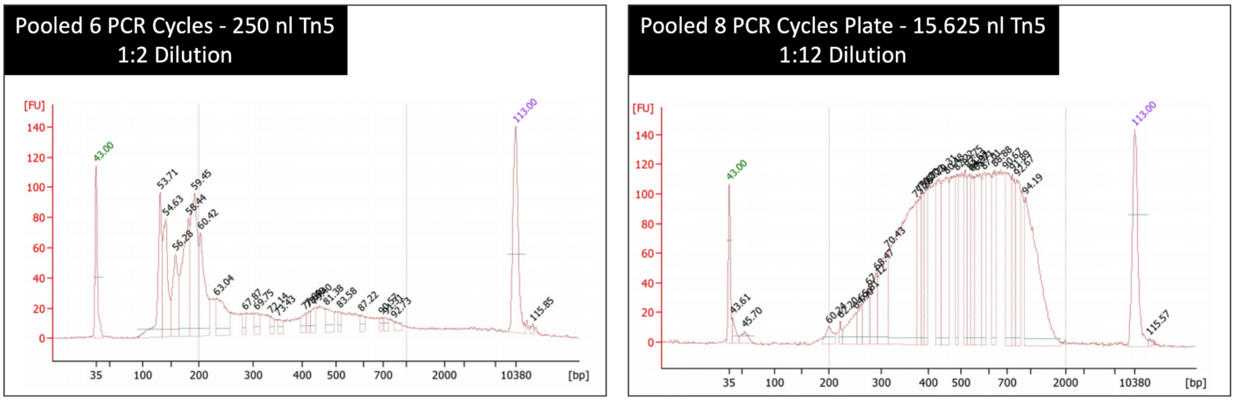

While we typically use 250 nl of our home-made Tn5 to prepare sequencing libraries with the standard FS (150 pg), values between 8- (31.125 nl) and 16- (15.625 nl) times less Tn5 usually worked well for the FS-LA protocol.

Library amplification

We adapted the number of PCR cycles based on the amount of cDNA after the pre-amplification reaction. Tagmented pre-amplified cDNA from both hPBMCs (16 cycles) and

HEK 293T cells (12 cycles) were amplified using 14 enrichment PCR cycles. We recommend doing an initial test but, in general, 14 enrichment PCR cycles should work in most conditions. The adequate number of PCR cycles for cDNA pre-amplification and post-tagmentation depend of course on one another.

**cDNA pre-amplification (PCR cycles)**	**Library Amplification**
4 (HEK 293T)	24
6 (HEK 293T)	22
8 (HEK 293T)	18
10 (HEK 293T)	16
12 (HEK 293T)	14
[redacted]	
16 (hPBMCs)	14

Reference

1. https://www.miltenyibiotec.com/_Resources/Persistent/ca9f513c68ed01981bc4d7aa25b01c90db75e6f5/Average_RNA_yields.pdf

Supplementary Note 2

List of all the oligonucleotides used in this study

FLASH-seq

Oligo ID	Sequence (5’ → 3’)	Comments
FS dT30VN*	/5Biosg/AAGCAGTGGTATCAACGCAGAGTACTTTTTTTTTTTTTTTTTTTTTTTTTTTTTTVN	desalted or HPLC
FS TSO	/5Biosg/AAGCAGTGGTATCAACGCAGAGTACrGrGrG	

Smart-seq2

Oligo ID	Sequence (5’ → 3’)	Comments
Smart dT30VN*	/5Biosg/AAGCAGTGGTATCAACGCAGAGTACTTTTTTTTTTTTTTTTTTTTTTTTTTTTTTVN	Picell *et al.*, 2013
FS TSO	/5Biosg/AAGCAGTGGTATCAACGCAGAGTACrGrGrG	desalted or HPLC
ISPCR	/5Biosg/AAGCAGTGGTATCAACGCAGAGT	Picell *et al.*, 2013

* Please note that FS dT30VN and SMART dT30VN have the same sequence and will be used interchangeably in the main manuscript.

Smart-seq3

Oligo ID	Sequence (5’ → 3’)	Comments
SS3-Forward	TCGTCGGCAGCGTCAGATGTGTATAAGAGACAGATTGCGCAA*T*G	Hagemann-Jensen *et al.*, 2020
SS3-Reverse	ACGAGCATCAGCAGCATACGA	
SS3-oligo-dTVN	/5BiosG/ACGAGCATCAGCAGCATACGATTTTTTTTTTTTTTTTTTTTTTTTTTTTTTVN	
SS3-TSO	/5BiosG/AGAGACAGATTGCGCAATGNNNNNNNNrGrGrG	

FLASH-seq with UMI

Oligo ID	Sequence (5' → 3')	Comments
STRT-dT	/5Biosg/AATGATACGGCGACCACCGATCGTTTTTTTTTTTTTTTTTTTTTTTTTTTTTT	Hochgerner *et al.*, 2017
DI-PCR-P1A - reverse	AATGATACGGCGACCACCGA	Hochgerner *et al.*, 2017

Oligo ID	Sequence (5' → 3')	Comments
FS-TSO-UMI	/5Biosg/AAGCAGTGGTATCAACGCAGAGTNNNNNNNNrGrGrG	desalted or HPLC
TSO-CAGCA	/5Biosg/AAGCAGTGGTATCAACGCAGAGTNNNNNNNNCAGCArGrGrG	
TSO-ATAAC	/5Biosg/AAGCAGTGGTATCAACGCAGAGTNNNNNNNNATAACrGrGrG	
TSO_CTAAC**	/5Biosg/AAGCAGTGGTATCAACGCAGAGTNNNNNNNNCTAACrGrGrG	
TSO_ATGAC	/5Biosg/AAGCAGTGGTATCAACGCAGAGTNNNNNNNNATGACrGrGrG	
TSO_CTGAC	/5Biosg/AAGCAGTGGTATCAACGCAGAGTNNNNNNNNCTGACrGrGrG	
TSO_AAGCA	/5Biosg/AAGCAGTGGTATCAACGCAGAGTNNNNNNNNAAGCArGrGrG	
TSO_CATCA	/5Biosg/AAGCAGTGGTATCAACGCAGAGTNNNNNNNNCATCArGrGrG	
TSO_CGTAC	/5Biosg/AAGCAGTGGTATCAACGCAGAGTNNNNNNNNCGTACrGrGrG	
Tn5_ISPCR_F	TCGTCGGCAGCGTCAGATGTGTATAAGAGACAGAAGCAGTGGTATCAACGCAGAGT	

** TSO which we recommend and the one we used in most of our experiments.
