# Supplementary material for: Fast and highly sensitive full-length single-cell RNA sequencing using FLASH-seq
Source: Nat Biotechnol. 2022 May 30;40(10):1447–51. doi: 10.1038/s41587-022-01312-3 (PMC9546769; doi:10.1038/s41587-022-01312-3)
Supplement: Supplementary file 2 — Reporting Summary [file 41587_2022_1312_MOESM2_ESM.pdf]

nature research

Corresponding author(s): Simone Picelli

Last updated by author(s): Apr 4, 2022

Reporting Summary

Nature Research wishes to improve the reproducibility of the work that we publish. This form provides structure for consistency and transparency in reporting. For further information on Nature Research policies, see our Editorial Policies and the Editorial Policy Checklist.

Statistics

For all statistical analyses, confirm that the following items are present in the figure legend, table legend, main text, or Methods section.

n/a	Confirmed	
☐	☒	The exact sample size (*n*) for each experimental group/condition, given as a discrete number and unit of measurement
☐	☒	A statement on whether measurements were taken from distinct samples or whether the same sample was measured repeatedly
☐	☒	The statistical test(s) used AND whether they are one- or two-sided<br>*Only common tests should be described solely by name; describe more complex techniques in the Methods section.*
☐	☒	A description of all covariates tested
☐	☒	A description of any assumptions or corrections, such as tests of normality and adjustment for multiple comparisons
☐	☒	A full description of the statistical parameters including central tendency (e.g. means) or other basic estimates (e.g. regression coefficient) AND variation (e.g. standard deviation) or associated estimates of uncertainty (e.g. confidence intervals)
☐	☒	For null hypothesis testing, the test statistic (e.g. *F*, *t*, *r*) with confidence intervals, effect sizes, degrees of freedom and *P* value noted<br>*Give P values as exact values whenever suitable.*
☒	☐	For Bayesian analysis, information on the choice of priors and Markov chain Monte Carlo settings
☒	☐	For hierarchical and complex designs, identification of the appropriate level for tests and full reporting of outcomes
☐	☒	Estimates of effect sizes (e.g. Cohen's *d*, Pearson's *r*), indicating how they were calculated

Our web collection on statistics for biologists contains articles on many of the points above.

Software and code

Policy information about availability of computer code

Data collection	We used the following software to collect data: BD FACSDiva software (version 8.0.2), NextSeq control software (version 4.0.1), Agilent 2100 Bioanalyzer Expert (version B.02.10.51764), bcl2fastq v2.20.0.422
Data analysis	Data Preprocessing (trimming, mapping, read assignment to a feature and read distributions, ...) were performed with:<br>BBMAP (v38.86), Umi-tools (v1.1.1), STAR (v2.7.3), samtools (v1.10), featureCounts (v2.0.0), ReSQC (v4.0.0), Salmon (v1.5.2), seqkt (v1.3-r106.), trimmomatic (v0.39), Cellranger (v6.1.1), bwa (v0.7.17-r1188), Picard v2.23.8-2-ga004f14-SNAPSHOT or v2.26.5), GATK (v4.2.2.0 or v4.1.4.1), bcftools (v1.10.2), python (v3.8.8)<br>Data analysis was performed in R (v4.1.0) with the following key libraries:<br>pcaPP (v1.9-74), rstatix (v0.7.0), tximport (v1.20.0), Seurat (v4.0.3), SCTransform (v0.3.2), uwot (v0.1.10), BSgenome (v1.60.041), BSgenome.Hsapiens.UCSC.hg38 (v1.4.3), ggseqlogo (v0.1), SoupX (v1.5.2), DoubletFinder (v2.0.3), bamSignals (v1.24.0), vcfR (v1.12.0) Custom scripts/code are accessible at: https://github.com/vincenthahaut/FLASH-Seq.

For manuscripts utilizing custom algorithms or software that are central to the research but not yet described in published literature, software must be made available to editors and reviewers. We strongly encourage code deposition in a community repository (e.g. GitHub). See the Nature Research guidelines for submitting code & software for further information.

Data

Policy information about availability of data

All manuscripts must include a data availability statement. This statement should provide the following information, where applicable:

- Accession codes, unique identifiers, or web links for publicly available datasets
- A list of figures that have associated raw data
- A description of any restrictions on data availability

Sequencing data are related to HEK 293T cells and hPBMCs have been deposited to Sequence Read Archive (PRJNA816486).
Count tables, other processed data and cDNA yields associated with this study are available on Mendeley data (doi: 10.17632/bh47n6fnpd.1).
Scripts used to process the data are available at: https://github.com/vincenthahaut/FLASH-seq.

Field-specific reporting

Please select the one below that is the best fit for your research. If you are not sure, read the appropriate sections before making your selection.

☒ Life sciences ☐ Behavioural & social sciences ☐ Ecological, evolutionary & environmental sciences

For a reference copy of the document with all sections, see nature.com/documents/nr-reporting-summary-flat.pdf

Life sciences study design

All studies must disclose on these points even when the disclosure is negative.

Sample size

FLASH-seq / FLASH-Seq low amplification Protocol Development
While testing the different reaction conditions for the FLASH-seq protocol (see extended file #1), we sequenced at least 16 HEK 293T cells per condition.
Our goal was to observe if the tested conditions presented large differences in the ability of the method to detect an increased number of expressed genes while showing similar mapping statistics (uniquely mapped reads, intronic / exonic / intergenic reads, ...).
HEK 293T cells are homogenous, and are regularly used for this purpose. We selected 16 cells as a minimum based on prior publications (e.g., Hagemann-Jensen et al 2020, Nature Biotechnology, Extended Data Fig. 1) which typically used 8 to 16 HEK 293T cells for protocol development.
We validated this decision by comparing 100-times the number of genes detected in 16 randomly selected HEK 293T cells (FS-5µL, 250K raw reads) with a simulated number of genes for 16 other HEK 293T cells which would harbor a 1, 2, 3, ... 10% drop in mean±SD genes detected (Wilcoxon rank sum test, two-sided, P-value). Statistically significant changes can be observed with a gain >1% or a loss of >4% of the expressed genes. While in theory this setting would adequately capture small changes, only larger differences were of interest for our purposes (e.i., >10%).

Retinal Organoids
The rarest classes of cells (e.g., cones and horizontal) are usually found at ~2.5 to 5% of the total cells. Each of these classes is not homogenous, containing at least a few similar but still distinct cell types. Based on these numbers and our past experience with PBMC and retinal organoids (Cowan et al, Cell, 2020), we processed 1536 cells with FLASH-seq and ~8000-10,000 cells with 10x Genomics, in order to capture a representative number of cells from even the rare cell types.

Data exclusions

Single-cell data was filtered according to established criteria to remove technically failed cells. Relevant sequencing parameters are listed in the Methods section.

Replication

Experiments were performed across hundreds of cells and using different cell types. All the experimental conditions that did not work or could not be reproduced were not pursued further. All the remaining experimental conditions were validated multiple times and across 2 or more cell types.

Randomization

FACS sorting is already randomly depositing individual cells into separate wells of microplates. As this is the first step in the protocol, other sources of randomization were not needed/not relevant.

Blinding

Investigators were not blinded to groups of samples. Knowing which samples were processed with which protocol is unavoidable, if the goal is to assess the best experimental conditions for a new protocol.

Reporting for specific materials, systems and methods

We require information from authors about some types of materials, experimental systems and methods used in many studies. Here, indicate whether each material, system or method listed is relevant to your study. If you are not sure if a list item applies to your research, read the appropriate section before selecting a response.

Materials & experimental systems

n/a	Involved in the study
[ ]	[x] Antibodies
[ ]	[x] Eukaryotic cell lines
[x]	[ ] Palaeontology and archaeology
[x]	[ ] Animals and other organisms
[ ]	[x] Human research participants
[x]	[ ] Clinical data
[x]	[ ] Dual use research of concern

Methods

n/a	Involved in the study
[x]	[ ] ChIP-seq
[ ]	[x] Flow cytometry
[x]	[ ] MRI-based neuroimaging

Antibodies

Antibodies used

FITC-conjugated Mouse Anti-Human CD45 Antibody used at a final dilution of 1:50 (Clone HI30, BD Biosciences, cat. # 560976).

Validation

Commercially available from BD Biosciences and routinely tested.

Eukaryotic cell lines

Policy information about cell lines

Cell line source(s)

HEK 293T (ATCC, CRl-3216); human retinal organoids were generated from the IPSC line 01F49i-N-B7 at passage 39. The iPSC line 01F49i-N-B7 was derived from anonymized donor tissue (gender: female).

Authentication

HEK 293T: commercially available from ATCC and authenticated by the vendor. Human retinal organoids: cell identity was confirmed by short tandem repeat analysis (STR analysis; Microsynth, Switzerland) on genomic DNA extracted from fibroblasts and iPSCs by the DNeasy Blood & Tissue Kit (QIAGEN).

Mycoplasma contamination

Negative (routinely tested).

Commonly misidentified lines
(See ICLAC register)

HEK 293T were used but authenticity was confirmed (see above).

Human research participants

Policy information about studies involving human research participants

Population characteristics

N.A., as these are anonymized blood samples.

Recruitment

- Anonymized blood samples from the Red Cross Blood Bank Hospital of Basel (Switzerland). We did not select for any specific age, gender, ethnicity or other factors but just received what was available at the time or request. All samples were tested negative for pathogens.
- Commercial Human Mononuclear Cells from adult individuals (Sigma, 690PB-100A).

Ethics oversight

Anonymized blood samples
According to the Swiss Federal Act on Research involving Human Beings of 30 September 2011 (Status as of 26 May 2021) work with anonymized biological material does not require approval by the ethics commission. Samples are irreversibly anonymized by assigning an internal identifier upon receipt of the sample and no records of the sample identifier given by the provider are kept (https://www.fedlex.admin.ch/eli/cc/2013/617/en)

Human retinal organoids
Tissue samples are fully anonymized, were obtained in accordance with the tenets of the Declaration of Helsinki and all experimental protocols were approved by the local ethics committee (Ethikkommission Nordwest- und Zentralschweiz (EKNZ)). All the relevant information, including Ethics are described on: https://hpscreg.eu/cell-line/IOBi001-A

Note that full information on the approval of the study protocol must also be provided in the manuscript.

Flow Cytometry

Plots

Confirm that:

☒ The axis labels state the marker and fluorochrome used (e.g. CD4-FITC).

☒ The axis scales are clearly visible. Include numbers along axes only for bottom left plot of group (a 'group' is an analysis of identical markers).

☒ All plots are contour plots with outliers or pseudocolor plots.

☒ A numerical value for number of cells or percentage (with statistics) is provided.

Methodology

Sample preparation	Detailed information is reported in the Methods section. In brief:<br>- hPBMC: leucocytes were isolated from whole blood by density gradient centrifugation using Ficoll-Paque. Prior to FACS sorting, cells were resuspended in 1x PBS+ 2% FBS and stained with a FITC311 conjugated Mouse Anti-Human CD45 Antibody for 25 min on ice. Unbounded antibodies were washed away by adding 3 ml of Roswell Park Memorial Institute 1640 medium (RPMI) + 2% FBS, cells were centrifuged for 5 min at 300 x g, resuspended in 1x PBS+ 0.04% BSA, and strained through a 40-µm filter then stained for 5 min with Propidium Iodide at room temperature to label dying cells.<br>- HEK 293T cells were cultivated in Dulbecco's Modified Eagle Medium (DMEM) supplemented with 10% FBS and 1% penicillin/streptavidin. Prior to FACS-sorting cells were centrifuged for 5 min at 300 x g, resuspended in 1x PBS+ 0.04% BSA, and strained through a 40-µm filter then stained for 5 min with Propidium Iodide at room temperature to label dying cells.<br>- Human retinal organoids: IPSCs were cultured in mTesrl medium (STEMCELLTechnologies) at 37°C, 5% CO2. Embryoid bodies were generated from an initial IPSC concentration of 300 cells/microwell. At week 18, retinal organoids were selected based on the presence of outer segments and characteristic retinal layers, pooled together and washed once with 1 ml of Ringer solution without calcium, pre-warmed at 37°C. The Neural Tissue Dissociation Kit P (Miltenyi Biotec) was used to dissociate them into single cells. After removing the Ringer solution, an enzymatic dissociation mix composed of 50 µl of Enzyme P and 975 µl of Buffer X was added. The tube was incubated on a SmartBlock thermoblock (Eppendorf) at 37°C with gentle agitation (500 rpm). The solution was mixed with a wide-bore P1000 pipette at regular intervals to facilitate cell dissociation. The reaction was stopped as soon as the organoids showed signs of dissociation (~20-30 min) by adding 15 µl of Stop solution (5 µl Enzyme A+ 10 µl Buffer Y). Samples were mixed gently by inversion and returned to the thermoblock for 15 min. After this time, a last round of trituration with a wide-bore P1O00 pipette helped break apart the remaining cell aggregates. The cell suspension was briefly put on ice before a quick spin for 5 min at 300 x gin a pre-refrigerated (4°C) centrifuge (Eppendorf). The supernatant was carefully removed without disturbing the pellet, before performing one wash with 1x PBS pH 7.4 + 0.04% BSA (PBS: Thermo Fisher; MACS BSA: 10% solution, Miltenyi Biotec). Supernatant was removed again and cells were resuspended in 1x PBS pH 7.4 + 0.04% BSA and sequentially strained through a 70-µm and then a 40-µm filter (pluriSelect). Viability staining was carried out with Propidium Iodide (1 mg/ml, ThermoFisher) at room temperature for 5 min to label dying cells.
Instrument	FACSAria Fusion (100-um nozzle).
Software	BD FACSDiva software (version 8.0.2).
Cell population abundance	HEK293 cells were generally 80-95% alive after thawing, washing and counting.<br>PBMC were cryopreserved and viability after thawing was >95%.<br>Human retinal organoid cells were processed fresh. Viability was >90% after dissociation.
Gating strategy	Particles smaller than cells (debris) were eliminated with an area plot of Forward-Scatter (FSC-A) versus Side-Scatter (SSC-A) by gating for cell-sized particles inside the gate. A 2-steps doublet discrimination was carried out to remove cell aggregates by making plots of Area versus Height, both in the Forward- and Side-Scatter channel, FSC-A vs FSC-H and SSC-A vs SSC-H, respectively. For the hPBMCs we plotted FITC-H (for CD45) vs TexasRed-H (for PI), sorting all singlets that were FITC+PI- (lower-right panel). For HEK 293T and organoid cells we didn't use antibodies and vsorted eerything that was non-debris, single-cell (2-ways doublet removal strategy) and PI- (live cell).

☒ Tick this box to confirm that a figure exemplifying the gating strategy is provided in the Supplementary Information.
